# Integrative multi-omics framework for causal gene discovery in Long COVID

**DOI:** 10.1371/journal.pcbi.1013725

**Published:** 2025-12-01

**Authors:** Sindy Pinero, Xiaomei Li, Lin Liu, Jiuyong Li, Sang Hong Lee, Marnie Winter, Thin Nguyen, Junpeng Zhang, Thuc Duy Le

**Affiliations:** 1 UniSA STEM, University of South Australia, Adelaide, South Australia, Australia; 2 Agriculture and Food Institute, Commonwealth Scientific and Industrial Research Organisation, Marsfield, New South Wales, Australia; 3 Australian Centre for Precision Health, University of South Australia, Adelaide, South Australia, Australia; 4 UniSA Allied Health and Human Performance, University of South Australia, Adelaide, South Australia, Australia; 5 South Australian Health and Medical Research Institute (SAHMRI), University of South Australia, Adelaide, South Australia, Australia; 6 Future Industries Institute, University of South Australia, Adelaide, South Australia, Australia; 7 Applied Artificial Intelligence Institute, Deakin University, Melbourne, Victoria, Australia; 8 School of Engineering, Dali University, Dali, Yunnan, China; BioInnovation Institute, DENMARK

## Abstract

Long COVID, or Post-Acute Sequelae of SARS-CoV-2 infection (PASC), affects an estimated 10–20% of COVID-19 patients and presents persistent multisystemic symptoms. Although demographic and clinical factors, such as age, sex, and comorbidities, contribute to risk, the genetic mechanisms underlying this risk remain poorly defined. To address this gap, we developed a multi-omics framework that integrates Transcriptome-Wide Mendelian Randomization (TWMR), Control Theory (CT), Expression Quantitative Trait Loci (eQTL), Genome-Wide Association Studies (GWAS), RNA sequencing (RNA-seq), and Protein-Protein Interaction (PPI) network to identify putative causal genes and network drivers in Long COVID. Our approach prioritized 32 candidate genes, including 19 previously reported and 13 novel, with roles in the SARS-CoV-2 response, viral carcinogenesis, immune regulation, and cell cycle control. Enrichment analyses revealed a shared genetic architecture in syndromic, metabolic, autoimmune, and connective tissue disorders. Using causal gene expression profiles, we identified three distinct symptom-based subtypes of Long COVID, providing information on the heterogeneity of disease mechanisms and clinical presentation. Finally, we developed an open-source Shiny application for interactive exploration of these findings. Together, this integrative framework highlights novel causal mechanisms and therapeutic targets, advancing precision medicine strategies for Long COVID.

## Author summary

We developed a computational approach to understand why some individuals experience long-lasting symptoms after COVID-19 infection, a condition known as Long COVID that affects millions worldwide. Although physicians can identify patients with Long COVID, we do not yet fully understand which genes cause it or how to treat it effectively. We combined two powerful analytical methods to solve this problem: one that determines whether specific genes cause Long COVID (rather than just being associated with it), and another that identifies key control points in biological networks. Our analysis of genetic and molecular data identified 32 genes that are likely to cause Long COVID, including 13 that have not been previously linked to the condition. We discovered that Long COVID consists of three distinct subtypes in terms of different gene expression profiles, each with distinct symptoms and underlying biology. We also found that Long COVID shares genetic factors with other conditions, such as autoimmune and metabolic disorders, which may explain its diverse symptoms. To help other researchers and physicians employ our findings, we have created a free online tool that enables them to explore the data and potentially identify new treatment targets. Our work provides the first comprehensive genetic framework for understanding Long COVID and developing personalized treatments.

## Introduction

Long COVID, also known as Post-Acute Sequelae of COVID-19 (PASC), is a complex condition characterized by the persistence or onset of symptoms after SARS-CoV-2 infection. Long COVID is defined differently by various organizations. For example, the World Health Organization (WHO) and the Centers for Disease Control and Prevention (CDC) describe it as symptoms that persist three months after infection and last at least two months [1,2]. In contrast, the National Institute for Health and Care Excellence (NICE) considers it to start as early as one month after infection [3–6]. Regardless of the definition timing, key risk factors include age, sex, ethnicity, socioeconomic status, vaccination status, smoking, and underlying health conditions [7]. In addition, studies have linked various biomarkers to Long COVID, particularly those related to inflammation, immune dysfunction, and coagulation abnormalities [8].

Despite significant progress in identifying risk factors and clinical markers [7,8], understanding the role of gene expression as a causal factor in Long COVID remains a major challenge. This knowledge gap presents a significant barrier to the development and implementation of interventions and targeted therapies [9], highlighting the need for novel approaches that focus on gene expression patterns associated with Long COVID. Identifying these Long COVID-causing genes is essential for advancing targeted treatment strategies. It can also improve diagnostic accuracy and promote better monitoring and prediction of patient outcomes [10].

Computational methods for identifying disease-causing genes typically employ two primary strategies, each offering distinct advantages that complement each other. The first strategy aims to identify genes associated with disease risk and prevention, often using approaches such as Transcriptome-Wide Mendelian Randomization (TWMR) [11]. The TWMR method incorporates transcriptomic data into MR studies utilizing genetic variants that influence gene expression, such as Quantitative Expression Trait Loci (eQTLs), to establish causal relationships between gene activity and disease. The TWMR methodology can identify whether altered gene expression directly influences an outcome (in this case, Long COVID) and reveal potential therapeutic targets. The resulting analysis reveals which genetic factors influence disease susceptibility or protection through genetic associations and causal inference, allowing researchers to identify specific genetic variants with direct causal effects on diseases. However, TWMR analysis often requires strong genetic instruments (e.g., single-nucleotide polymorphisms (SNPs) that robustly modulate gene expression), and determining causal relationships becomes more complex when confounding variables or pleiotropy are present.

The second strategy identifies genes or proteins that are crucial in biological networks. Techniques such as Bayesian Networks [12], Node Importance [13], and Control Theory (CT) [14] are used to understand how different genes and proteins interact within biological pathways, considering the interconnected nature of biological systems. CT is particularly useful for identifying critical nodes or key genes and proteins that significantly influence the entire network. Identifying these critical nodes (network driver genes) enables researchers to determine which components would be the most effective therapeutic targets for stabilizing or controlling disease-related disturbances. For example, CT methods have been utilized in cancer research to identify key regulatory genes, such as *TP53*, whose modulation can restore network stability, thus providing focused therapeutic opportunities [15].

In this study, we propose a novel framework to explore and discover potential genes involved in Long COVID by integrating two complementary strategies: MR [11] and CT [14], along with multi-omics data, including eQTLs, Genome-Wide Association Studies (GWAS), RNA sequencing (RNA-seq), and the human Protein-Protein Interaction (PPI) network. Our approach identifies candidate causal genes that may contribute to Long COVID risk and examines their potential regulatory roles within a network. Specifically, we discover genes whose expression patterns suggest either increased susceptibility to Long COVID or a crucial role in maintaining biological network stability. By integrating these methodologies and utilizing multi-omics data, our analysis provides comprehensive insights into the potential genetic mechanisms underlying Long COVID, highlighting candidate therapeutic targets for further investigation.

Our study has identified 32 potential causal genes for Long COVID, of which 19 have been confirmed by the existing literature, providing support for the effectiveness of our findings. The remaining genes represent promising candidates for follow-up experiments. Among these candidates, we identified genes that act as risk or protective factors, as well as network driver genes that regulate and stabilize the disease network’s structure. Enrichment analyses revealed important biological pathways in Long COVID, including SARS-CoV-2 infection, viral carcinogenesis, cell cycle regulation, and immune response mechanisms. Using the identified potential causal genes, we clustered Long COVID patients into three distinct subtypes with different symptom profiles, establishing a foundation for personalized diagnostic and therapeutic approaches. This work represents a significant step toward customized management and treatment strategies for Long COVID, ultimately improving patient outcomes.

To facilitate the application of our framework, we developed a web application (a Shiny app) that allows users to generate gene lists by adjusting parameters for direct (MR) and network-based (CT) causal approaches. This tool provides researchers and clinicians with an accessible platform to explore parameter variations and analyze their data, enhancing the reproducibility of our findings.

## Materials and methods

### Overview of the causal gene discovery framework

**Fig 1:**
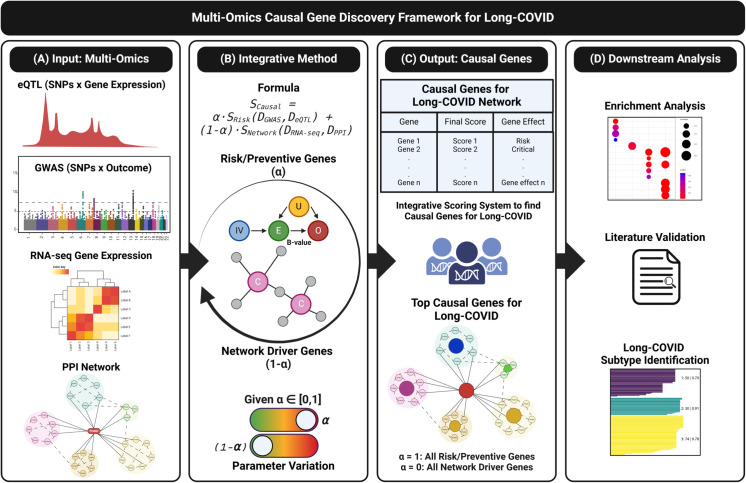
A causal gene discovery framework for Long COVID using multi-omics data. (A) The input data includes expression Quantitative Trait Loci (eQTL), Long COVID Genome-Wide Association Studies (GWAS), RNA sequencing (RNA-seq), and the human Protein-Protein Interaction (PPI) network. (B) A fusion approach to evaluating gene expression by integrating Transcriptome-Wide Mendelian Randomization (TWMR) and Control Theory (CT) scores. (C) Significant genes are ranked by their weighted scores. (D) Downstream analyses include Enrichment Analysis (EA), literature review, and the identification of Long COVID subtypes. SNPs: Single Nucleotide Polymorphisms, IVs: Instrumental Variables. E: Exposure. O: Outcome. U: Confounders. Created in BioRender. Piñero, S. (2025) https://BioRender.com/6awyup6.

The causal gene discovery framework integrates various data sources, including eQTL, GWAS, RNA-seq, and PPI networks, to identify genes with putative causal roles in Long COVID (Fig 1). It begins by processing multi-omics input data (Fig 1A) and then applies an integrative scoring method (Fig 1B) that combines TWMR with CT-based network analysis. This approach balances the contributions of risk and protective factors and of genes critical to the network through a parameter (*α*) that can be adjusted to accommodate both goals. The output (Fig 1C) ranks putative causal genes by weighted scores, offering insights into their roles within the Long COVID network. Finally, downstream analyses (Fig 1D), including Enrichment Analysis (EA), literature validation, and subtype identification, help discover disease mechanisms and prioritize therapeutic targets. This comprehensive computational approach integrates genetic and network-based perspectives, providing deeper insights into the nature of Long COVID.

By treating genetic variants as instrumental variables (IVs), two-sample MR methods estimate the effects of genetically regulated risk exposures for complex diseases using only summary statistics. When considering gene expression as exposure in TWMR analyses, we aim to identify gene expressions that have causal relationships with the disease of interest. In our case, we focus on identifying the genes that act as risk or protective factors for Long COVID. Given the limited number of eQTLs available as IVs for a gene, which makes it challenging to detect invalid IVs, we adopt the multi-tissue approach, *Mt-Robin* [11]. This method uses eQTL data in a mixed model to identify IV-specific random effects arising from pleiotropy due to estimation errors in the eQTL summary statistics, allowing accurate inference of the dependence (fixed effects) between eQTL and GWAS effects, even in the presence of invalid IVs.

Although MR approaches identify genes that directly affect Long COVID, network biology approaches, such as CT, have shown that the genes driving the disease are not limited to those directly linked to the disease phenotypes [14]. In this work, we employ a CT approach to extract a list of genes that serve as network drivers for Long COVID—i.e., genes whose removal or intervention would disrupt the biological networks associated with the disease, thereby affecting disease outcomes. These network driver genes may or may not have direct causal relationships with the disease.

To create a comprehensive list of putative causal genes for Long COVID and to understand their roles in disease regulation, we use a fusion approach that integrates the two methods described above (see Framework subsection for details). Specifically, we calculate the scores for each gene using the following formula:


$$S_{\text{Causal}}=\alpha \cdot S_{\text{Risk}}(D_{\text{GWAS}},D_{\text{eQTL}})$$

$$+(1-\alpha )\cdot S_{\text{Network}}(D_{\text{RNA-seq}},D_{\text{PPI}})$$ (1)

where:

- $S_{\text{Causal}}$ represents the final score of each gene.
- $S_{\text{Risk}}(D_{\text{GWAS}},D_{\text{eQTL}})$ is the score derived from the TWMR approach (*Mt-Robin*) to identify putative causal risk and protective genes using GWAS and eQTL datasets.
- $S_{\text{Network}}(D_{\text{RNA-seq}},D_{\text{PPI}})$ is the CT approach score to identify network driver genes based on RNA-seq data and the human PPI network.
- The parameter α controls the contribution of each risk/protective putative causal gene, while 1−α adjusts the influence of each network-critical gene.

The formula (1) integrates an approach that ranks genes by combining their causal effects and significance within the Long COVID network, providing a comprehensive prioritization based on both causal and network properties.

Thus, this causal multi-omics approach provides insights into the genetic mechanisms underlying Long COVID and highlights potential intervention targets.

### Dynamic visualization of Long COVID putative causal genes: A shiny application

In our model, the parameter *α* serves as an adjustable coefficient that enables researchers to explore different scenarios for prioritizing protein-coding genes based on their roles in influencing disease risk or prevention and controlling the Long COVID network.

As *α* approaches 1, the model prioritizes genes associated with disease risk and protective scenarios. These genes are directly associated with the pathogenesis of Long COVID, highlighting potential therapeutic targets for intervention.

In contrast, as *α* approaches 0, the model emphasizes driver genes critical to the structure of the disease network. These genes regulate key interactions within the network, positioning them as potential therapeutic targets to restore lost stability or modulate pathological states.

The model integrates both perspectives across the intermediate range of *α*, balancing network controllability and disease risk. In this case, genes that significantly influence the network and are closely related to disease risk become key players, making them important targets for further investigation.

Researchers can dynamically explore these shifts in gene rankings by adjusting *α* in our interactive tool available at https://sindypin.shinyapps.io/github/. This instrument allows a detailed examination of how genes transit from disease risk or protective factors (α→1) to network drivers (α→0).

### Input data collection and preparation

The success of our integrative multi-omics framework relies on the careful selection and preparation of diverse datasets that capture Long COVID’s genetic, transcriptomic, and proteomic dimensions. We collected and curated high-quality data from publicly available resources, ensuring robust coverage of key biological processes. These datasets include cis-eQTL information from the Genotype-Tissue Expression (GTEx) project [16], GWAS findings for Long COVID susceptibility [17], Whole Genome Sequencing (WGS) for Linkage Disequilibrium (LD) analysis [16], and gene-level data from Ensembl [18]. Additionally, an RNA-seq dataset [19] and the human PPI network were incorporated to provide a comprehensive view of gene expression and functional interactions. The following subsections detail the sources, characteristics, and preparation steps for each dataset used in our analysis.

#### Expression Quantitative Trait Loci (eQTL).

We utilized 49 significant cis-eQTL datasets, each within a 1Mb region and meeting a False Discovery Rate (FDR) threshold of < 0.05, obtained from the GTEx project (Version 8, Ensembl 99, GRCh38) [16]. These datasets comprise 39,832 unique genes derived from nearly 1,000 healthy European individuals, accessed on 9 August 2023. They were crucial for investigating the relationship between genetic variation and gene expression across different human tissues (S1 Text). For more details and a description of the datasets available in the GTEx consortium, refer to the original publication [20].

#### Genome-Wide Association Studies (GWAS).

We sourced a Long COVID GWAS dataset (Release 7; Ensembl 109; HGB GRCh38) from Lammi et al., 2025 [17]. This dataset consists of 3,018 cases evaluated for 19 symptoms three months post-COVID-19 infection according to WHO and CDC definitions of Long COVID [1,2], and 1,093,995 broad controls from the general population who were not specifically evaluated for post-COVID symptoms across six ancestries. For comprehensive details, including the complete list of ancestries, symptoms, and unique SNPs, please refer to S2 Text.

#### Whole Genome Sequencing (WGS).

To ensure the robustness and validity of our method, we calculated the LD matrix using GTEx WGS BAM files (Ensembl 88, GRCh38), which contain 820,792 unique SNPs from 836 male and female European individuals (S3 Text). Access to this specific dataset was granted through special permission [16].

For the calculation of the LD matrix, we utilized GTEx-EUR BAM files as our primary reference panel [16]. Users of our method can replace this reference with ancestry-matched panels as needed for their specific research contexts, thereby improving the precision of LD estimation in non-European populations.

#### Human genes dataset.

To assess the causal relationship of each gene with the outcome, we utilized the public Human Genes dataset from the Ensembl Genes database (version 110, GRCh38), which contains 70,116 genes [18].

#### RNA Sequencing (RNA-seq).

Moreover, we analyzed RNA-seq gene expression data from the Mount Sinai COVID-19 Biobank Study [21]. The dataset comprises patients with Long COVID symptoms (persisting for more than one month post-acute infection, following established institutional criteria [4–6]), COVID-19 patients, and healthy controls. We sourced this dataset from the Gene Expression Omnibus - National Center for Biotechnology Information (GEO-NCBI) database, under the identifier GSE215865, corresponding to the Ensembl GRCh37 release [19]. It contains 413 blood samples from 158 individuals with Long COVID (S4 Text).

#### Protein-Protein Interaction (PPI).

Finally, we employed the human PPI dataset published by Vinayagam et al., 2011 [22] as a model to build the Long COVID network (S5 Text).

### Framework

To create a comprehensive list of putative causal genes for Long COVID and to understand their roles in disease regulation, we used a fusion approach integrating MR and CT. Specifically, we calculated the scores of each gene using the formula in Eq 1. This approach produced a final ranking of genes based on their direct causal relationships and significance within the Long COVID network. The following sections detail the calculations of $S_{\text{Risk}}$ and $S_{\text{Network}}$

#### Calculating $S_{\text{Risk}}$.

To calculate $S_{\text{Risk}}$, we employed the *Mt-Robin* method [11] to identify genes that act as risk or protective factors for Long COVID. Using GWAS (${𝐃}_{\text{GWAS}}$) and eQTL (${𝐃}_{\text{eQTL}}$) data (see the Overview Section), this approach accurately infers the dependence (fixed effects) between eQTL and GWAS effects, even with potential invalid IVs.

We first constructed and refined the LD matrix using SNPs from our dataset to ensure robust genetic instruments. We calculated pairwise *r*^2^ values and applied an LD 0.5 threshold to filter highly linked SNPs. Our multi-criteria SNP selection process eliminated those with multiple correlations above the LD threshold, prioritized SNPs present across multiple tissues with consistent effect directions, and selected significant SNPs with the smallest minimum p-values. In addition, we required genes to be expressed in at least one tissue.

Statistical analysis involved reverse regression coefficients and weighted regression with random slopes and correlated errors. We integrated these results with GWAS standard errors and the refined LD matrix to inform our resampling strategy. We evaluated causal relationships using bootstrapping to generate null distributions while preserving the SNP LD structure. We resampled GWAS effect sizes for each gene, preserving LD correlations, and calculated test statistics under the null hypothesis of no association. The p-value for each gene was determined by the proportion of null test statistics exceeding the observed value, excluding samples with non-convergence or singular fits in the mixed-effects model.

The final score was calculated using the absolute effect size ($\beta_{y}$) from the MR method. Genes with a p-value or FDR greater than 0.05 received a score of 0, ensuring only significant causal effects. We normalized the MR score ($S_{\text{Risk}}$) using min-max scaling for cross-gene comparability:


$$S_{\text{Risk}}=S_{\text{MR\_norm}}$$

$$=\frac{S_{\text{MR}}-\min\{S_{\text{MR}}\}}{\max\{S_{\text{MR}}\}-\min\{S_{\text{MR}}\}}$$ (2)

where $S_{\text{MR}}$ represents the size of each gene’s causal effect, and $\min(S_{\text{MR}})$ and $\max(S_{\text{MR}})$ are the smallest and largest values in all genes, respectively.

Finally, we estimated FDR-corrected p-values to identify significant putative causal contributors to Long COVID (p-value < 0.05).

#### Calculating $S_{\text{Network}}$.

To calculate $S_{\text{Network}}$, we integrated RNA-seq expression data from Long COVID patients and the human PPI network described in the Overview Section. RNA-seq data revealed disease-specific gene expression patterns, while the PPI network provided structural relationships among proteins. Together, these datasets allowed us to identify and classify driver nodes that control the biological network underlying Long COVID.

Network analysis involved constructing a directed graph in which nodes represent genes and edges indicate protein interactions. We first mapped the RNA-seq expression data to the PPI network to identify which genes were expressed in Long COVID patients and how they interacted. This integration allowed us to assess the control-theoretic properties of each gene within the specific context of Long COVID, rather than in a general PPI network.

We then classified genes by removing each from the network and observing changes in the number of required driver nodes needed for control [14]. This process identified three categories: indispensable genes (requiring an increase in driver nodes), neutral genes (showing no significant change), and dispensable genes (exhibiting minimal impact). We focused our analyses on indispensable genes due to their critical role in maintaining network control. Dispensable genes were excluded from further analysis because they do not significantly contribute to the network’s control structure and would not decrease the number of drivers required for full network controllability.

We further refined indispensable genes into Type-I and Type-II classifications based on their network behavior. Type-I genes were categorized based on their effects on other driver nodes. Critical genes were those whose removal increased the number of required driver nodes, particularly by disrupting directed paths connecting regulatory nodes to their downstream targets. Redundant genes reduced the number of required driver nodes, whereas ordinary genes did not.

Type-II genes were classified according to their control requirements: critical genes (zero in-degree, ${\text{K}}_{\text{in}}=0$) appeared in all driver node sets, redundant genes in none, and ordinary genes in some but not all.

We analyzed network connectivity using three measures: K (total degree), which represents total interactions and indicates network centrality; *K*_*in*_ (in-degree), which shows incoming interactions that other genes could regulate; and *K*_*out*_ (out-degree), which indicates outgoing interactions that influence different genes.

To address potential bias concerns in our network analysis, we emphasize that our approach ranks genes based on their total degree (K) and functional classification, rather than favoring genes in larger network structures. This approach ensures that genes are prioritized based on their individual network properties and control-theoretic importance, objectively identifying the most influential nodes regardless of local network density.

The CT score ($S_{\text{CT}}$) incorporated these classifications with weighted importance. Type-I critical genes were assigned a weight of 1 because they are essential for network stability. Type-II critical genes received a weight of 2 as they must always be controlled (${\text{K}}_{\text{in}}=0$). Redundant and ordinary genes received a weight of 0, reflecting their non-critical roles.

We calculated $S_{\text{CT}}$ by multiplying the total degree of each gene (K) by its assigned weighted score (W):

$$S_{\text{CT}}=K\times W$$ (3)

where $S_{\text{CT}}$ represents the network impact score for each gene calculated from its degree and weight.

The final score was normalized using the min-max scaling as follows:


$$S_{\text{Network}}=S_{\text{CT\_norm}}$$

$$=\frac{S_{\text{CT}}-\min\{S_{\text{CT}}\}}{\max\{S_{\text{CT}}\}-\min\{S_{\text{CT}}\}}$$ (4)

where $\min(S_{\text{CT}})$ and $\max(S_{\text{CT}})$ are the smallest and largest values across all genes, respectively.

#### Polygenic risk score integration analysis.

To enhance the translational impact of our findings, we integrated our 32 Long COVID putative causal genes with existing COVID-19 PGS datasets from the PGS Catalog [23]. We analyzed three available COVID-19 PGS datasets: PGS002272 (6 genome-wide significant variants), PGS002273 (12 genome-wide significant variants), and PGS004938 (955,417 variants using the LDpred2 method [24]), representing the current state of COVID-19 genetic risk prediction models.

We employed transcription start site (TSS)-based mapping with LD clumping (a 200kb window) and a conservative nearest-gene assignment (±50kb window) to map PGS variants to genes. For PGS004938, we applied the 97.5th percentile filtering to retain high-confidence variants. Gene mapping used UCSC RefSeq annotations (GRCh38) with strand-aware TSS positioning [25]. Statistical enrichment was assessed using Fisher’s exact test to determine whether the overlap between PGS and Long COVID genes exceeded the expected by chance. The distance analysis calculated the minimum distance from each Long COVID gene’s TSS to the nearest COVID-19 PGS variant. Complete methodological details and sensitivity analyses are provided in S6 Text.

#### Analysis of shared genetic basis between Long COVID and related conditions.

Disease-gene associations were compiled using five complementary databases: MalaCards [26], DISEASES [27], DISGENET [28], MedGen [29], and GenCC [30]. We systematically queried these databases for conditions associated with our identified genes, focusing on pathophysiological features that overlapped with Long COVID manifestations. Selection criteria included: (1) presence of immune/inflammatory components, (2) chronic/persistent symptoms, (3) multi-system involvement, and (4) metabolic or endocrine disruption. Conditions were categorized based on their primary pathophysiological mechanisms and potential relevance to the pathogenesis of Long COVID. The selection of the database was based on a comprehensive coverage of rare and common conditions, including mechanistic annotations and regular curation of disease-gene relationships. The complete dataset of conditions and their database sources is provided in S7 Table.

#### Enrichment Analysis (EA).

Our study conducted a comprehensive pathway EA on the risk, protective, and network driver genes identified from our framework. The aim was to identify the Biological Processes (BP), Cellular Components (CC), and Molecular Functions (MF) that are significantly associated with these genes. To ensure compatibility with various bioinformatics tools, we initially mapped Ensembl gene IDs to Entrez gene IDs using the org.Hs.eg.db database [18].

For the EA, we applied the 32 genes identified by our framework, which included all overlapping and non-overlapping genes from analyses conducted at multiple *α* parameter settings (α=0,0.25,0.50,0.75,1). The genes derived from the Mt-Robin analysis included all significant genes, whereas the CT analysis produced the top 16-ranked genes based on their network properties. This selection approach ensured that our EA captured the biological processes associated with genes identified across the entire spectrum of our computational framework, from purely statistical causal inference (α=0) to a purely network-based (α=1) approach.

We utilized well-established databases (GO [31], KEGG [32], and Reactome [33]). We prioritized enriched pathways based on statistical significance and their relevance to the established literature on Long COVID. The pathways were considered significant when they met all threshold criteria (p-value, p-adjusted, and q-value < 0.05).

Furthermore, we examined the Long COVID context by conducting a comprehensive literature review to identify potential symptoms associated with each enriched pathway, providing additional insights into the disease’s possible clinical implications.

We visualized the results using dot and network plots, which clearly and intuitively represented enriched terms and molecular pathways.

#### Gene expression clustering.

We investigated Long COVID subtypes using gene expression data from the risk, protective, and network driver genes we identified. We determined the optimal number of clusters using the CancerSubtype package’s ConC algorithm [34], an unsupervised method for subtype discovery. The analysis utilized RNA-seq data, as detailed in the Input Data Section. Moreover, we performed a grid search across hyperparameters, evaluating 2 to 5 clusters with a fixed seed of 5 for reproducibility.

After optimizing the clustering parameters, we grouped patients with Long COVID using the selected CC configuration. The cluster quality assessment involved calculating the silhouette widths of individual and group members. We selected the final number of clusters based on the highest Average Silhouette Width (ASW) and the balanced distribution of individuals between clusters. This clustering enabled mapping clinical data to analyze symptom prevalence within each subtype.

To assess cluster-specific symptom patterns, we conducted statistical tests of significance. We applied Chi-square tests when the expected cell counts in the contingency tables exceeded 5. We used Fisher’s exact test for cells with lower expected counts, simulated p-values (workspace: 2e8) for symptoms, and simulated Chi-square tests for other clinical variables. Statistical significance was set at p-value < 0.05.

We then calculated symptom frequencies in both absolute counts and relative percentages for each cluster, visualizing these distributions through comparative heatmaps.

More details about the entire framework can be found in S7 Text.

## Results

### Putative causal genes of Long COVID

**Fig 2:**
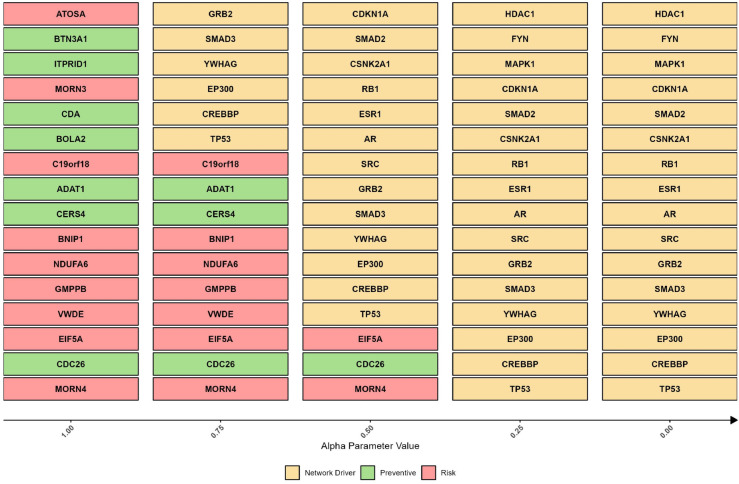
Top putative causal genes ranked by their final score $S_{\text{Causal}}$. These genes, obtained from our framework, are sorted horizontally based on their absolute effect size in ascending order and classified vertically across different *α* values. The parameter *α* balances the direct effect of genes on the disease (${𝒮}_{\text{Risk}}$) and their network controllability roles (${𝒮}_{\text{Network}}$). At α=1, the model outputs disease risk (red) and protective (green) genes. As *α* decreases towards 0, the focus shifts to network driver genes that control the biological system (yellow).

By varying the *α* values in our model, we identified a comprehensive set of putative causal genes for Long COVID, each with distinct roles. Fig 2 shows the sets of these causal genes that correspond to specific values of *α*. As *α* approaches 1, the model outputs genes classified as risk (red) or protective (green), inferred from the color coding of their effect sizes, where red represents positive effect sizes (risk) and green represents negative effect sizes (protective), decreasing *α* towards zero shifts the focus to network driver genes that control the Long COVID PPI network (yellow).

Genes such as membrane occupation and recognition nexus repeat containing 4 (*MORN4*), cell division cycle associated 26 (*CDC26*), and eukaryotic translation initiation factor 5A (*EIF5A*) consistently rank highly across different *α* values (1.00 to 0.50), suggesting a strong causal relationship between their expression levels and disease risk or protective mechanisms. Using SNPs as IVs in our analysis, we estimated the causal effects of gene expression on the Long COVID risk. The consistently high ranking of *MORN4*, *CDC26*, and *EIF5A* suggests that their expression levels can significantly contribute to disease susceptibility, making them potential key targets for intervention strategies aimed at reducing disease risk (see Fig 2). The complete list of SNPs used as IVs for each gene’s expression is provided in S2 Table.

As *α* decreases, the model shifts focus from the MR approach to the CT perspective, prioritizing the balance between risk-related genetic contributions and network control dynamics. This transition highlights the framework’s flexibility in integrating these two viewpoints. Notably, genes such as tumor protein p53 (*TP53*), cyclic adenosine monophosphate response element-binding protein-binding-protein (*CREBBP*), early region 1A binding protein p300 (*EP300*), tyrosine 3-monooxygenase/tryptophan 5-monooxygenase activation protein gamma (*YWHAG*), SMAD family member 3 (*SMAD3*), and the growth factor receptor-bound protein 2 (*GRB2*) become increasingly crucial in the network, emphasizing their roles in maintaining network control (see Fig 2, with these genes highlighted in yellow).

When considering the union of the top genes for each *α* value in our analysis, we identified 32 unique putative causal genes for Long COVID. This comprehensive set of genes represents the most influential factors in the spectrum of our parameter *α*, which balances disease-related impact and network controllability.

Of these 32 genes, 19 have been previously identified in COVID-19 and/or Long COVID studies, reinforcing their importance in the disease process. These include well-known genes such as the androgen receptor (*AR*), butyrophilin subfamily 3 member A1 (*BTN3A1*), cyclin-dependent kinase inhibitor 1A (*CDKN1A*), *CREBBP*, *EIF5A*, *EP300*, estrogen receptor 1 (*ESR1*), atos homolog A (*ATOSA*), FYN proto-oncogene (*FYN*), *GRB2*, histone deacetylase 1 (*HDAC1*), mitogen-activated protein kinase 1 (*MAPK1*), NADH:ubiquinone oxidoreductase subunit A6 (*NDUFA6*), retinoblastoma transcriptional corepressor 1 (*RB1*), SMAD family member 2 (*SMAD2*), *SMAD3*, sarcoma proto-oncogene (*SRC*), *TP53*, and *YWHAG*. These genes have been associated with various aspects of SARS-CoV-2 infection and Long COVID, including roles as hub genes, drug targets, and factors that influence disease severity (Table 1). The high number of confirmed Long COVID genes suggests that our framework effectively identifies putative causal genes.

**Table 1:** Core putative causal genes for Long COVID confirmed by the literature. These 19 genes were validated by existing COVID-19 (COV) and/or Long COVID (LCV) studies, reinforcing our findings. Literature validations include studies on severity (Sev.), regulation (Reg.), and polymorphisms (Polymo.). For more supporting literature, refer to S3 Table.

Gene	Primary Findings	COV	LCV
*AR*	Hub Gene, Drug Target, COVID-19 Severity	[35]	-
*ATOSA*	Downregulated in COVID-19	[36]	-
*BTN3A1*	Predictive Marker	[37]	-
*CDKN1A*	Key Regulator, Drug Target	[38]	[39]
*CREBBP*	Hub/Drug Target	[40]	[41]
*EIF5A*	Drug Target	[42]	-
*EP300*	Hub/Drug/Vaccine Target, COVID-19 Severity, Epigenetic Regulator	[43]	[44]
*ESR1*	Hub/Drug Target, Herpes Zoster Association	[45]	-
*FYN*	Hub/Drug Target	[46]	
*GRB2*	Drug Target	[47]	-
*HDAC1*	Drug Target, Epigenetic Regulation	[48]	[49]
*MAPK1*	Hub/Drug Target	[50]	
*NDUFA6*	Drug Target	[51]	-
*RB1*	Hub Gene, SARS-CoV-2 Oncogenesis, Genetic Polymorphism	[52]	[53]
*SMAD2*	Hub/Drug Target	[54]	[54]
*SMAD3*	Drug Target, Virus-host Interaction	[55]	-
*SRC*	Drug Target, Virus-host Interaction	[56]	[57]
*TP53*	Hub/Drug/Vaccine Target, Critical Gene	[58]	[59]
*YWHAG*	Hub/Vaccine Target, COVID-19 Neurotropism	[60]	-

The remaining 13 genes in our putative causal set represent novel discoveries for COVID-19 and Long COVID research: adenosine deaminase tRNA-specific 1 (*ADAT1*), B-cell lymphoma 2 interacting protein 1 (*BNIP1*), bole-like 2 (*BOLA2*), chromosome 19 open reading frame 18 (*C19orf18*), inositol 1,4,5-trisphosphate receptor interacting domain containing 1 (*ITPRID1*), *CDC26*, cytidine deaminase (*CDA*), ceramide synthase 4 (*CERS4*), casein kinase-2 *α*-1 (*CSNK2A1*), GDP-mannose pyrophosphorylase B synthase (*GMPPB*), MORN repeat containing 3 (*MORN3*), *MORN4*, and the von Willebrand factor D and EGF domains gene (*VWDE*). These previously unlinked genes demonstrate the potential of our framework to reveal novel intervention targets.

**Fig 3:**
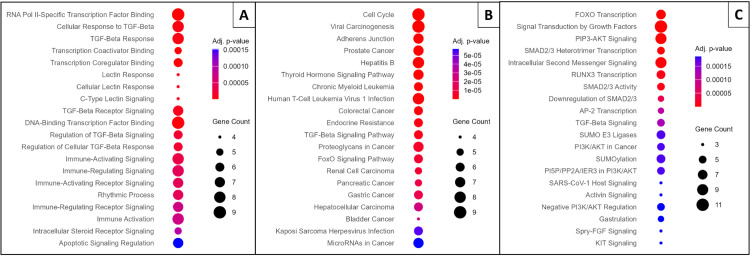
Enrichment analysis (EA) results for the identified Long COVID putative causal genes. (A) Gene Ontology (GO) EA, showing the top 20 enriched terms across Biological Process (BP) and Molecular Function (MF) categories. (B) KEGG pathway EA, displaying the top 20 enriched pathways. (C) Reactome pathway EA, illustrating the top 20 enriched pathways. For all plots, genes are ranked by the lowest adjusted p-value. The y-axis represents the enriched terms or pathways, the size of each dot reflects the number of associated genes, and the color gradient indicates the adjusted p-value, with blue denoting greater significance.

EA of these 32 putative causal genes identified 458 significant pathways in GO (Gene Ontology) [31], 99 in KEGG (Kyoto Encyclopedia of Genes and Genomes) [32], and 246 in Reactome [61]. The top 20 pathways in each database, ranked by adjusted p-value, are shown in Fig 3, with the complete list available in the S4 Table.

Key findings include the transforming growth factor (TGF)-*β* signaling pathway, highlighted in GO and KEGG analyses, which plays a crucial role in immune regulation and tissue repair. Its disruption may contribute to persistent inflammation and fibrosis, leading to lung and organ damage, as observed in Long COVID patients [38]. Similarly, KEGG pathways, such as the cell cycle and viral carcinogenesis, suggest long-term cellular effects of SARS-CoV-2 infection, including abnormal proliferation and senescence, which potentially explain prolonged recovery and tissue dysfunction [53].

GO analysis highlights the importance of immune signaling pathways in ongoing inflammation and autoimmune-like symptoms [62]. Reactome analysis emphasizes Forkhead box O (FOXO) transcription and phosphatidylinositol 3-kinase (PI3K)/protease B (AKT) signaling, which are involved in metabolism, stress responses, cell survival, and growth factor signaling pathways that can affect tissue repair and regeneration [38,41].

These findings reveal potential mechanisms underlying Long COVID and suggest therapeutic targets, such as TGF-*β* signaling and FOXO transcription, to mitigate long-term effects.

#### Integration with COVID-19 polygenic risk scores.

To assess the translational potential and genetic overlap between Long COVID and acute COVID-19 susceptibility, we compared our 32 putative causal genes with existing COVID-19 polygenic risk score (PRS) datasets. This analysis aimed to determine whether the Long COVID genes could be incorporated into current genetic risk prediction models or represent distinct pathophysiological mechanisms.

TSS-based mapping identified 3,190 unique genes from the combined PGS datasets. Direct comparison with our 32 Long COVID genes revealed minimal overlap, with only three genes (9.4%) showing concordance: *ITPRID1*, *GRB2*, and *CDA* (Fisher’s exact test, p = 0.72).

The three overlapping genes demonstrate biological plausibility for the pathogenesis of Long COVID. *ITPRID1* contains domains that interact with IP3 receptors, which are critical for calcium signaling pathways essential for immune cell activation and viral responses [63,64]. *GRB2* has been identified as a potential drug target in COVID-19 due to its role in inflammatory signaling pathways [47]. *CDA*, involved in nucleotide metabolism and immune cell function, has been associated with therapeutic responses in inflammatory conditions [65].

Distance analysis revealed that 22% (7/32) of our Long COVID genes were within 50 kb of COVID-19 PGS variants, and 34% (11/32) within 100 kb, indicating that 66% of our identified genes operate beyond the typical cis-regulatory range captured by current PGS models. The limited overlap indicates that 90.6% of our genes represent distinct genetic mechanisms not currently captured by the COVID-19 susceptibility PGS, suggesting that Long COVID involves fundamentally different genetic architectures than those associated with acute COVID-19 risk. The complete results are provided in the S6 Text.

#### Shared genetic basis of Long COVID and related conditions.

We examine the involvement of the 32 putative causal genes identified for Long COVID in other pathophysiological conditions (Table 2, complete data set in S1 Table). This analysis revealed several distinct patterns of disease overlap, curated from multiple disease databases, including The Human Disease Database (MalaCards), Disease-Gene Associations (DISEASES), The Gene-Disease Network (DisGeNET), Medical Genetics Database (MedGen), and the Gene Curation Coalition (GenCC) (see the Methods section for more information about these databases). Many of these genes are implicated in a spectrum of syndromic, metabolic, autoimmune, connective tissue, and neurodevelopmental disorders that share clinical or biological features with Long COVID manifestations [8,66,67].

**Table 2:** Putative causal genes in Long COVID and their overlap with other pathophysiological conditions. Analysis reveals the involvement of these genes in related diseases, suggesting shared mechanistic pathways underlying Long COVID manifestations.

Gene	Pathophysiological Conditions	Long-COVID Overlap	Databases^*^
*ADAT1*	Developmental syndromes	Neurological and systemic involvement; persistent fatigue and cognitive dysfunction [68]	MC; D
*AR*	ID, metabolic and endocrine disorders	Immune dysregulation and sustained inflammatory responses [68]	MC; D
*BTN3A1*	AD, neurologic and chronic pulmonary conditions	Persistent inflammation and tissue-specific immune dysregulation	MC; D
*CDA*	ID, hematologic, CTD	Chronic immune activation, endothelial dysfunction	DG; MG; MC; D
*CDKN1A*	Metabolic, AD, dev. and tumor-predisposition syndromes	Prolonged inflammatory states [8]	MC; D
*CERS4*	MetS, CV disease, Turner syndrome	Metabolic and vascular complications [67,68]	DG; MC; D
*CREBBP*	Dev/epigenetic syndromes with immune involvement	Epigenetic dysregulation, persistent inflammation [8]	MC; D; MG; DG; GC
*CSNK2A1*	ID, inflammatory syndromes	Extended immune hyperactivity [8]	MC; D; MG; DG; GC
*EIF5A*	MetS, ATD, vascular disease	Chronic inflammation, endothelial dysfunction	MC; D; MG; DG; GC
*EP300*	Dev/epigenetic syndromes, AD disorders	Epigenetic and immune dysregulation	MC; D; MG; DG; GC
*FYN*	AD, vascular, inflammatory conditions	Immune hyperactivity, vascular lesions [67]	MC; D
*GMPPB*	Metabolic, CMS, glycosylation defects	Energy metabolism defects, chronic inflammation [67]	DG; MC; GC
*GRB2*	Chronic myeloproliferative, ID, MetS	Sustained cytokine dysregulation	DG; MC; D
*HDAC1*	ID, metabolic, inflammatory syndromes	Persistent immune activation	DG; MC
*MAPK1*	AD, CV, neurodevelopmental disorders	Prolonged inflammation, CV risk [67]	MG; DG; MC; GC
*NDUFA6*	Mitochondrial dysfunction, vascular disease	Energetic deficits, POTS-like symptoms [66,68]	MC; D
*RB1*	Tumor predisposition, ID features	Immune dysregulation, systemic impairment	MC
*SMAD2*	AD (IBD), CTD, vascular disease	Tissue fragility, chronic inflammation	MC; D; MG; DG; GC
*SMAD3*	AD, CTD, multi-system inflammation	Endothelial, skeletal, immune pathways	MC; D; MG; DG; GC
*SRC*	AD, ID, vascular, inflammatory syndromes	Chronic vascular and immune abnormalities	MC
*TP53*	Tumor predisposition, ID, metabolic disorders	Systemic instability, immune compromise [8]	MC; D; MG; DG; GC
*YWHAG*	Neurodevelopmental, CV, COPD	Respiratory and neurological symptoms [66,68]	DG; MC; D

**Abbreviations:** ^*^Databases: MC: MalaCards; D: DISEASES; DG: DISGENET; MG: MedGen; GC: GenCC. CDL: Cornelia de Lange; ID: Immunodeficiency; AD: Autoimmune Disease; CTD: Connective Tissue Disorder; MetS: Metabolic Syndrome; CV: Cardiovascular diseases; ATD: Autoimmune Thyroid Disease; CMS: Congenital Myasthenic Syndrome; POTS: Postural Orthostatic Tachycardia Syndrome; IBD: Inflammatory Bowel Disease; COPD: Chronic Obstructive Pulmonary Disease. Long-COVID overlap descriptions are supported by representative studies [8,66–68]; identical patterns share the same citation.

A subset of these genes (*CDKN1A*, *CREBBP*, *CSNK2A1*, and *TP53*) is involved in tumor-predisposition syndromes and complex developmental disorders with autoimmune and inflammatory components. Aberrant cytokine signaling and dysregulated immune checkpoints of these conditions suggest potential mechanisms for the prolonged inflammatory responses observed in Long COVID [8]. Similarly, genes such as *C19orf18*, *CDC26*, *MORN3*, *NDUFA6*, *VWDE*, and *YWHAG* are linked to systemic conditions that affect multiple organ systems. Their association with mitochondrial dysfunction and vascular pathologies parallels fatigue, dysautonomia, and endothelial dysfunction, which are commonly reported in Long COVID [66].

*ATOSA* and *GMPPB* are linked to chronic inflammation, mirroring the mechanisms of immune activation and tissue damage implicated in Long COVID. Additionally, *CERS4*, *ESR1*, *FYN*, and *MAPK1* highlight the interplay between immune dysfunction and metabolic disruption, shedding light on the metabolic dysregulation seen in some patients [67].

Our database integration analysis reveals meaningful biological connections between Long COVID and other disorders, particularly immune-mediated conditions and metabolic diseases. The identified genetic overlaps suggest that variants in these genes may influence individual susceptibility to persistent post-viral symptoms, as they do in other chronic conditions. These shared molecular features help explain the diverse manifestations observed in patients with Long COVID [68].

#### Genetic risk and protective factors in Long COVID susceptibility.

Among the 32 putative causal genes obtained from our framework, we identified 16 significant protein-coding genes directly associated with the risk and protection of Long COVID. These genes are involved in critical biological processes such as cell cycle regulation (*CDC26*), apoptosis (*BNIP1*), and immune response (*BTN3A1*) (Table 3).

**Table 3:** Risk and protective putative causal genes for Long COVID ordered by the $S_{\text{Causal}}$ score. Genes are classified as risk or protective factors for Long COVID based on their effect size sign (positive or negative, respectively) when α=1.

Rank	Gene	Description	Effect	Score
1	*MORN4*	MORN Repeat Containing 4	Risk	1.000
2	*CDC26*	Cell Division Cycle 26	Protective	0.878
3	*EIF5A*	Eukaryotic Translation Initiation Factor 5A	Risk	0.614
4	*VWDE*	Von Willebrand Factor D And EGF Domain	Risk	0.453
5	*GMPPB*	GDP-Mannose Pyrophosphorylase B	Risk	0.388
6	*NDUFA6*	NADH Dehydrogenase Subunit A6	Risk	0.286
7	*BNIP1*	BCL2 Interacting Protein 1	Risk	0.263
8	*CERS4*	Ceramide Synthase 4	Protective	0.228
9	*ADAT1*	Adenosine Deaminase Acting on tRNA 1	Protective	0.225
10	*C19orf18*	Chromosome 19 Open Reading Frame 18	Risk	0.225
11	*BOLA2*	BolA Family Member 2	Protective	0.179
12	*CDA*	Cytidine Deaminase	Protective	0.177
13	*MORN3*	MORN Repeat Containing 3	Risk	0.143
14	*ITPRID1*	ITPR Interacting Domain Containing 1	Protective	0.142
15	*BTN3A1*	Butyrophilin Subfamily 3 Member A1	Protective	0.141
16	*ATOSA*	Atos Homolog A	Risk	0.064

**Fig 4:**
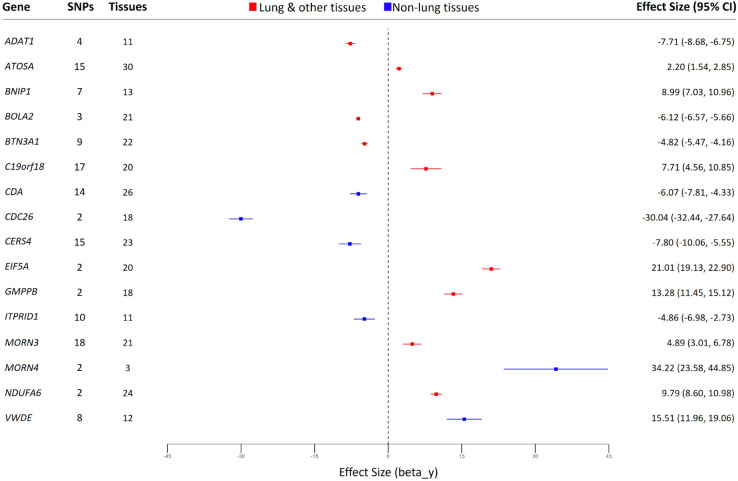
Effect size of the risk and protective putative causal genes for Long COVID. Forest plot shows the significant genes identified at α=1.0, with all causal relationships meeting statistical significance (p-value and FDR < 0.05). Higher expression is associated with increased (positive effect size) or decreased (negative effect size) risk. SNPs: number of associated SNPs; Tissues: number of tissues where the SNPs influence the gene expression. Points show fixed effect size (standardized beta coefficient) with 95% CI error bars. Red bars: lung and other tissues; Blue bars: non-lung tissues. **Abbreviations:** GWAS: Genome-Wide Association Study. SNP: Single Nucleotide Polymorphism. FDR: False Discovery Rate. CI: Confidence Interval.

The forest plot (Fig 4) reveals a wide range of effect sizes for 16 protein-coding genes, from -30.04 for *CDC26* to 34.22 for *MORN4*, indicating varying degrees of influence on Long COVID susceptibility. Through our framework, we identified statistically significant causal relationships for these genes (p-value and FDR < 0.05), with confidence intervals that do not cross zero, providing strong evidence for their potential roles. In particular, genes such as *MORN4*, *CDC26*, *EIF5A* and *VWDE* exhibit the strongest causal associations, with the largest absolute effect sizes.

In our framework, we used varying numbers of SNPs as IVs for each gene’s expression, ranging from 2 SNPs for genes like *MORN4*, *CDC26*, *EIF5A*, *GMPPB*, and *NDUFA6*, to 18 SNPs for *MORN3*. These IVs strengthen the validity of our causal estimates of the relationship between gene expression and the Long COVID risk. The number of tissues in which gene expression was evaluated also varied by gene, enhancing the robustness of our findings in different biological contexts. For instance, *MORN4* showed expression changes in two tissues/cells (left ventricle and thyroid) and in cultured fibroblasts. In contrast, *CDA* demonstrated widespread effects, modulating gene expression across 26 distinct tissues. These included multiple organ systems: adipose, neural, cardiovascular, endocrine, connective, immune, digestive, reproductive, renal, and hematopoietic tissues. This extensive distribution highlights the systemic impact of SNPs on gene expression regulation.

Moreover, the expression patterns of all 16 risk and protective protein-coding genes identified through our framework suggest a systemic involvement in Long COVID. Ten genes showed expression in both both lung and other tissues, while six genes were expressed exclusively in non-lung tissues. This distribution of expression patterns across other non-lung tissues supports the presence of non-respiratory symptoms observed in Long COVID patients, suggesting the involvement of molecular mechanisms beyond the pulmonary system [69].

Directional effects vary among genes, with some showing positive effect sizes (e.g., *ATOSA*, *BNIP1*, *C19orf18*, *EIF5A*, *GMPPB*, *MORN3*, *MORN4*, *NDUFA6*, and *VWDE*) and others negative effect sizes (e.g., *ADAT1*, *BOLA2*, *BTN3A1*, *CDA*, *CDC26*, *CERS4*, and *ITPRID1*). Genes with positive effect sizes suggest that increased expression in relevant tissues is associated with a higher susceptibility to Long COVID. In contrast, those with negative effect sizes indicate that increased expression may reduce the risk or be protective against Long COVID.

Among these 16 protein-coding genes, the roles of *BTN3A1*, *EIF5A*, and *NDUFA6* were previously identified in the pathogenesis of COVID-19, suggesting a potential link between their expression and the development of Long COVID. [37,42,51] (Table 4).

**Table 4:** Summary of three putative causal genes with established links to COVID-19 and hypothesized effects in Long COVID. Additional related literature and references are available in the S3 Table.

Gene	General Function	Role in COVID-19	Long COVID Impact
*BTN3A1*	T-cell activation and regulation [72]	Part of 5-gene signature; higher expression correlates with more ventilator-free days [37]	Higher expression may reduce risk via improved immune regulation
*EIF5A*	Translation regulation, protein synthesis, virus response, cell differentiation [73]	Promotes PRF, translation termination, and ribosome recycling in SARS-CoV-2 [42]	May contribute to persistent symptoms due to enhanced viral response
*NDUFA6*	NADH dehydrogenase activity, electron transport, energy production [73]	Top ten hub gene, significant mRNA differences [51]	Disruptions may increase risk by impacting cardiovascular health

**Abbreviations:** COVID-19: Coronavirus Disease 2019; PRF: Programmed Ribosomal Frameshifting.

*BTN3A1*, an immune system protein involved in T-cell activation and regulation, is part of a 5-gene signature that predicts ventilator-free days in patients with COVID-19 [37]. Our analysis revealed a negative effect size value for *BTN3A1*, suggesting that higher expression is causally associated with better clinical outcomes and a potentially reduced risk of Long COVID. This protective effect may be attributed to its role in promoting a more controlled immune response, thereby reducing long-term complications [70].

In contrast, *EIF5A*, a translation factor that promotes programmed ribosomal frameshifting (PRF), translation termination, and ribosome recycling in SARS-CoV-2 infection, showed a positive effect size value. This function suggests that *EIF5A* may contribute to persistent symptoms in Long COVID by causing ongoing disruptions in translation regulation and protein synthesis, leading to continued immune activation and cellular stress [42].

*NDUFA6*, a key component of the mitochondrial respiratory chain, has been identified among the main genes associated with SARS-CoV-2 infection [51], showing significant differences in gene expression in affected patients. Our findings suggest that changes in *NDUFA6* can negatively impact cardiovascular health and increase the risk of Long COVID. These effects are likely attributable to the critical role of the gene in cellular energy production and mitochondrial function. *NDUFA6*-affected activity can lead to reduced ATP synthesis, increased oxidative stress, and the development of cardiovascular symptoms that are frequently observed in patients with Long COVID [71].

These findings suggest that *BTN3A1*, *EIF5A*, and *NDUFA6* play a significant role in the pathogenesis of COVID-19 with implications for the development of Long COVID. *BTN3A1* appears to confer protective effects through controlled immune responses, potentially reducing the risk of Long COVID. In contrast, *EIF5A* and *NDUFA6* can contribute to persistent symptoms by disrupting translation regulation and impaired mitochondrial function, respectively.

In addition to the three previously mentioned genes, our framework identified 13 novel risk and protective putative causal genes for Long COVID. Among these genes, *CDA*, *ADAT1*, *CERS4*, *CDC26* and *BOLA2* were enriched mainly in our analyzes with significant roles in crucial pathways, including nucleotide metabolism, RNA editing, lipid metabolism, cell cycle regulation, and iron-sulfur cluster assembly [5,74–80].

*CDA* and *ADAT1* are both involved in nucleotide metabolism and RNA editing processes. *CDA* is crucial for the salvage of pyrimidine and the balance of the nucleotide pool, potentially affecting the integrity of the RNA and the function of the immune system [74]. Similarly, *ADAT1* is involved in pre-mRNA editing, converting adenosine to inosine in eukaryotic tRNA, potentially influencing inflammatory responses [75]. Their roles as risk factors can be hypothesized based on their participation in these critical cellular processes, which could contribute to the persistent symptoms observed in patients with Long COVID [5].

*CERS4* and *BOLA2* are involved in cellular metabolism and homeostasis. *CERS4* facilitates sphingosine N-acyltransferase activity and is implicated in ceramide synthesis, influencing lipid metabolism and cellular signaling pathways [76]. *BOLA2* works in iron maturation and is part of the iron-sulfur cluster assembly complex, playing a role in cell redox homeostasis [77]. The association of risk with these genes might be related to their impact on various cellular processes, including signaling pathways and cellular respiration. Its role may be associated with the various symptoms observed in Long COVID cases [78].

*CDC26* is part of the anaphase-promoting complex (APC) involved in cell cycle regulation [79]. Its role as a risk factor can be attributed to its function as a ubiquitin-protein ligase, which manages the proteolysis of cell cycle proteins. This could alter cellular repair and regeneration processes, possibly explaining the prolonged cellular damage observed in individuals with Long COVID [80].

The detailed results of the pathway EA using the GO, KEGG and Reactome databases, including significantly enriched biological processes, molecular functions, cellular components, and pathways, are detailed in S4 Table.

#### Network driver genes that control Long COVID network.

Our multi-omics framework identified 16 putative causal genes that function as network drivers in Long COVID. In CT, such drivers represent critical nodes whose manipulation manages the overall state and dynamics of the system, regulating numerous downstream genes and pathways. By identifying these regulatory hubs, our approach reveals strategic intervention points that could restore normal function or mitigate disease effects, offering therapeutic targets to modify network behavior and clinical outcomes [14].

The identified core network driver genes have at least 150 connections to other nodes, which highlights their significant influence. Disruption of these genes under normal conditions could contribute to the pathogenesis of Long COVID, making them potential therapeutic targets to restore normal function in affected patients (Table 5).

**Table 5:** Network driver genes for Long COVID ordered by the $S_{\text{Causal}}$ score. The *K* column represents the total degree (total interactions), *K*_*in*_ describes the in-degree (incoming interactions), and *K*_*out*_ denotes the out-degree (outgoing interactions).

Rank	Gene	Description	*K*	$K_{in}$	$K_{out}$	Score
1	*TP53*	Tumor Protein p53	299	196	103	1.000
2	*CREBBP*	CREB Binding Protein	273	153	120	0.913
3	*EP300*	E1A Binding Protein p300	270	162	108	0.903
4	*YWHAG*	14-3-3 Protein Gamma	252	180	72	0.843
5	*SMAD3*	SMAD Family Member 3	225	143	82	0.753
6	*GRB2*	Growth Factor Receptor Bound 2	210	96	114	0.702
7	SRC	SRC Proto-Oncogene	195	92	103	0.652
8	*AR*	Androgen Receptor	179	112	67	0.599
9	*ESR1*	Estrogen Receptor 1	174	68	106	0.582
10	*RB1*	Retinoblastoma 1	169	106	63	0.565
11	*CSNK2A1*	Casein Kinase-2 *α*-1	165	89	76	0.552
12	*SMAD2*	SMAD Family Member 2	161	99	62	0.538
13	*CDKN1A*	Cyclin-Dependent Kinase Inhibitor 1*α*	158	108	50	0.528
14	*MAPK1*	Mitogen-Activated Protein Kinase 1	157	80	77	0.525
15	*FYN*	FYN Proto-Oncogene	153	63	90	0.512
16	*HDAC1*	Histone Deacetylase 1	151	95	56	0.505

**Abbreviations:** *K*: total degree (all interactions); *K*_*in*_: in-degree (incoming interactions); *K*_*out*_: out-degree (outgoing interactions).

Of the 16 identified network driver genes, 14 were associated with pathways enriched for COVID-19, Long COVID, or both. These pathways involve essential cellular functions, including cell proliferation, differentiation, cell cycle progression, DNA repair, inflammation, and immune responses. Disruptions in these processes can lead to persistent symptoms of Long COVID, chronic inflammation, neurodegeneration, and immune dysfunction (Table 6).

**Table 6:** Long COVID roles of the identified network driver genes. Key protein functions and enriched pathways obtained from GO, KEGG, or Reactome, along with their roles in COVID-19 and Long COVID pathogenesis. All pathway enrichments meet statistical significance thresholds (p-value and FDR < 0.05).

Gene	Function	Paths	Main Path	Roles	Ref.
*AR*	Steroid-hormone transcription factor, regulates cell proliferation	88	Regulation of miRNA transcription	Affects TMPRSS2 and ACE2 expression, linked to persistent symptoms in males	[81]
*CDKN1A*	Inhibits CDKs, regulates cell cycle	152	p53 signaling pathway	Involved in SARS-CoV-2 entry, tissue damage, fibrosis	[82]
*CREBBP*	Acetyltransferase, regulates gene expression	118	Histone acetyltransferase activity	Controls inflammation, may trigger neurodegeneration	[41]
*EP300*	Acetyltransferase, regulates cell growth	184	Histone acetyltransferase activity	Regulates ACE2, critical in inflammation, persistent immune responses	[44]
*ESR1*	Estrogen receptor, regulates transcription	113	Intracellular estrogen receptor signaling pathway	Protective against COVID-19, reduces inflammation, immune dysfunction in women	[83]
*FYN*	Non-receptor kinase, regulates immune response	115	Immune response-regulating signaling pathway	Regulates inflammation, may be linked to immune dysregulation	[62]
*HDAC1*	Histone deacetylase, modulates gene expression	98	Regulation of apoptotic signaling pathway	Modulates inflammation and apoptosis in COVID-19	[84]
*MAPK1*	Kinase involved in signal transduction	246	Immune response-activating signaling pathway	Controls inflammation and cytokine responses in COVID-19	[85]
*RB1*	Tumor suppressor, regulates cell cycle	25	Regulation of apoptotic signaling pathway	May interact with viral mechanisms, potential oncogenic effects	[53]
*SMAD2*	Mediates TGF-*β* signals, regulates cell growth	114	TGF-*β* receptor signaling pathway	Involved in fibrosis and other complications post-COVID	[82]
*SMAD3*	Mediates TGF-*β* signals, regulates cell differentiation	168	miRNA transcription	Linked to pulmonary fibrosis, impacts post-COVID severity	[82]
*SRC*	Non-receptor kinase, regulates gene transcription	257	Immune response-regulating signaling pathway	Mediates viral entry, chronic inflammation, immune dysregulation	[57]
*TP53*	Tumor suppressor, regulates apoptosis and DNA repair	263	Intrinsic apoptotic signaling pathway (DNA damage response)	Influences cytokine release, immune response in COVID-19	[86]
*YWHAG*	Adapter protein in signaling pathways	26	PI3K-Akt signaling pathway	Involved in cell survival, inflammation, and immune responses in COVID-19	[87]

**Abbreviations:** GO: Gene Ontology; KEGG: Kyoto Encyclopedia of Genes and Genomes; Reactome: Reactome Pathway Database; FDR: False Discovery Rate.

The extensive findings of our functional enrichment studies on these putative causal genes of network drivers, obtained from GO, KEGG, and Reactome, are presented in detail in the S4 Table.

**Fig 5:**
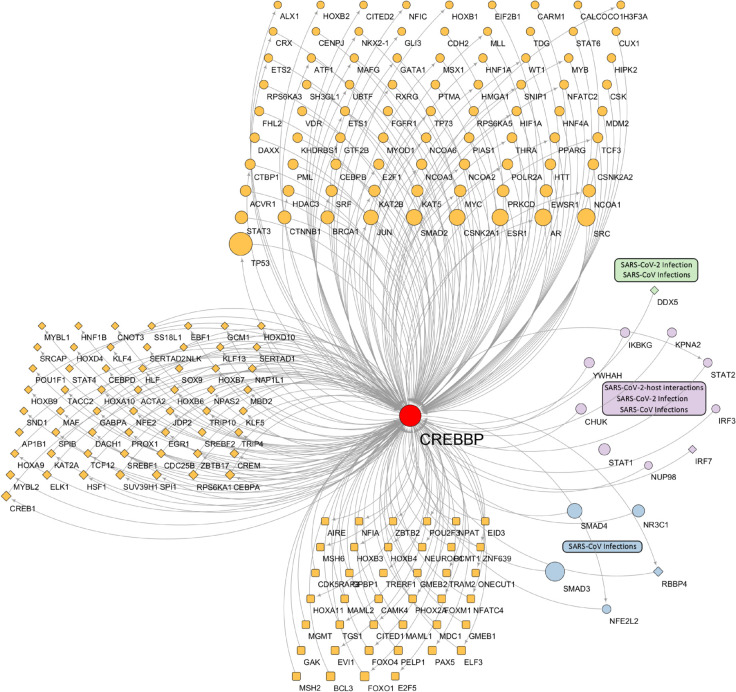
Network plot highlighting a network driver gene for Long COVID. Our analysis identified *CREBBP* as a key network driver gene for Long COVID, supported by existing literature, with 273 total interactions (153 incoming, 120 outgoing). Connected genes are represented by three shapes based on network control properties: ellipses for critical genes (removal increases the required driver nodes), diamonds for ordinary genes (removal maintains the driver nodes), and round rectangles for redundant genes (removal preserves the control). The three most enriched pathways are shown in green, purple, and blue, with node sizes proportional to their K-degree (network connectivity).

In Fig 5, we provide a detailed example of *CREBBP*, one of the genes identified by our framework and confirmed in the literature. This gene was chosen because of its larger number of connections compared to other genes, highlighting its essential role in the network. The plots of the other identified network driver protein-coding genes for Long COVID are provided in S1 Fig.

### Gene expression clustering reveals Long COVID subtypes

We clustered Long COVID patients into subgroups using gene expression data from the 32 putative causal genes identified in our framework. Moreover, we hypothesized that distinct gene expression patterns of risk and protective genes, as well as network driver genes, underlie different clinical characteristics in patients. Using Consensus Clustering (ConC) [34], we identified subgroups of patients who demonstrated coherent clustering and balanced distributions (i.e., not skewed toward a single subset).

The analysis identified three distinct Long COVID subtypes, aligning with the findings of the three groups reported in previous research [5,66], each with high ASW values indicating robust clustering: Cluster 1 included 65 individuals (ASW: 0.93), Cluster 2 contained 53 individuals (ASW: 0.85), and Cluster 3 consisted of 36 individuals (ASW: 0.75).

**Fig 6:**
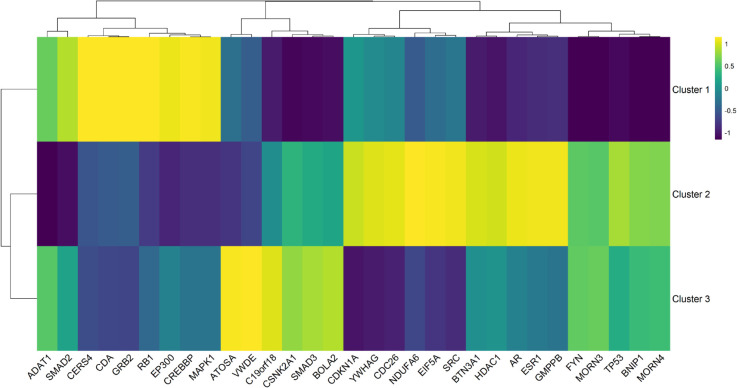
Cluster-level heat-map of the 32 candidate Long-COVID genes. The heat-map shows the mean gene expression for each cluster, highlighting distinct expression patterns across the three patient groups. Hierarchical clustering of genes (shown at top) reveals coordinated expression patterns. The color gradient represents *z*-scored log_2_ expression values (viridis color scale: dark-purple = low expression, bright-yellow = high expression), demonstrating cluster-specific gene signatures associated with different Long COVID phenotypes. A sample-level heat-map showing individual subject gene expression is available in S5 Table and S2 Fig.

Table 7 presents the comprehensive distribution of symptom frequencies in the three clusters, providing context for the clinical heterogeneity observed in our cohort. We performed a direct differential expression analysis as shown in Fig 6. These putative causal genes exhibited distinct expression patterns across all subtypes, highlighting their potential role in distinguishing symptom profiles. To explore this further, we map symptom prevalence within groups to evaluate whether gene expression patterns align reliably with the identified symptoms. Significant differences in symptom distributions (p-value < 0.05) were observed, with symptoms grouped into broader categories, including respiratory, gastrointestinal, neurological, metabolic, psychological, dental, and sleep-related problems, allowing for a comprehensive comparison between groups. Table 8 complements this analysis by summarizing the key genes identified per cluster, including differentially expressed genes, their regulatory patterns, biological functions, and associated enriched pathways that contribute to the distinct manifestations of Long COVID symptoms [21]. Details of the RNA-seq and clinical datasets used in this analysis are provided in the Methods section.

**Table 7:** Cluster-specific symptom prevalence in Long COVID patients. This table highlights the most characteristic symptoms for each cluster, showing count and percentage (in parentheses) of patients experiencing each symptom. Symptoms are listed in order of prevalence within each cluster to emphasize the cluster-defining characteristics. Complete clinical data and statistical comparisons are available in S5 Table and S2 Fig.

C1: Respiratory-Sleep	Count (%)	Key Characteristics
Weakness or Fatigue	40 (52.6)	Predominant fatigue
Memory/Thought Problems	39 (51.3)	Cognitive symptoms
Sleep Problems	37 (48.7)	Sleep disturbances
Eating More/Less	34 (44.7)	Appetite changes
Muscle Pain	33 (43.4)	Physical discomfort
Shortness of Breath	26 (34.2)	Respiratory issues
Chest Pain/Cardiac Issues	22 (28.9)	Cardiac symptoms
Increased Mucus	21 (27.6)	Respiratory secretions
Lung Problems	16 (21.1)	Pulmonary complications
**C2: Neuropsychological-Dental**	**Count (%)**	**Key Characteristics**
Eating More/Less	34 (43.6)	Appetite dysregulation
Weakness or Fatigue	33 (42.3)	General fatigue
Memory/Thought Problems	30 (38.5)	Cognitive impairment
Muscle Pain	29 (37.2)	Musculoskeletal pain
Shortness of Breath	24 (30.8)	Respiratory symptoms
Sleep Problems	22 (28.2)	Sleep disorders
Anxiety/Depression	17 (21.8)	Psychological symptoms
Chest Pain/Cardiac Issues	16 (20.5)	Cardiac manifestations
Increased Mucus	12 (15.4)	Respiratory secretions
Cavities/Teeth Problems	11 (14.1)	Dental complications
**C3: Gastrointestinal-Metabolic**	**Count (%)**	**Key Characteristics**
Eating More/Less	17 (47.2)	Metabolic dysregulation
Weakness or Fatigue	15 (41.7)	General fatigue
Nausea/Diarrhea/Vomiting	14 (38.9)	GI disturbances
Memory/Thought Problems	12 (33.3)	Cognitive symptoms
Muscle Pain	12 (33.3)	Physical discomfort
Sleep Problems	12 (33.3)	Sleep disturbances
Headaches	12 (33.3)	Neurological symptoms
Anxiety/Depression	9 (25.0)	Psychological symptoms
Shortness of Breath	9 (25.0)	Respiratory symptoms
Chest Pain/Cardiac Issues	8 (22.2)	Cardiac symptoms

**Abbreviation:** C: Cluster. GI: Gastrointestinal.

**Table 8:** Gene expression patterns, pathways, and symptoms across Long COVID clusters. Cluster-specific genes highlight functions and enriched pathways associated with symptom persistence. This table shows relationships between clusters, symptoms, and pathways with significant biological relevance (p-values and FDR < 0.05).

Clus.	Symptoms	Gene	Reg.	Function	Enriched Pathway
1	Respiratory issues, Sleep disturbances	*CREBBP*	Up	Transcriptional coactivator, hypoxia response	HIF-1 signaling: mediates cellular response to hypoxia
		*GRB2*	Up	Growth factor signaling mediator	ErbB signaling: regulates cell survival and stress response
		*MAPK1*	Up	Stress-responsive kinase	MAPK signaling: controls cellular response to stress and inflammation
		*SMAD2*	Up	Signal transducer in TGF-*β* pathway, regulates inflammation	TGF-*β* signaling: controls inflammatory response and tissue repair
2	Psychological symptoms, Dental issues	*CDC26*	Up	Cell cycle regulator	Controls cellular homeostasis
		*CDKN1A*	Up	Cell cycle regulator, Stress response	p53 signaling: mediates cellular stress response
		*ESR1*	Up	Nuclear receptor, inflammation control	Nuclear receptor signaling: regulates inflammatory responses
		*YWHAG*	Up	Signal transduction regulator	PI3K-Akt signaling: controls cell survival and stress adaptation
3	Gastrointestinal symptoms, Metabolic disturbances	*HDAC1*	Down	Epigenetic regulator	Chromatin modification: regulates gene expression
		*NDUFA6*	Up	Mitochondrial function	Oxidative phosphorylation: controls energy metabolism
		*SRC*	Down	Tyrosine kinase, immune regulation	Immune response signaling: controls inflammation
		*TP53*	Down	Stress response regulator	Apoptotic signaling: regulates cell death and survival

**Abbreviations:** Clus.: Cluster; Reg.: Regulation; FDR: False Discovery Rate.

Cluster 1 showed a symptom profile dominated by respiratory problems and sleep disturbances. Increased mucus was reported by 29.23% of patients in this cluster, significantly higher than in cluster 2 (15.09%) and cluster 3 (16.67%) ($\chi^{2}$ *p-value* $=1.07\times 10^{-41}$, *adjusted p-value* $=1.18\times 10^{-40}$). Lung (23.08%) and smell and/or taste problems (20.00%) were similarly more prevalent in cluster 1 ($\chi^{2}$ *p-value* $=7.69\times 10^{-7}$, *adjusted p-value* $=1.21\times 10^{-6}$; $\chi^{2}$ *p-value* $=8.23\times 10^{-15}$, *adjusted p-value* $=2.59\times 10^{-14}$, respectively). Sleep problems were also more common in cluster 1 (49.23%) compared to cluster 2 (28.30%) and cluster 3 (33.33%) ($\chi^{2}$ *p-value* $=9.98\times 10^{-33}$, *adjusted p-value* $=5.49\times 10^{-32}$), in agreement with previous reports indicating sleep disturbances as key features of specific Long COVID phenotypes [88]. This pattern is consistent with multiple cluster analyses that identify distinct respiratory and fatigue-related symptom groups [5,66]. The corresponding gene expression profile revealed elevated expression of *CREBBP*, *GRB2*, *MAPK1*, and *SMAD2*, which are involved in inflammatory responses, stress adaptation, and TGF-*β* signaling pathways associated with respiratory function and sleep regulation. These molecular findings suggest that the genes selected in cluster 1 effectively captured the biological mechanisms underlying the respiratory and sleep-related symptoms of this group.

A higher prevalence of psychological symptoms and dental problems characterized the second group. Anxiety and depression were observed in 37.74% of the patients, slightly higher than in cluster 1 (36.92%) and significantly higher than in cluster 3 (25.00%) ($\chi^{2}$ *p-value*  = 0.0082, *adjusted p-value*  = 0.0106). Cavities and tooth problems affected 18.87% of the patients in cluster 2, compared to 13.85% in cluster 1 and 5.56% in cluster 3 ($\chi^{2}$ *p-value* $=2.32\times 10^{-9}$, *adjusted p-value*  = 4.65 × 10^−9^). The gene expression analysis in cluster 2 revealed upregulation of *CDC26*, *CDKN1A*, *ESR1*, and *YWHAG*, genes associated with cell cycle regulation, stress response, and inflammation control, respectively. In particular, *ESR1* has been implicated in psychiatric disorders, and *YWHAG* is known to modulate multiple signaling pathways relevant to mood regulation [89]. The prominence of neuropsychological symptoms in this cluster aligns with other Long COVID clustering studies that have identified distinct neurocognitive and mood-related phenotypes [5,66]. Furthermore, recent studies suggest an interplay between COVID-19 and the deterioration of oral health, providing a rationale for the increased dental problems in cluster 2 [90]. These findings reflect the biological mechanisms behind the psychological and dental symptoms of this group.

Cluster 3 was defined by gastrointestinal symptoms (GI) and metabolic disturbances. A significant 38.89% of patients in this cluster experienced nausea, diarrhea, and/or vomiting, higher than in cluster 1 (13.85%) and cluster 2 (7.55%) ($\chi^{2}$ *p-value*  = 1.37 × 10^−116^, *adjusted p-value*  = 3.02 × 10^−115^). Eating more or less was reported by 47.22% of patients in cluster 3, comparable to cluster 1 (47.69%) but higher than cluster 2 (37.74%) ($\chi^{2}$ *p-value*  = 1.56 × 10^−13^, *adjusted p-value*  = 4.29 × 10^−13^). Headaches were also more common in cluster 3 (33.33%) compared to cluster 1 (30.77%) and cluster 2 (22.64%) ($\chi^{2}$ *p-value*  = 2.19 × 10^−20^, *adjusted p-value*  = 8.02 × 10^−20^). The gene expression profile showed downregulation of *HDAC1*, *SRC*, and *TP53*, along with upregulation of *NDUFA6*, genes associated with metabolic regulation, immune response, and cellular stress pathways. These alterations are correlated with evidence of persistent metabolic and immune dysregulation in Long COVID [67]. The prominent GI issues are consistent with the recognition of Long COVID clusters focused on GI [5,66], showing the heterogeneous nature of post-COVID symptom profiles. These molecular profiles are correlated with the GI and metabolic symptoms identified in group 3, highlighting the ability of these genes to capture the biological processes driving these manifestations.

Our demographic analysis did not reveal significant differences in key covariates between the three identified symptom clusters. Age, sex, and smoking status were evenly distributed across clusters (all p-values > 0.1), indicating that the observed symptom differentiation was not attributable to these factors. We also conducted comprehensive statistical testing of multiple potential confounding variables using chi-squared tests, including race, pre-existing conditions such as asthma, cancer, diabetes, hypertension, and cardiovascular disorders, with none showing significant differences between clusters (all p-value > 0.05), with the sole exception of ulcerative colitis (p-value = 0.016). This uniform distribution of demographic and clinical characteristics across clusters strengthens the biological validity of our gene expression-based clustering approach, suggesting that the observed symptom patterns reflect genuine molecular distinctions rather than artifacts of population stratification or comorbidity distribution.

Integrating symptom profiles with gene expression clustering demonstrates how our identified genes stratify Long COVID patients into biologically distinct groups, each cluster exhibiting unique symptom signatures. Cluster 1 exhibits predominantly respiratory and sleep disturbances, suggesting potential benefits from therapies targeting these pathways. Cluster 2 features psychological and dental problems, indicating the need for interventions that address stress-related pathways and oral health. Cluster 3 presents GI and metabolic symptoms, suggesting treatments focused on metabolic and digestive support. The alignment between gene functions and symptom distributions validates the biological relevance of these putative causal genes and their roles in initiating diverse clinical manifestations. More details, including complete statistical analyses and p-values, are available in S5 Table.

## Discussion

Long COVID, or PASC, is a multisystemic disorder whose respiratory, neurological, cardiovascular, and gastrointestinal manifestations can persist for months after the acute phase [1,2,4–6]. Despite its growing clinical impact, decisive genetic drivers remain elusive. We address this gap with a multi-omics framework that combines TWMR with CT concepts to prioritize genes that show evidence of expression-mediated effects on disease risk and occupy critical positions within the network for controllability. By design, this framework identifies putative risk genes (strong cis-MR support) and network-driver genes (nodes whose removal increases the number of control inputs), yet allows the boundary to shift as stronger instruments and resources become available or as additional trans-eQTLs are identified. All input paths, LD panels, and parameters, including the balancing factor *α*, are exposed in the public code (https://github.com/SindyPin/Causal-Multiomics-Method) and the Shiny app (https://sindypin.shinyapps.io/github/), which allows users to reproduce or refine every result with a single configuration change.

Our study used publicly available meta-analyzed summary statistics, preventing direct severity-adjusted models that could unravel the Long COVID-specific genetic liability from severe acute disease effects. Although severity adjustment would theoretically provide this distinction, it risks collider bias if both host genetics and viral load independently influence the outcomes [91,92]. The GWAS dataset we used mitigated confounding through case-control designs comparing Long COVID patients with COVID-positive controls [17]. Clinical evidence supports Long COVID as distinct: 10-30% of non-hospitalized and up to 67% of mild-moderate cases develop persistent symptoms, the majority arising from mild rather than severe infections [93,94]. Our complementary approaches (TWMR for causal inference and CT for network regulation) capture Long COVID-specific mechanisms independent of acute severity pathways.

We used the publicly accessible COVID-19 Host Genetics Initiative dataset from Lammi et al. (2025) [17], which included 24 cohorts with broad ancestry diversity—critical given documented Long COVID disparities across populations. Although the larger 23andMe GWAS (54,000 cases, 120,000 controls) [95] offers superior statistical power and stronger MR instruments through population homogeneity and positive SARS-CoV-2 controls, it was not available during our analysis. Furthermore, the 23andMe cohort is predominantly of European ancestry, limiting its generalizability to the development of broadly applicable therapeutic strategies. In contrast, our chosen dataset’s multi-ancestry composition better supports inclusive therapeutic development despite its smaller sample size.

The impact of ancestry on susceptibility to Long COVID, together with our genetic findings, deserves careful consideration for clinical translation. The Long COVID GWAS we analyzed included cases and COVID-19-positive controls from six ancestries (European, East Asian, American mixed, African, South Asian, and others) [17]. While European ancestry predominated, the inclusion of diverse populations allowed the discovery of cross-ancestry variation and highlighted important population-specific genetic differences. For example, risk allele frequencies can vary substantially by ancestry, as demonstrated by variants with frequencies ranging from 1.6% in non-Finnish Europeans to 36% in East Asians, highlighting the critical importance of ancestry-aware interpretation of genetic findings.

Our RNA-seq cohort represents individuals from diverse racial backgrounds, including Black or African American, Asian, White, American Indian/Alaska Native, Native Hawaiian or Other Pacific Islander, and those identifying with multiple races. This diversity strengthens the generalizability of our transcriptomic findings and ensures that ancestry-related biological variability is captured in our integrative framework. However, we acknowledge that the current genetic architecture of Long COVID may not fully capture population-specific susceptibility patterns, and future research should prioritize ancestry-stratified analyses to prevent exacerbating health disparities in the diagnosis and treatment of Long COVID.

The instrumental variants in our analysis were restricted to high-confidence cis-eQTLs $\left(P<5\times 10^{-6},\;r^{2}<0.01;\;F>10\right)$ to reduce the bias of weak instruments. Causal estimates were obtained using Mt-Robin, which reduces pleiotropic outliers through robust regression [11]. Our framework integrates TWMR and CT, as they identify causal genes from complementary perspectives and utilize different data types. TWMR uses GWAS and eQTL data, while CT relies on gene expression data and PPI networks, thus avoiding potential conflicts in data types and model assumptions. These methods operate at different analytical levels: TWMR identifies individual causal genes using genetic instruments in accordance with MR principles, while CT analyzes system-level regulatory effects using network topology assessment. This sequential design ensures that each method operates within its valid assumption framework. The implementation of Mt-Robin incorporates built-in robustness features through its resampling-based approach (10,000 iterations), which accounts for LD and tissue-tissue correlations, while explicitly modeling SNP-specific random effects ($\theta_{i}$) to capture horizontal pleiotropy. This approach maintains type I error control even with up to 50% of instruments invalid. We acknowledge the limitations of using multi-tissue cis-eQTLs and have avoided trans-eQTLs in the current analysis due to their context specificity and low signal-to-noise ratio. However, the framework supports alternative eQTL files, including trans-eQTLs (e.g., eQTLGen), which users can incorporate to test the robustness of gene classifications across MR–CT boundaries.

Given the predominance of participants of European ancestry in GWAS discovery, our use of the GTEx-EUR LD reference aligns with the characteristics of the study population. The framework enables researchers to integrate alternative panels matched by ancestry with a straightforward configuration change before the LD-clumping step, enhancing flexibility for population-specific analyses and reducing potential LD mismatches. Despite the strengths of our approach, we acknowledge certain inherent limitations of the MR methodology, including effects on the population structure, potential unmeasured confounders, long-range LD patterns, and horizontal pleiotropy. Although we have implemented Mt-Robin and leave-one-out diagnostics to mitigate these concerns, the reported effect sizes should be interpreted as approximations of lifelong expression liability rather than definitive causal estimates.

A critical consideration in causal inference studies is whether the tissue sources of eQTL data align with the organ systems most affected by Long COVID. To address this, we deliberately selected cis-eQTL from 49 human tissues, ensuring a broad coverage of the principal organ systems implicated in the syndrome (S1 Text). Respiratory involvement is captured through lung tissue, directly reflecting the pulmonary manifestations commonly reported in Long COVID. Neurological symptoms are comprehensively represented through extensive coverage of brain regions, including the amygdala, anterior cingulate cortex, caudate, cerebellar hemisphere, cerebellum, cortex, frontal cortex, hippocampus, hypothalamus, nucleus accumbens, putamen, substantia nigra, and spinal cord, ensuring that central nervous system dysfunction is accurately depicted. Cardiovascular complications are addressed through various tissues, including the aorta, atrial appendage, coronary arteries, left ventricle, and tibial arteries. At the same time, immune dysregulation—a hallmark of Long COVID—is reflected in whole blood, EBV-transformed lymphocytes, and spleen tissue. This comprehensive tissue coverage increases the biological validity of our causal gene identification strategy, supporting the interpretation that our multi-omics integration captures expression patterns relevant to the multi-organ nature of Long COVID.

Our framework addresses batch effects by integrating data strategically and modularly rather than combining raw data across studies. We utilize summary statistics from GWAS and eQTL studies that incorporate study-specific corrections and quality control measures from the original analyses, ensuring that population structure and technical artifacts are handled within each component’s validated statistical framework. The Mt-Robin method specifically addresses population structure through its resampling procedure (10,000 iterations) and LD-aware analysis, which accounts for genetic correlations and population stratification without requiring additional correction steps. This sequential integration approach, where the GWAS, eQTL, RNA-seq, and PPI data contribute to distinct analytical stages, prevents the propagation of batch effects while maintaining methodological integrity at each step.

This framework highlighted 32 genes whose combined MR and network evidence suggest that immune regulation, viral carcinogenesis, cell-cycle control, and metabolic adaptation contribute to Long-COVID pathophysiology. The list includes *TP53*, *CREBBP*, *EP300*, *SMAD3*, *GRB2*, and *YWHAG*, genes likely responsible for driving persistent inflammation, tissue remodeling, and immune exhaustion—mechanisms consistent with the omnigenic model, in which peripheral regulators collectively influence the core disease genes [96]. Enrichment analyses may inherit annotation bias from curated databases; cross-validation in independent omics layers will therefore be essential.

We designed our framework to be network-agnostic, allowing researchers to explore Long COVID causal genes using their preferred network resources while providing systematic validation across multiple databases. We compared results from our primary network (6,327 genes) [22] with the OmniPath database (4,789 genes) [97], which together share 3,027 overlapping genes. Controllability scores demonstrated substantial consistency, with a Spearman correlation of ρ=0.61(p<2.2 × 10^−16^) for overlapping genes, indicating moderate to strong agreement despite differences in network topologies. This correlation validates the robustness of our approach while acknowledging that different PPI networks capture distinct interaction types. Our framework delivers this diversity by providing pre-processed versions of multiple networks and comparative visualization tools for multi-network validation (S6 Table and S3 Fig).

We use degree centrality solely to classify the selected driver genes, as it serves as a practical proxy for regulatory influence—genes with more downstream connections may affect broader regions of the network. This ranking is performed after identifying control-critical genes and is not used to determine driver status. Although betweenness or eigenvector centrality could offer complementary insights, they are not directly related to the structural controllability framework proposed by Liu et al. (2011) [98], which forms the basis of our approach. Users may adapt the ranking method in the code to incorporate alternative centralities if desired.

Our fusion methodology is designed to combine the complementary strengths of MR and network approaches. The observed switching behavior across *α* values reflects the distinct nature of the two scoring systems: $S_{\text{Risk}}$ captures direct causal evidence through statistical significance thresholds from MR analysis, while $S_{\text{Network}}$ identifies regulatory control points through network topology measures. When we systematically evaluated seven alternative normalization strategies (Min-Max, Box-Cox, Yeo-Johnson, Rank-Inverse Normal, Quantile, Asinh, and Rank-based transformations) across the full range of parameters *α*, all approaches demonstrated similar transitions between gene sets, confirming that this behavior arises from inherent biological differences in what each method captures rather than methodological artifacts.

This complementary design allows researchers to explore the spectrum systematically—from genes with strong statistical causal evidence (*α* → 1) to critical network regulators (*α* → 0), using user-defined top-*K* gene sets and normalization methods. Rather than artificially forcing the smooth mixing of incompatible scoring distributions, our approach preserves the interpretability of each methodology while enabling users to select the balance that aligns with their biological hypotheses and validation strategies. This flexibility represents a strength of the framework, as it acknowledges that causal genes and network drivers may represent distinct but equally important therapeutic target classes in complex diseases, such as Long COVID. The complete results of the normalization analysis are provided in S7 Table and S4 Fig.

The adjustable parameter *α* balances the MR and the network evidence. Setting α=1 privileges genes with strong cis-MR support, making them suitable for hypothesis-driven validation studies; α=0 favors regulators when the goal is to map the intervention points. A sweep of sensitivity (Δα=0.1) shows that the top-ranked list is stable in 0.3≤α≤0.7. We encourage users to perform their own search on the *α* grid depending on their research goals through our available Shiny app (https://sindypin.shinyapps.io/github/).

Our analysis identified three transcriptomic endotypes corresponding to distinct Long COVID profiles: respiratory-sleep, neuro-psychological/dental, and gastro-metabolic disturbances. However, we do not claim that these three subtypes represent the definitive structure of the syndrome. The number of clusters was chosen using internal pre-specified criteria (silhouette, size balance, and resampling stability); clinical symptom patterns were then interpreted *post hoc*. Consequently, the reported cluster-symptom associations should be read as conditional on the selected *K* and interpreted with appropriate caution regarding post-selection inference.

In practical terms, our goal was to stratify patients into clinically useful groups rather than to nominate single-gene biomarkers. Most of the 32 genes are not strongly associated with individual symptoms when tested individually (*AR* is the only exception). However, their joint expression pattern reliably distinguishes patient subgroups, consistent with post-viral biology, in which pathway-level (not single-gene) dysregulation dominates. This justifies our multigene integrative strategy and cautions against over-interpreting one-gene-at-a-time symptom associations.

Generalization remains a field-wide challenge. Currently, there are no publicly accessible whole-blood transcriptomic cohorts with patient-level symptom matrices that would permit a like-for-like external evaluation. We therefore provide all artifacts necessary for future validation—code, centroid coordinates, and a conservative assignment rule with an unclassified option—to facilitate prospective testing as appropriate datasets emerge. Meantime, we prioritize robustness checks that do not rely on outcome re-optimization, such as split-sample agreement and subsampling stability.

Clinically, these endotypes should be viewed as testable hypotheses that can guide trial design and biomarker development rather than as fixed diagnostic categories. They offer a principal method for (i) enriching trials for patients sharing dominant biological programs, (ii) aligning mechanistic studies with coherent patterns of symptoms, and (iii) developing explicit and auditable decision rules. Limitations include potential post-selection bias, cohort-specific effects, and the cross-sectional nature of transcriptomic sampling. Future work should include preregistered analysis plans for selection *K*, longitudinal stability of assignments, and multi-cohort, multi-tissue evaluations once compatible resources become available.

The 32 highlighted genes overlap disorders characterized by chronic inflammation, autoimmune dysregulation, and metabolic disturbance [66,67], suggesting that pre-existing genetic liabilities may influence susceptibility to Long COVID. Although overlap with other GWAS is limited, this may reflect differences in case definitions, statistical power, tissue models, and control strategies rather than false discoveries. We provide complete results for user inspection, reproducibility, and external validation.

Diagnostic panels that quantify gene expression, combined with machine-learning risk models, could facilitate the early identification of individuals at risk. Drug-repurposing screens that target high-priority driver genes offer a rational route to therapeutic discovery; however, *in vitro*, *in vivo*, and longitudinal validation will be essential before clinical translation.

## Conclusion

Our study presents a reproducible and extensible framework for putative causal gene discovery that integrates genetic, transcriptomic, and network control evidence. Although current Long-COVID GWAS resources are underpowered, the method is intentionally designed to be future-proof. By providing an open platform for iterative refinement, we lay the foundation for a community-based understanding of Long COVID genetics and a path toward precise evidence-based care.

## Supporting information

S1 Text GTEx v8 eQTL datasets. Description of 49 GTEx tissue-specific cis-eQTL datasets (Version 8, Ensembl 99, GRCh38) encompassing 39,832 unique genes from nearly 1,000 healthy European individuals. All associations are significant (FDR < 0.05) within 1Mb of the transcription start site.
(PDF)

S2 Text Details of the Long COVID GWAS dataset. Description of the Long COVID GWAS dataset (Release 7; Ensembl 109; GRCh38) from Lammi et al., 2023, including 3,018 cases evaluated for 19 post-COVID symptoms and 1,093,995 controls across six ancestries. Provides complete lists of ancestries, symptoms, and unique SNPs analyzed.
(PDF)

S3 Text Whole Genome Sequencing (WGS) data for LD matrix calculation. Description of GTEx WGS data (Ensembl 88, GRCh38) containing 820,792 unique SNPs from 836 European individuals used to calculate the linkage disequilibrium (LD) matrix. Details on data access and alternative reference panels matched with ancestry.
(PDF)

S4 Text Mount Sinai COVID-19 Biobank Study. RNA-sequencing Data Description of RNA-sequencing gene expression data (GSE215865, Ensembl GRCh37) from 413 blood samples, including 158 Long COVID individuals (symptoms that persist > 1 month after infection), COVID-19 patients, and healthy controls.
(PDF)

S5 Text Protein-Protein Interaction (PPI) dataset. Description of the human PPI dataset from Vinayagam et al. 2011, used as a model for building the Long COVID network.
(PDF)

S6 Text Integration analysis of polygenic risk score (PRS). Description of integration analysis between 32 putative causal genes of Long COVID and three COVID-19 PRS datasets from the PGS Catalog (PGS002272, PGS002273, and PGS004938). Details on variant-to-gene mapping methodology using TSS-based mapping with LD clumping, statistical enrichment testing, and distance analysis between Long COVID genes and COVID-19 PRS variants.
(PDF)

S7 Text Methodological framework for Long COVID causal gene identification. Complete description of the integrated MR and CT framework for identifying putative causal genes. Includes risk score calculation, network score calculation, final gene ranking methodology, enrichment analysis procedures, clustering, and validation approaches.
(PDF)

S1 Table Shared genetic basis between Long COVID and related conditions. Disease-gene associations compiled from five databases (MalaCards, DISEASES, DISGENET, MedGen, and GenCC) for genes identified in this study. Conditions were selected based on pathophysiological overlap with Long COVID, including immune/inflammatory components, chronic symptoms, multi-system involvement, and metabolic/endocrine disruptions. The table includes condition names, associated genes, primary pathophysiological mechanisms, and database sources.
(XLSX)

S2 Table Instrumental variables (SNPs) for causal gene analysis. Complete list of SNPs used as instrumental variables in the MR analysis for each identified gene. The table includes SNP identifiers, associated genes, and tissue-specific expression data.
(TSV)

S3 Table Literature support for putative causal genes in COVID-19 and Long COVID. Summary of putative causal genes with established links to COVID-19 and hypothesized effects in Long COVID. Includes gene functions, mechanisms, and supporting references.
(MD)

S4 Table Results of enrichment analysis for putative causal genes of Long COVID. Significantly enriched terms and pathways from the GO, KEGG, and Reactome databases. Includes biological processes, molecular functions, cellular components, pathways with associated gene counts, enrichment statistics (p-value, p-adjust, q-value), and gene lists.
(XLSX)

S5 Table Complete clinical data and statistical comparisons for Long COVID clusters. Comprehensive symptom prevalence data for all three Long COVID clusters, including counts, percentages, and statistical test results (Chi-square and Fisher’s exact tests). Contains complete demographic information and all clinical variables analyzed across clusters.
(XLSX)

S6 Table Statistical analysis and multi-network validation. Statistical test results of gene rankings across multiple PPI networks demonstrating framework robustness.
(XLSX)

S7 Table Normalization and analysis of the sensitivity of the parameter *α* Complete results of gene rankings by different normalization methods and values of the parameter *α* (0, 0.25, 0.50, 0.75, 1.0), including sets of the K gene and comparative statistics that demonstrate the flexibility of the framework.
(CSV)

S1 Fig Network plots for Long COVID driver genes. Network visualizations for all identified protein-coding genes of the network driver showing protein-protein interactions, connectivity patterns, and network topology for each gene. (PDF)

S2 Fig Sample-level gene expression heatmap for Long COVID clusters. Individual patient-level heatmap showing expression patterns of the 32 candidate Long COVID genes across all samples. Color gradient represents z-scored log_2_ expression values with hierarchical clustering of both genes and samples.
(PDF)

S3 Fig Multi-network validation visualization. Comparative visualization of gene rankings and controllability scores across multiple PPI networks, demonstrating framework robustness and network-specific differences in topology and interaction coverage.
(PDF)

S4 Fig Visualization of gene ranking in normalization methods. Visualization of the consistency of gene ranking across different normalization approaches and parameter settings (*α*), spanning from statistical causal evidence to network-based prioritization.
(PDF)
