# Supplementary material for: Integrative multi-omics framework for causal gene discovery in Long COVID
Source: PLoS Comput Biol. 2025 Dec 1;21(12):e1013725. doi: 10.1371/journal.pcbi.1013725 (PMC12677781; doi:10.1371/journal.pcbi.1013725)
Supplement: S1 Text — Description of 49 GTEx tissue-specific cis-eQTL datasets (Version 8, Ensembl 99, GRCh38) encompassing 39,832 unique genes from nearly 1,000 healthy European individuals. All associations are significant (FDR < 0.05) within 1Mb of the transcription start site. (PDF) [file pcbi.1013725.s001.pdf]

S1 Text: Expression Quantitative Trait Loci (eQTL)

Table 1 summarizes the expression Quantitative Trait Loci (eQTL) datasets used in this study, obtained from the Genotype-Tissue Expression (GTEx) project (Version 8, Ensembl 99, GRCh38) [1]. These datasets provide a comprehensive resource for understanding the relationship between genetic variants and gene expression levels across 49 distinct human tissues. For each tissue, the table lists the number of samples, unique genes, and gene-SNP associations analyzed, reflecting the depth and breadth of the GTEx project.

The tissues include a wide range of systems, such as the nervous system (e.g., amygdala, cortex, hippocampus), cardiovascular system (e.g., aorta artery, coronary artery, left ventricle), digestive system (e.g., stomach, esophagus, colon), and others. The number of samples varies across tissues, with skeletal muscle having the largest sample size (706), while kidney cortex has the smallest (73). The diversity of tissues and sample sizes ensures robust tissue-specific gene expression regulation exploration.

These datasets are particularly valuable for identifying regulatory variants that affect gene expression tissue-dependently. This enables the integration of genetic and transcriptomic data, which is critical for finding mechanisms underlying complex traits and diseases, including Long COVID. For instance, lung, blood, and brain tissues are particularly interesting in this study due to their relevance to Long COVID symptoms, ranging from respiratory issues to neurological and systemic effects.

References

[1] GTEx portal - datasets (2023). URL https://gtexportal.org/home/datasets. Accessed 8 Sep 2023.

Table 1: **Summary of Expression Quantitative Trait Loci (eQTL) datasets**. It indicates the number of samples, unique genes, and gene-SNPs associations for 49 distinct tissues, obtained from the GTEx project (Version 8, Ensembl 99, GRCh38) [1].

**Tissue**	**Samples**	**Genes**	**SNPs**
Adrenal gland	233	23,820	23,264
Amygdala brain	129	24,069	23,609
Anterior cingulate cortex	147	24,342	23,843
Aorta artery	387	23,959	23,371
Atrial appendage	372	23,194	22,747
Breast mammary tissue	396	25,849	25,294
Caudate (basal ganglia)	194	24,718	24,323
Cerebellar hemisphere	175	25,144	24,404
Cerebellum	209	25,461	24,737
Coronary artery	213	24,529	24,095
Cortex	205	24,849	24,419
Cultured fibroblasts	483	22,050	21,416
EBV-transformed lymphocytes	147	22,759	22,199
Esophagus mucosa	497	23,949	23,340
Esophagus muscularis	465	23,871	23,288
Frontal cortex	175	24,676	24,265
Gastroesophageal junction	330	24,168	23,634
Hippocampus	165	24,420	24,087
Hypothalamus	170	25,096	24,649
Kidney cortex	73	24,807	24,395
Left ventricle	386	21,353	20,991
Liver	208	22,262	21,870
Lung	515	26,095	25,464
Minor salivary gland	144	25,579	25,020
Not sun-exposed skin (suprapubic)	517	25,279	24,676
Nucleus accumbens (basal ganglia)	202	24,890	24,463
Ovary	167	25,325	24,792
Pancreas	305	22,615	22,129
Pituitary	237	26,854	26,218
Prostate	221	26,529	25,969
Putamen (basal ganglia)	170	23,804	23,428
Sigmoid colon	318	24,483	23,951
Skeletal muscle	706	21,031	20,560
Small intestine terminal ileum	174	26,182	25,694
Spinal cord	126	24,669	24,167
Spleen	227	25,479	24,900
Stomach	324	24,290	23,862
Subcutaneous adipose	581	24,665	24,010
Substantia nigra	114	24,044	23,626
Sun-exposed skin (lower leg)	605	25,196	24,564
Testis	322	35,007	34,164
Thyroid	574	26,054	25,184
Tibial artery	584	23,304	22,652
Tibial nerve	532	25,873	25,092
Transverse colon	368	25,379	24,816
Uterus	129	25,188	24,637
Vagina	141	25,778	25,245
Visceral omentum adipose	469	24,724	24,167
Whole blood	670	20,315	19,701
