# Supplementary material for: Integrative multi-omics framework for causal gene discovery in Long COVID
Source: PLoS Comput Biol. 2025 Dec 1;21(12):e1013725. doi: 10.1371/journal.pcbi.1013725 (PMC12677781; doi:10.1371/journal.pcbi.1013725)
Supplement: S2 Text — Description of the Long COVID GWAS dataset (Release 7; Ensembl 109; GRCh38) from Lammi et al., 2023, including 3,018 cases evaluated for 19 post-COVID symptoms and 1,093,995 controls across six ancestries. Provides complete lists of ancestries, symptoms, and unique SNPs analyzed. (PDF) [file pcbi.1013725.s002.pdf]

S2 Text: Genome-wide Association Studies (GWAS)

Description

Table 1 summarizes the Genome-wide Association Studies (GWAS) datasets available from Lammi et al., 2023 [1], which include genetic data for Long COVID cases and controls categorized into broad and strict definitions. Broad cases refer to Long COVID patients tested or untested for SARS-CoV-2, while strict cases are those with confirmed Long COVID based on SARS-CoV-2 test verification. Similarly, broad controls are from the general population, and strict controls are SARS-CoV-2-positive individuals who did not develop Long COVID.

In this study, we used only GWAS1 for the Mendelian Randomization (MR) analysis. GWAS1 pairs strictly verified Long COVID cases with broad controls, making it the most appropriate choice for this analysis. The strict case definition reduces misclassification bias, ensuring that the genetic associations identified are specific to Long COVID. Broad controls provide a larger sample size, increasing the analysis's statistical power.

The other datasets, GWAS2, GWAS3, and GWAS4, were not used as they either relaxed the definition of cases or limited the control group. For instance, GWAS2 includes broad cases, which may introduce noise into the analysis. GWAS3 and GWAS4 use strict controls, which, while specific, result in smaller sample sizes, reducing statistical power. GWAS1 was, therefore, the optimal choice for this study, as it strikes a balance between specificity in cases and a sufficient control group size to support reliable causal inference.

Table 1: **Genome-wide Association Studies (GWAS) datasets used in this study [1].** Release: 7, Ensembl: 109, Human Genome Build: GRCh38. **Broad Cases** refers to Long COVID cases that were tested and untested for SARS-CoV-2 infection. **Strict Cases** refers to Long COVID cases that were only test-verified for SARS-CoV-2 infection. **Broad Controls** are from the general population, while **Strict Controls** are SARS-CoV-2 cases that did not develop Long COVID.

**Dataset**	**Cases**	**Controls**	**SNPs**
GWAS 1	Strict: 3,018	Broad: 1,093,995	9,510,587
GWAS 2	Broad: 6,450	Broad: 1,093,995	9,722,678
GWAS 3	Strict: 3,018	Strict: 46,208	9,738,584
GWAS 4	Broad: 6,450	Strict: 46,208	9,753,825
TOTAL	Unique: 6,450	Unique: 1,093,995	9,722,678

Figure 1 illustrates the distribution of cases and controls across the four Long COVID GWAS datasets sourced from Lammi et al., 2023 [1]. The datasets distinguish between **Broad Cases**, which include Long COVID patients regardless of SARS-CoV-2 testing status, and **Strict Cases**, which consist only of test-verified Long

COVID patients. Similarly, **Broad Controls** are drawn from the general population, while **Strict Controls** are SARS-CoV-2-positive individuals who did not develop Long COVID. For this study, GWAS1 was selected due to its use of strictly verified Long COVID cases combined with broad population controls, providing the necessary specificity and statistical power for robust analysis.

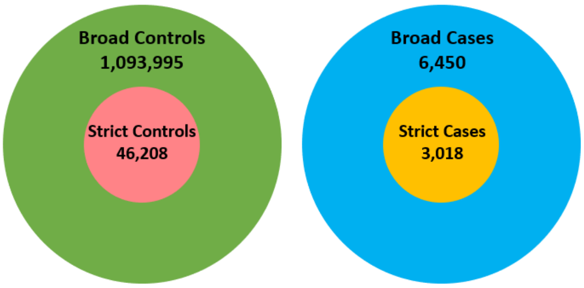

Fig. 1: **Cases and controls for the four Long COVID GWAS datasets used in the analysis and sourced from Lammi et al., 2023 [1]. Broad Cases** refers to Long COVID cases that were both tested and untested for SARS-CoV-2 infection. **Strict Cases** refers to Long COVID cases that were only test-verified for SARS-CoV-2 infection. **Broad Controls** are from the general population, while **Strict Controls** are SARS-CoV-2 cases that did not develop Long COVID.

Example

Table 2 presents the top five rows from one of the original Long COVID GWAS datasets described by Lammi et al., 2023 [1]. Each row represents a genetic variant with its associated details, including the chromosome number (**Chr**), genomic position (**Position**), unique variant identifier (**Variant ID**), reference allele (**Ref All**), alternate allele (**Alt All**), log odds ratio (**logOR**), effect size estimate (**Beta**), standard error of the effect size (**SE**), and frequency of the alternate allele (**Freq**). These data highlight key attributes of the genetic variants that were used to identify potential associations with Long COVID phenotypes. The log odds ratio (**logOR**) and effect size estimate (**Beta**) provide insights into the direction and magnitude of the variant's impact. In contrast, the standard error (**SE**) reflects the variability in these estimates. The frequency of the alternate allele (**Freq**) aids in understanding the distribution of genetic variation within the population.

Symptoms and Ancestries

The symptoms and ancestries represented in the Long COVID GWAS1 dataset used in this study reflect the diverse clinical presentations and populations affected by Long COVID, as summarized in Table .

Table 2: **Top 5 rows from one of the original Long COVID GWAS datasets** [1]. It shows the chromosome number, the variant's genomic position, the genetic variant's unique identifier, the reference and alternate alleles, the log odds ratio, the effect size estimate, the standard error of the effect size, and the frequency of the alternate allele.

Chr	Position	Variant ID	Ref All	Alt All	logOR	Beta	SE	Freq
1	727242	rs61769339	G	A	0.660	−0.0891	0.0725	0.142
1	729886	rs539032812	T	C	0.204	−0.0856	0.175	0.0278
1	758351	rs12238997	A	G	0.642	−0.0838	0.0695	0.150
1	758443	rs61769351	G	C	0.462	−0.0672	0.0712	0.148
1	770988	rs12029736	A	G	0.332	0.0452	0.0619	0.491

The dataset includes a comprehensive list of symptoms commonly reported by Long COVID patients. These symptoms span various systems and manifestations, highlighting the condition's heterogeneity. Key symptoms include fatigue, shortness of breath, memory and concentration problems, anosmia, persistent cough, and insomnia. Symptoms affecting other systems, such as gastrointestinal issues (e.g., abdominal pain, nausea/vomiting, diarrhea) and musculoskeletal complaints (e.g., myalgia, arthralgia), are also represented.

Moreover, the GWAS1 dataset has individuals from six major ancestry groups: Mixed American, African, East Asian, European, Middle Eastern, and South Asian. This broad representation ensures that findings are inclusive and applicable across diverse populations. By considering multiple ancestries, the study minimizes the risk of population-specific bias and enhances the generalizability of the results. Furthermore, this diversity is crucial for understanding how genetic factors may influence Long COVID risk and symptoms differently across populations.

Symptoms:

- Abdominal pain
- Anosmia
- Arthralgia
- Chest pain
- Chills
- Confusion/Disorientation
- Depression
- Diarrhea
- Dysphagia
- Fatigue
- Fever
- Headache
- Hoarseness
- Insomnia
- Myalgia
- Nausea/Vomiting
- Numbness/Tingling
- Persistent cough
- Problems with memory/concentration
- Reduced appetite
- Rhinorrhea
- Shortness of breath
- Sore throat
- Weight loss

Ancestries:

- Admixed American
- African
- East Asian
- European
- Middle Eastern
- South Asian

References

[1] Lammi, V. *et al.* Genome-wide association study of long covid. *Nature Genetics* **57**, 1402–1417 (2025). Epub 2025 May 21.
