# Supplementary material for: Integrative multi-omics framework for causal gene discovery in Long COVID
Source: PLoS Comput Biol. 2025 Dec 1;21(12):e1013725. doi: 10.1371/journal.pcbi.1013725 (PMC12677781; doi:10.1371/journal.pcbi.1013725)
Supplement: S3 Text — Description of GTEx WGS data (Ensembl 88, GRCh38) containing 820,792 unique SNPs from 836 European individuals used to calculate the linkage disequilibrium (LD) matrix. Details on data access and alternative reference panels matched with ancestry. (PDF) [file pcbi.1013725.s003.pdf]

# S3 Text: Whole Genome Sequence (WGS) Data for LD Analysis

Table 1 shows the top five rows from the Whole Genome Sequence (WGS) BIM file used to calculate the Linkage Disequilibrium (LD) matrix, sourced from the GTEx project (Ensembl 88, GRCh38) [1]. This file contains information about genetic variants, including the chromosome number (**Chr**), variant identifier (**Variant ID**), distance from the start of the chromosome (**Distance**), genomic position on the chromosome (**Position**), and the reference (**Ref all**) and alternate alleles (**Alt all**).

The provided rows demonstrate the structure and organization of the WGS BIM file. For example, the first row describes a variant located at position 13,526 on chromosome 1, with a reference allele of T and an alternate allele of C. This dataset forms the foundation for calculating LD matrices, which are crucial for understanding the correlation between genetic variants and their co-inheritance patterns.

**Table 1**: **Top 5 rows of the Whole Genome Sequence (WGS) BIM file.** This dataset was used for calculating the Linkage Disequilibrium (LD) matrix. The table provides details on the chromosome, variant ID, distance from the start of the chromosome, position on the chromosome, and reference and alternate allele. It was sourced from GTEx (Ensembl 88, GRCh38) [1].

| Chr | Variant ID | Distance | Position | Ref all | Alt all |
|---|---|---|---|---|---|
| 1 | chr1_13526_C_T_b38 | 0 | 13526 | T | C |
| 1 | chr1_13550_G_A_b38 | 0 | 13550 | A | G |
| 1 | chr1_14451_CTCT_C_b38 | 0 | 14451 | C | CTCT |
| 1 | chr1_14469_C_T_b38 | 0 | 14469 | T | C |
| 1 | chr1_14470_G_A_b38 | 0 | 14470 | A | G |

Table 2 presents the top five rows from the GWS FAM file, containing metadata for the 836 European individuals used in calculating the LD matrix, sourced from GTEx (Ensembl 88, GRCh38) [1]. The table includes the family ID (**Family ID**), individual ID (**Individual ID**), paternal and maternal IDs (**Paternal ID** and **Maternal ID**), sex (**Sex**, where 1 represents male and 2 represents female), and phenotype status (**Phenotype**, with -9 indicating missing phenotype data).

This metadata ensures the accurate identification of individuals and their relationships, which is essential for LD matrix calculations. The uniform phenotype status (-9) reflects the absence of case/control definitions in this dataset, as it is primarily intended for population-level analyses.

# References


[1] GTEx portal - protected data access (2023). URL https://gtexportal.org/home/protectedDataAccess. Accessed: 09/08/2023.

**Table 2**: **Top 5 rows of the Whole Genome Sequence (GWS) FAM file**. This dataset has 836 European male and female individuals and it was used for calculating the Linkage Disequilibrium (LD) matrix, sourced from Genotype-Tissue Expression (GTEx) (Ensembl 88, GRCh38) [1]. The table provides details on the family ID, individual ID, paternal and maternal IDs, gender, and phenotype status.

| Family ID | Individual ID | Paternal ID | Maternal ID | Sex | Phenotype |
|---|---|---|---|---|---|
| GTEX-1117F | GTEX-1117F | 0 | 0 | 2 | -9 |
| GTEX-111CU | GTEX-111CU | 0 | 0 | 1 | -9 |
| GTEX-111FC | GTEX-111FC | 0 | 0 | 1 | -9 |
| GTEX-111VG | GTEX-111VG | 0 | 0 | 1 | -9 |
| GTEX-111YS | GTEX-111YS | 0 | 0 | 1 | -9 |
