# Supplementary material for: Integrative multi-omics framework for causal gene discovery in Long COVID
Source: PLoS Comput Biol. 2025 Dec 1;21(12):e1013725. doi: 10.1371/journal.pcbi.1013725 (PMC12677781; doi:10.1371/journal.pcbi.1013725)
Supplement: S4 Text — RNA-sequencing Data Description of RNA-sequencing gene expression data (GSE215865, Ensembl GRCh37) from 413 blood samples, including 158 Long COVID individuals (symptoms that persist > 1 month after infection), COVID-19 patients, and healthy controls. (PDF) [file pcbi.1013725.s004.pdf]

S4 Text: RNA-seq Gene Expression and Clinical Data

The participants from the RNA-sequencing (RNA-seq) dataset used in this study [1], totaling 567 individuals (both males and females), have an age range from 0 to 90 years and are represented by a diverse racial background as follows :

- Black or African American
- Asian
- White
- American Indian/Alaska Native
- Native Hawaiian or Other Pacific Islander
- Individuals identifying with multiple races

Table 1 presents the top five rows and columns of the RNA-seq gene expression dataset used in this study, sourced from the Gene Expression Omnibus (GEO) - National Center for Biotechnology Information (NCBI) database (GSE215865, Ensembl GRCh37) [1]. This dataset includes gene expression measurements for 58,884 unique genes across a cohort of 567 participants, consisting of 495 acute and Long COVID patients and 72 controls. The participant's age range is between 0 to 90 years, and represents diverse racial backgrounds, ensuring the dataset reflects population heterogeneity.

The table highlights the Ensembl Gene IDs and their corresponding expression values for a subset of samples. Missing expression values (**NA**) are present for some genes, reflecting the sparsity often observed in RNA-seq data for lowly expressed or unexpressed genes in specific samples. For instance, *ENSG00000227232.5* has measurable expression across most samples, while other genes, such as *ENSG00000223972.5* and *ENSG00000243485.5*, show no detectable expression in the displayed rows.

To ensure the dataset was suitable for downstream analyses, rows and columns containing only **NA** values were deleted. For the remaining missing values, the mean of the respective gene or sample was used to impute missing expression levels, ensuring a complete dataset while minimizing potential biases.

This RNA-seq dataset forms the basis for transcriptomic analyses conducted in this study, helping identify key genes and pathways associated with Long COVID and acute COVID conditions.

References

[1] NCBI GEO - GSE215865 (2023). URL https://www.ncbi.nlm.nih.gov/geo/query/acc.cgi?acc=GSE215865. Accessed 11 Feb 2023.

Table 1: Top 5 rows and columns of the RNA-sequencing (RNA-seq) gene expression dataset used in this paper. This dataset consists of gene expression data from 495 acute and Long COVID patients and 72 controls, covers 58,884 unique genes, and includes data from a diverse cohort of 567 participants of varying ages (0 to 90 years) and multiple racial backgrounds. The table showcases the Ensembl Gene ID and expression values for a subset of the participants. It was sourced from the GEO - NCBI database (GSE215865, Ensembl GRCh37) [1].

**Gene ID**	**Subj1_Sample1**	**Subj1_Sample2**	**Subj2_Sample1**	**Subj2_Sample2**
ENSG00000223972.5	NA	NA	NA	NA
ENSG00000227232.5	NA	1.415	1.499	1.706
ENSG00000278267.1	NA	-0.669	-0.858	0.229
ENSG00000243485.5	NA	NA	NA	NA
ENSG00000284332.1	NA	NA	NA	NA
