# Supplementary material for: Integrative multi-omics framework for causal gene discovery in Long COVID
Source: PLoS Comput Biol. 2025 Dec 1;21(12):e1013725. doi: 10.1371/journal.pcbi.1013725 (PMC12677781; doi:10.1371/journal.pcbi.1013725)
Supplement: S5 Text — Description of the human PPI dataset from Vinayagam et al. 2011, used as a model for building the Long COVID network. (PDF) [file pcbi.1013725.s005.pdf]

S5 Text: Protein-Protein Interaction (PPI)

Table 1 displays the top five rows from the Protein-Protein Interaction (PPI) dataset used to construct the Long COVID network, adapted from Vinayagam et al., 2011 [1]. This dataset provides insights into the functional relationships between genes based on their interactions within biological networks. The table includes the gene identifiers (**Gene ID**), gene names (**Gene Name**), and their classification into node subtypes (**Node Subtype**).

Node subtypes categorize genes based on their role and resilience within the network:

- **Type I genes:** Highly connected and robust against changes or disruptions, representing critical hubs within the network.
- **Type II genes:** Less connected and more susceptible to minor network disruptions, making them potential points of vulnerability in the system.

For example, *CREBBP*, classified as a Type I gene, is a key regulatory hub with significant connectivity, suggesting its role in maintaining network stability. In contrast, *HMOX2* and *GBP2*, both Type II genes, exhibit less resilience and may serve as potential targets for understanding network fragility in Long COVID pathophysiology.

This PPI dataset is integral to the construction of a robust Long COVID interaction network, providing a framework to study gene-level contributions to disease mechanisms and their potential as therapeutic targets.

Table 1: **Top 5 rows of the Protein-Protein Interaction (PPI) dataset used for building the Long COVID network.** The table showcases the genes and their associated subtypes. Type-I genes exhibit greater resilience and have more connections, being more robust against network alterations, while Type-II genes are more susceptible to even minor network disruptions. This dataset was sourced from Vinayagam et al., 2011 [1].

Gene ID	Gene Name	Node Subtype
3163	HMOX2	Type II
1387	CREBBP	Type I
2634	GBP2	Type II
5499	PPP1CA	Type I
6642	SNX1	Type II

References

[1] Vinayagam, A. *et al.* A directed protein interaction network for investigating intracellular signal transduction. *Science Signal* **4** (2011).
