# Supplementary material for: Integrative multi-omics framework for causal gene discovery in Long COVID
Source: PLoS Comput Biol. 2025 Dec 1;21(12):e1013725. doi: 10.1371/journal.pcbi.1013725 (PMC12677781; doi:10.1371/journal.pcbi.1013725)
Supplement: S6 Text — Description of integration analysis between 32 putative causal genes of Long COVID and three COVID-19 PRS datasets from the PGS Catalog (PGS002272, PGS002273, and PGS004938). Details on variant-to-gene mapping methodology using TSS-based mapping with LD clumping, statistical enrichment testing, and distance analysis between Long COVID genes and COVID-19 PRS variants. (PDF) [file pcbi.1013725.s006.pdf]

S6 Text: Polygenic Risk Score Integration Analysis

Methodology

We conducted a comprehensive integration analysis between our 32 Long COVID putative causal genes and existing COVID-19 polygenic risk scores (PGS) from the PGS Catalog. Our methodology employed:

TSS-based mapping: Variants were mapped to genes using transcription start site (TSS) proximity (±50kb window) with strand-aware annotations from UCSC RefSeq (GRCh38, 28,307 genes).

LD clumping: Applied 200kb distance-based clumping to remove correlated variants and prevent signal inflation, retaining the strongest variant per locus.

Dataset-specific filtering: For PGS004938, we applied 97.5th percentile filtering to retain 9,500 high-confidence variants from 955,417 total variants.

Sensitivity analyses: Multiple parameter combinations tested including window sizes (50kb vs 100kb), tie-handling policies (up to 3 nearest genes vs strict nearest-only), and clumping distances (100kb vs 200kb).

Results Summary

Primary analysis identified 3,190 unique genes from all PGS datasets combined. Only 3 genes (9.4%) overlapped with our Long COVID gene set: *ITPRID1* (distance: 4.4kb), *GRB2* (5.6kb), and *CDA* (8.5kb). Statistical enrichment was non-significant ($p = 0.72$, Fisher's exact test).

Distance diagnostic revealed:

- Within 50kb of PGS variants: 7/32 genes (22%)
- Within 100kb of PGS variants: 11/32 genes (34%)
- Beyond 100kb: 21/32 genes (66%)

Sensitivity analyses confirmed robustness across parameter variations, with overlap remaining consistently minimal (2-4 genes across all tested configurations).

Biological Interpretation

The limited cis-regulatory overlap (9.4%) contrasted with strong literature validation (59.4% of our genes confirmed in COVID-19/Long COVID studies) suggests Long COVID operates through distinct genetic mechanisms:

Trans-regulatory effects: Most Long COVID genes likely influence disease through network-mediated or long-range regulatory mechanisms not captured by proximity-based PGS mapping.

Different pathophysiology: The genetic architecture underlying persistent post-viral symptoms appears fundamentally distinct from acute COVID-19 susceptibility mechanisms.

Network-mediated causality: Our Control Theory analysis identified 16 network driver genes (K-degree: 151-299 connections) that may regulate disease through pathway control rather than direct cis-effects.
