# Supplementary material for: Integrative multi-omics framework for causal gene discovery in Long COVID
Source: PLoS Comput Biol. 2025 Dec 1;21(12):e1013725. doi: 10.1371/journal.pcbi.1013725 (PMC12677781; doi:10.1371/journal.pcbi.1013725)
Supplement: S7 Text — Complete description of the integrated MR and CT framework for identifying putative causal genes. Includes risk score calculation, network score calculation, final gene ranking methodology, enrichment analysis procedures, clustering, and validation approaches. (PDF) [file pcbi.1013725.s007.pdf]

Framework

Overview

Figure 1 illustrates the first part of the analytical framework employed in this study, outlining the relationship between genetic variants, gene expression, and Long COVID outcomes. The framework integrates three key components:

- **Instrumental Variables (IVs):** Genetic variants (SNPs) that serve as instruments to explore causal relationships.
- **Exposure:** Gene expression levels (eQTL) representing the intermediate step between genetic variants and the outcome.
- **Outcome:** Long COVID phenotypes derived from GWAS datasets.

The figure also highlights the presence of potential confounders that may simultaneously influence gene expression and Long COVID outcomes. This design adheres to the MR, which uses genetic variants as natural experiments to infer causal relationships, minimizing confounding and reverse causation.

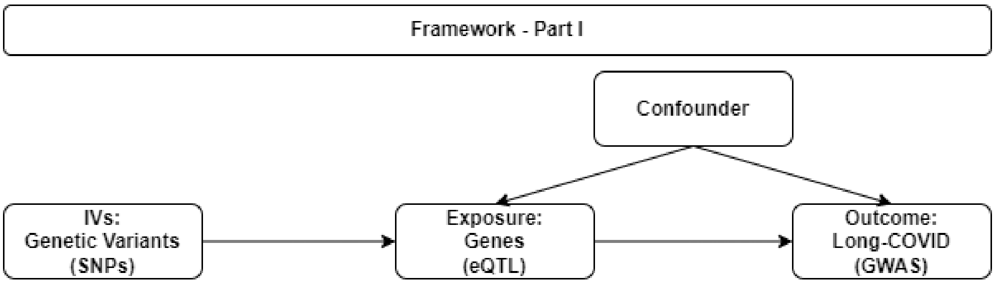

Fig. 1: **Mendelian Randomization (MR) Framework - Part I.** This framework applies MR to identify causal relationships between genetic variants (instrumental variables, IVs), gene expression (eQTL) as exposures, and Long COVID Genome-Wide Association Studies (GWAS) as outcomes. The model accounts for confounding factors to ensure robust causal inference, establishing a pathway from genetic variants through eQTL to Long COVID outcomes.

Figure 1 outlines the second part of the framework, which details two procedural approaches for identifying causal and critical genes in Long COVID.

- **Procedure I:**
  - Combines eQTL data and GWAS results to perform MR using the `Mt_Robin` tool, identifying causal genes associated with Long COVID.
  - RNA-seq gene expression data and PPI networks are analyzed using Control Theory (CT), specifically the Controllability Analysis (CA) method to identify critical genes within the Long COVID network.

- The overlap between causal and critical genes provides a set of biologically significant targets for Long COVID.

- **Procedure II:**
  - Begins with MR to identify causal genes.
  - These genes are filtered and further analyzed using RNA-seq and PPI network data.
  - CT is applied to identify critical genes, producing a refined set of causal and critical genes for Long COVID.

This framework integrates MR and network controllability to identify and prioritize key genes comprehensively, bridging genetic causality with network-level importance in Long COVID research.

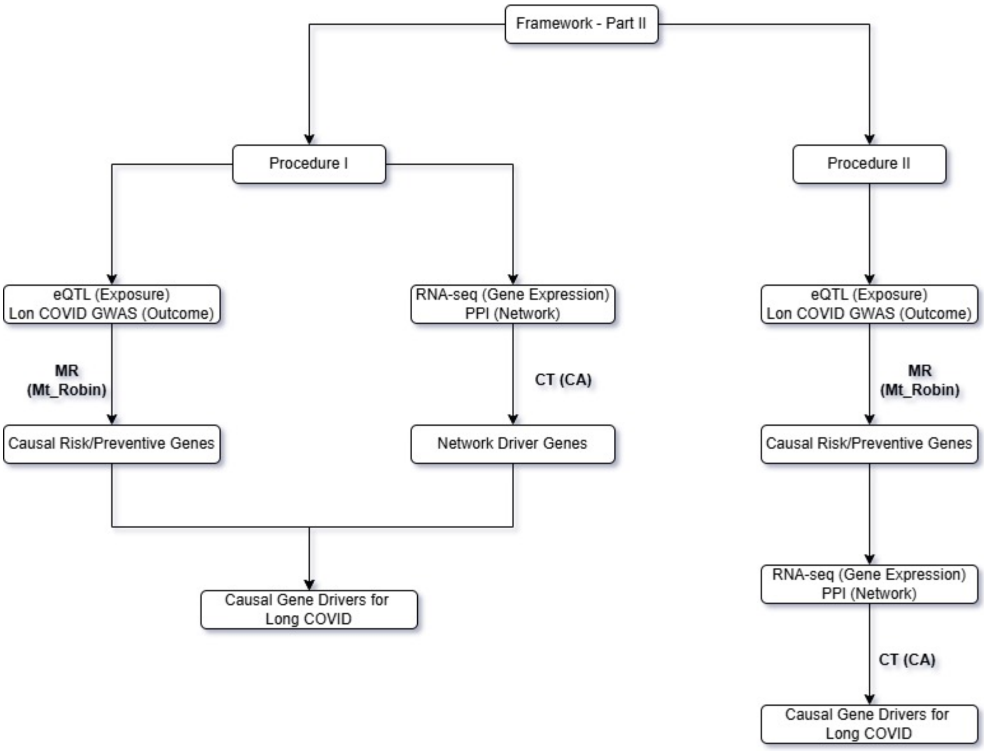

Integrative Multi-Omics Framework for Causal Gene Discovery in Long COVID - Part II. This framework combines two procedures to identify causal gene drivers for Long COVID by integrating genetic and transcriptomic data. Procedures I and II use expression Quantitative Trait Loci (eQTL) as exposures and Long COVID Genome-Wide Association Studies (GWAS) as outcomes, using Mendelian Randomization (MR) implemented via Mt-Robin to identify causal risk/preventive genes, and RNA sequencing (RNA-seq) data and Protein-Protein Interaction (PPI) networks, applying Controllability Analysis (CA), a kind of Control Theory (CT) method, to detect network driver genes. These steps collectively converge to uncover causal gene drivers for Long COVID.

Mendelian Randomization (MR): Mt-Robin

The Multi-tissue transcriptome-wide Mendelian Randomization method ROBust to INvalid instrumental variables (Mt-Robin) analysis for GWAS1 [1], and eQTL [2] represents a comprehensive approach to identifying causal relationships between genetic variants and Long COVID susceptibility. This analysis integrates GWAS data with eQTL information to detect potential causal genes.

0.0.1 Data Pre-processing

Overview

Data pre-processing for the Long COVID GWAS analysis involved multiple steps designed to ensure data quality, compatibility, and reliability. This process was essential for preparing the data for downstream MR analysis using the Mt-Robin method. The pre-processing workflow consisted of several key stages:

Data Cleaning

- Removal of missing or incomplete data entries
- Standardization of genetic variant identifiers
- Quality control of allele frequencies and effect sizes
- Verification of data consistency across files

Data Format Standardization

- Standardization of column names across datasets
- Conversion of data types to appropriate formats
- Organization of variant information in a consistent structure
- Implementation of uniform coordinate systems

Quality Control Measures

- Verification of variant ID consistency
- Cross-reference of allele frequencies
- Assessment of effect size distributions
- Evaluation of standard error measurements
- Check for duplicate entries

Forward Selection Analysis

Forward selection represents a stepwise approach to identifying instrumental variables while controlling for LD and expression patterns.

Instrumental Variable Selection

The selection process employs three key parameters:

$$\begin{aligned} \text{ld\_thresh} &= 0.5, \\ \text{pval\_thresh} &= 0.001, \\ \text{nTiss\_thresh} &= 1. \end{aligned} \tag{1}$$

where:

- ld_thresh: Maximum allowed linkage disequilibrium (LD) between SNPs to ensure independence.
- pval_thresh: Threshold for the p-value to select significant SNPs.
- nTiss_thresh: Minimum number of tissues where SNP expression is observed to retain significance.

These thresholds ensure a robust selection of independent genetic instruments while maintaining biological relevance.

Selection Process

The iterative selection process follows a structured approach:

1. **SNP Pool Creation:** The initial pool is formed from SNPs meeting the specified p-value threshold.
2. **LD Matrix Calculation:** Linkage Disequilibrium (LD) is computed using PLINK format files:

   $$\text{LD} = \text{cor}(\text{df\_subset, use=”pairwise.complete”}) \tag{2}$$

   The squared correlation coefficient is then calculated as:

   $$\text{LD\_r2} = \text{LD}^2 \tag{3}$$

   where:

   - LD: Linkage Disequilibrium calculated as the correlation between genetic variants.
   - df_subset: Subset of the data frame containing the genetic variant data.
   - LD_r2: Squared correlation coefficient representing the strength of LD.

3. **Iterative Selection:** SNPs are iteratively selected based on:

   - The minimum p-value in the remaining pool.
   - LD threshold constraints to ensure independence.
   - Tissue-specific expression patterns to prioritize biologically relevant SNPs.

This systematic approach ensures the selection of independent and biologically relevant genetic instruments.

Mt-Robin Setup

The Mt-Robin setup phase establishes the framework for analyzing causal relationships between genetic variants and disease outcomes.

Data Preparation

The setup process involves:

1. Loading eQTL and GWAS data
2. Matching SNPs across datasets
3. Extracting beta values and standard errors
4. Organizing tissue-specific expression data

This careful preparation ensures data compatibility and reliability for the subsequent analysis.

Mixed Model Implementation

The linear mixed model implements a sophisticated statistical framework:

$$\text{beta\_x} = \text{beta\_y} + (\text{beta\_y}|\text{snpID}) + \epsilon \tag{4}$$

where:

- beta_x: eQTL effect sizes.
- beta_y: GWAS effect sizes.
- snpID: Random effects grouping.
- ϵ: Error term.

This model structure captures both fixed and random effects while accounting for the hierarchical nature of genetic data.

Statistical Analysis

The statistical analysis phase ensures robust identification of significant associations while controlling for multiple testing.

P-value Calculation

P-values are computed through a rigorous process:

1. Bootstrap resampling (10,000 iterations)
2. Null distribution generation
3. Two-sided test comparison

This approach provides reliable statistical inference while accounting for the complex structure of genetic data.

Multiple Testing Correction

Multiple testing correction is implemented using the Benjamini-Hochberg procedure:

$$\text{FDR} = \text{p.adjust}(\text{p\_values, method} = \text{"BH"}) \tag{5}$$

where:

- FDR: False Discovery Rate, representing the expected proportion of false positives among the declared significant results.
- p.adjust: Function used to adjust p-values for multiple testing.
- p_values: The original p-values from statistical tests.
- method = "BH": Benjamini-Hochberg method, a procedure for controlling the False Discovery Rate.

This correction controls the false discovery rate while maintaining reasonable statistical power.

Output Processing

The final results compilation includes comprehensive information for downstream analysis:

- Gene identifiers: Unique identifiers for each analyzed gene
- Effect sizes: Quantification of genetic effects
- Standard errors: Measures of estimation precision
- P-values and FDR: Statistical significance measures
- Number of instrumental variables: Count of independent genetic instruments
- Tissue-specific information: Expression patterns across tissues

This rich output facilitates detailed interpretation and follow-up analyses of identified associations.

Conclusion

The Mt-Robin analysis for GWAS1 and eQTL represents a comprehensive approach to identifying causal genetic factors in Long COVID susceptibility. Through careful data processing, robust statistical analysis, and stringent quality control, this analysis provides valuable insights into the genetic architecture of Long COVID while maintaining high statistical rigor and biological relevance. The complete code for this analysis is available in Notebook 1.

Control Theory (CT): Controllability Analysis (CA)

Clinical Data Processing

We generated four distinct subsets from the main clinical dataset [3]:

- PASC case subset
- Positive Acute COVID control subset
- Negative Acute COVID control subset
- Unknown Acute COVID control subset

Gene Expression Processing

Missing Value Treatment

We processed the gene expression matrix by:

- Removing columns containing only NA values
- Eliminating rows where all values except the first column were NA

Gene Identifier Processing

The gene identifiers were processed through the following sequential steps:

1. Removal of version numbers from Ensembl Gene IDs
2. Mapping to external gene names via biomaRt
3. Addition of gene type information
4. Filtering to retain only protein-coding genes

Control Theory Implementation

Procedure 1: Direct Control Theory Application

1. Applied Control Theory directly to PASC case data
2. Overlapped results with GWAS1 findings

Procedure 2: Overlap-First Approach

1. Overlapped PASC case data with GWAS1 results
2. Applied Control Theory to the overlapped dataset

Network Controllability Analysis

Type I Node Classification

Nodes were classified based on their impact on driver nodes (N_D):

$$\text{Node Type I} = \begin{cases} \text{Critical} & \text{if } N_D \text{ increases in absence} \\ \text{Redundant} & \text{if } N_D \text{ decreases in absence} \\ \text{Ordinary} & \text{if } N_D \text{ remains unchanged} \end{cases}$$

Type II Node Classification

Nodes were classified based on their presence in driver node sets:

$$\text{Node Type II} = \begin{cases} \text{Critical} & \text{if in all driver node sets} \\ \text{Redundant} & \text{if in no driver node sets} \\ \text{Ordinary} & \text{if in some driver node sets} \end{cases}$$

Network Metrics

Topological Measures

We calculated the following network metrics:

- Total number of nodes (N)
- Total number of edges (E)
- Average degree ($\langle k \rangle$)
- Number of driver nodes (N_d)

Mt-Robin Result Integration

Integration Analysis

We performed an analysis with the results from the Mt-Robin results to identify:

- Driver nodes overlapping
- Network properties implicated genes
- Critical control points

Conclusion

The control theory analysis framework presented here combines sophisticated network analysis with biological data integration to identify critical control points in Long COVID gene networks. Through systematic data processing, implementation of two complementary procedures, and comprehensive network metric calculations, this approach provides a robust methodology for understanding network controllability in the context of Long COVID pathogenesis. By integrating Mt-Robin results with network controllability analysis, we established a bridge between genetic association signals and their functional implications in network control. The complete analysis pipeline is available in Notebook 2, ensuring reproducibility and transparency of our methodological approach.

Integration

MR Score

MR analysis results were processed to include all significant causal genes with their respective beta values, p-values, and FDR scores. A normalized MR score (MR_Score_{norm}) was calculated as:

$$MR\_Score_{norm} = \frac{MR\_Score - \min(MR\_Score)}{\max(MR\_Score) - \min(MR\_Score)} \tag{6}$$

where:

- MR_Score: Raw Mendelian Randomization score.
- $\min(MR\_Score)$: Minimum value of the MR scores in the dataset.
- $\max(MR\_Score)$: Maximum value of the MR scores in the dataset.

Genes were classified into three categories based on their statistical significance and effect direction:

- **Risk genes:** Genes with a positive beta value, indicating an increased likelihood of the associated phenotype.
- **Preventive genes:** Genes with a negative beta value, suggesting a protective effect against the associated phenotype.
- **Non-significant genes:** Genes with a p-value greater than 0.05 or an FDR greater than 0.05, indicating no statistically significant association.

CT Score

CT analysis classified nodes based on two types:

- **Type I:** Critical (0), Redundant (1), or Ordinary (2)
- **Type II:** Critical (0), Redundant (1), or Ordinary (2)

Weight Calculation:

$$\text{Weight} = \begin{cases} 2 & \text{if TypeI} = 0 \text{ and TypeII} \neq 0, \\ 1 & \text{if TypeI} \neq 0 \text{ and TypeII} = 0, \\ 0 & \text{otherwise.} \end{cases} \tag{7}$$

where:

- TypeI: Classification of nodes based on their impact on driver nodes (e.g., critical, redundant, or ordinary).
- TypeII: Classification of nodes based on their presence in driver node sets (e.g., critical, redundant, or ordinary).

CT Score Computation:

$$CT\_Score = K \times \text{Weight} \tag{8}$$

where:

- CT_Score: Computed score representing the contribution of a node to network controllability.
- K: A constant or scaling factor determined by the analysis context.
- Weight: A classification-based multiplier determined by the node's type (e.g., critical, redundant, or ordinary).

CT Score Normalization:

$$CT\_Score_{norm} = \frac{CT\_Score - \min(CT\_Score)}{\max(CT\_Score) - \min(CT\_Score)} \tag{9}$$

where:

- CT_Score_norm: Normalized CT score, scaled to a range between 0 and 1.
- CT_Score: Raw CT score calculated for a node.
- min(CT_Score): Minimum CT score observed across all nodes in the analysis.
- max(CT_Score): Maximum CT score observed across all nodes in the analysis.

Final Score

The final score for each gene was computed as:

$$Final\_Score = \alpha \times MR\_Score_{norm} + (1 - \alpha) \times CT\_Score_{norm} \tag{10}$$

where:

- Final_Score: Combined score representing the integration of MR and CT analyses.
- α: Weighting parameter ($0 \leq \alpha \leq 1$) determining the relative contribution of MR and CT scores.

- MR_Score_norm: Normalized Mendelian Randomization score, scaled between 0 and 1.
- CT_Score_norm: Normalized Control Theory score, scaled between 0 and 1.

Alpha Value Exploration

Five different α values were evaluated:

- $\alpha = 1.00$: Pure MR score
- $\alpha = 0.75$: MR-weighted combination
- $\alpha = 0.50$: Balanced combination
- $\alpha = 0.25$: CT-weighted combination
- $\alpha = 0.00$: Pure CT score

Result Generation

Final output files included:

- Gene ranking based on Final Score
- Gene effect classification (Risk/Preventive/Non-significant)
- Critical gene classification (Type I/Type II/Not Critical)
- Normalized scores (MR and CT)

Conclusion

This scoring system provides a flexible framework for integrating MR and CT. The complete implementation code is available in Notebook 6.

Enrichment Analysis (EA)

Analysis Definition and Purpose

Enrichment Analysis (EA) was applied to determine whether gene sets are enriched for specific biological attributes compared to chance. The analysis follows a systematic process:

- Gene Set Selection: Disease-associated genes
- Background Set Definition: Complete genome reference
- Statistical Testing: Hypergeometric and Fisher's exact tests
- P-value Correction: Multiple testing adjustment
- Result Interpretation: Enriched annotation analysis

Analysis Implementation

We applied the following steps:

- Enrichment analysis
- Genome annotation
- Pathway analysis
- Visualization

Enrichment Categories

We ran an analysis across three ontologies:

- Biological Process (BP)
- Molecular Function (MF)
- Cellular Component (CC)

And three different pathways:

- KEGG pathways
- Reactome pathways
- WikiPathways

Core Gene Analysis

Processing of 21 core genes:

- GO term enrichment calculation
- KEGG pathway mapping
- Reactome pathway analysis

Extended Analysis

Analysis of 32 core genes:

- Comprehensive pathway mapping
- Cross-database enrichment
- Functional annotation clustering

Statistical Processing

Statistical settings include:

- p-value adjustment: Benjamini-Hochberg
- q-value threshold: 0.05
- Alpha threshold: 0.5 for sensitivity analysis

Conclusion

The EA provided comprehensive pathway insights for Long COVID genetic factors. Complete implementation code is available in Notebook 3.

Gene Expression Clustering

The gene expression data was processed through the following steps:

1. Subsetting data for causal genes
2. Removing metadata columns
3. Cleaning column names
4. Transposing data matrix
5. Aggregating by Subject-ID using median values

The analysis explored multiple parameter combinations:

- **Cluster Number (clusterNum $\in [2, 5]$):** This parameter defines the range of cluster numbers to evaluate during clustering analysis. The values between 2 and 5 specify the minimum and maximum number of clusters to test, helping identify the optimal number of clusters that best represent the data.
- **Maximum Number of Clusters (maxK $\in [5, 7]$):** The maximum number of clusters to consider when performing clustering algorithms. Values in this range allow flexibility while ensuring computational efficiency by not testing excessively high numbers of clusters.
- **Proportion of Items (pItem $\in [0.5, 0.9]$):** This parameter represents the proportion of items (samples) to be resampled during each iteration of the clustering process. Resampling between 50% and 90% of the data ensures robust cluster identification while maintaining computational efficiency.
- **Proportion of Features (pFeature $\in [0.5, 1.0]$):** Indicates the proportion of features (variables or genes) to include in each iteration of clustering. Testing values between 50% and 100% ensures that enough features are considered to identify meaningful patterns while avoiding overfitting.

Two clustering algorithms were implemented:

1. **Hierarchical clustering (hc):** This clustering method builds a hierarchy of clusters by iteratively merging or splitting them based on their similarity. In this study, hierarchical clustering helps visualize relationships between data points by creating a dendrogram, where closer branches represent higher similarity.
2. **Partitioning Around Medoids (pam):** PAM is a robust clustering method that partitions the data into a pre-specified number of clusters by selecting representative data points (medoids) as cluster centers. Unlike k-means, PAM minimizes the dissimilarity between points and their assigned medoids, making it less sensitive to noise and outliers.

The **ExecuteCC** function was implemented with error handling and data validation:

1. Data preprocessing and scaling
2. Consensus clustering execution
3. Distance matrix computation
4. Result extraction and validation

Clinical Data Analysis

Clinical data was processed to analyze symptom patterns:

1. Preprocess symptoms function implementation
2. Symptom frequency calculation
3. Statistical testing for significance

Statistical tests were performed:

- χ^2 **Test for Large Samples:** The chi-squared test is a statistical method used to assess whether there is a significant association between two categorical variables in large sample sizes. It compares the observed frequencies in a contingency table to the expected frequencies under the assumption of independence. This test is suitable when all expected cell frequencies are greater than or equal to 5, ensuring the validity of the test results.
- **Fisher's Exact Test for Small Samples ($n < 5$):** Fisher's Exact Test is a precise statistical method used for small sample sizes, specifically when the expected frequencies in a contingency table are less than 5. Unlike the chi-squared test, Fisher's Exact Test does not rely on large-sample approximations and calculates the exact probability of the observed data under the null hypothesis, making it ideal for sparse or small datasets.

Two types of heatmaps were generated:

1. Hierarchical clustering (hc)
2. Partitioning Around Medoids (pam)

Output Generation

Multiple output files were created:

1. Cluster assignments
2. Statistical test results
3. Frequency tables
4. Visualization outputs

Conclusion

The implemented analysis pipeline represents a comprehensive approach to analyzing Long COVID gene expression data. The methodology follows a systematic workflow:

1. Initial data preparation and quality control
2. Robust consensus clustering with parameter optimization
3. Clinical correlation analysis
4. Statistical validation
5. Visualization of results

Implementing both hierarchical and PAM clustering algorithms provided complementary approaches to pattern discovery. Integrating clinical data with gene expression profiles enabled a multi-dimensional understanding of Long COVID manifestations.

References

[1] Lammi, V. *et al.* Genome-wide association study of long covid. *Nature Genetics* **57**, 1402–1417 (2025). Epub 2025 May 21.

[2] GTEx portal - datasets (2023). URL https://gtexportal.org/home/datasets. Accessed 8 Sep 2023.

[3] NCBI GEO - GSE215865 (2023). URL https://www.ncbi.nlm.nih.gov/geo/query/acc.cgi?acc=GSE215865. Accessed 11 Feb 2023.
