# Supplementary figures and images for: Integrative multi-omics framework for causal gene discovery in Long COVID
Source: PLoS Comput Biol. 2025 Dec 1;21(12):e1013725. doi: 10.1371/journal.pcbi.1013725 (PMC12677781; doi:10.1371/journal.pcbi.1013725)

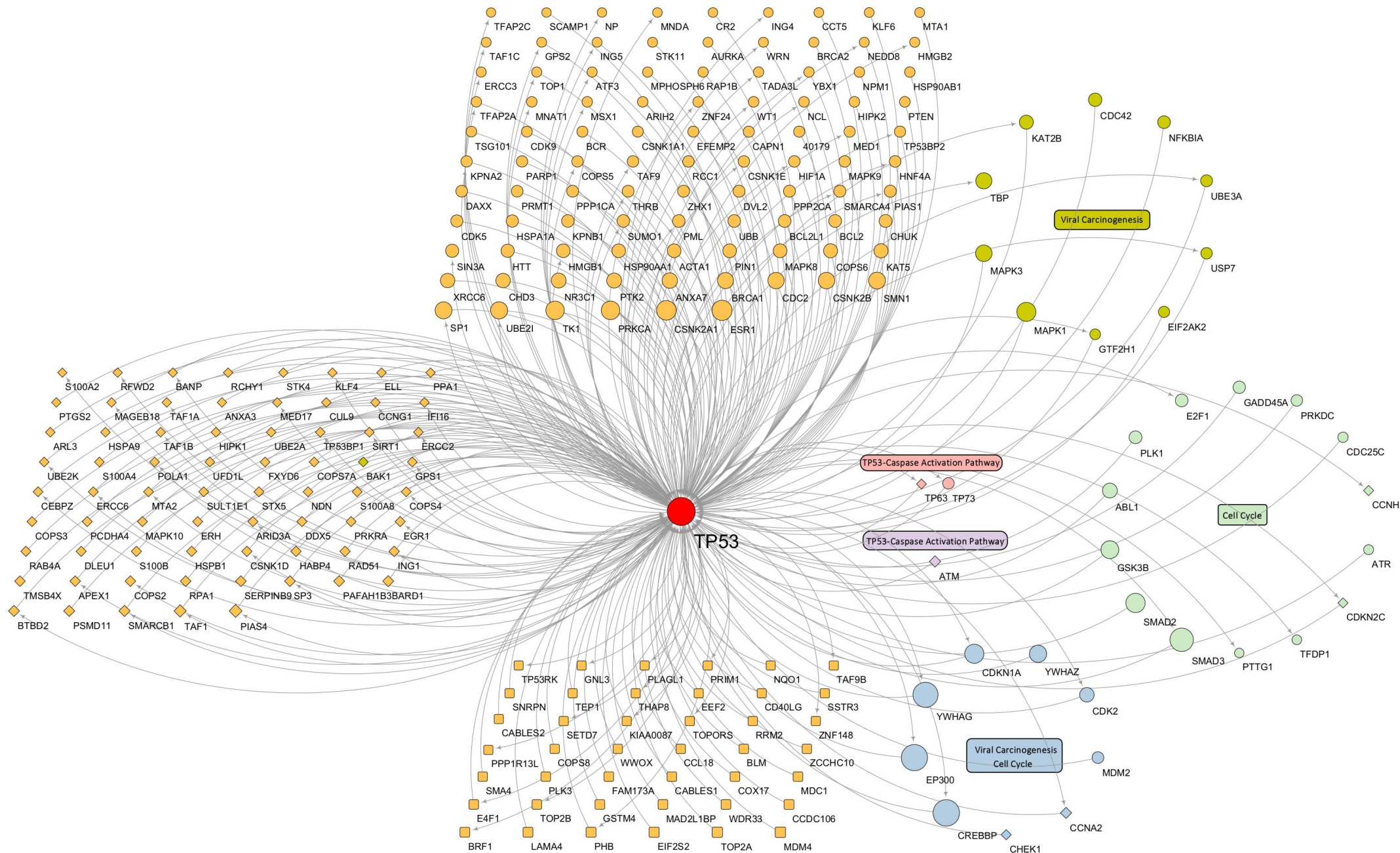

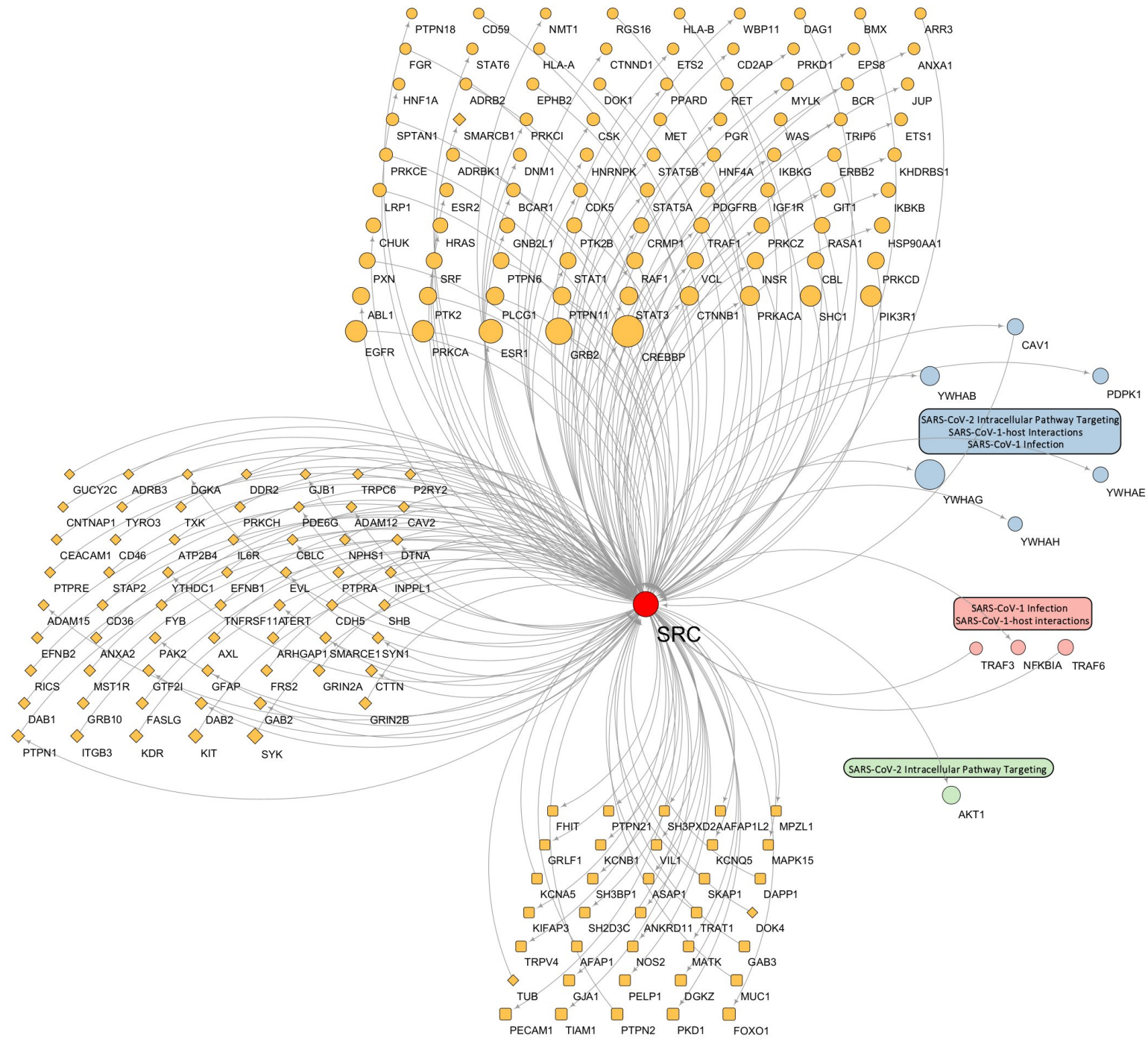

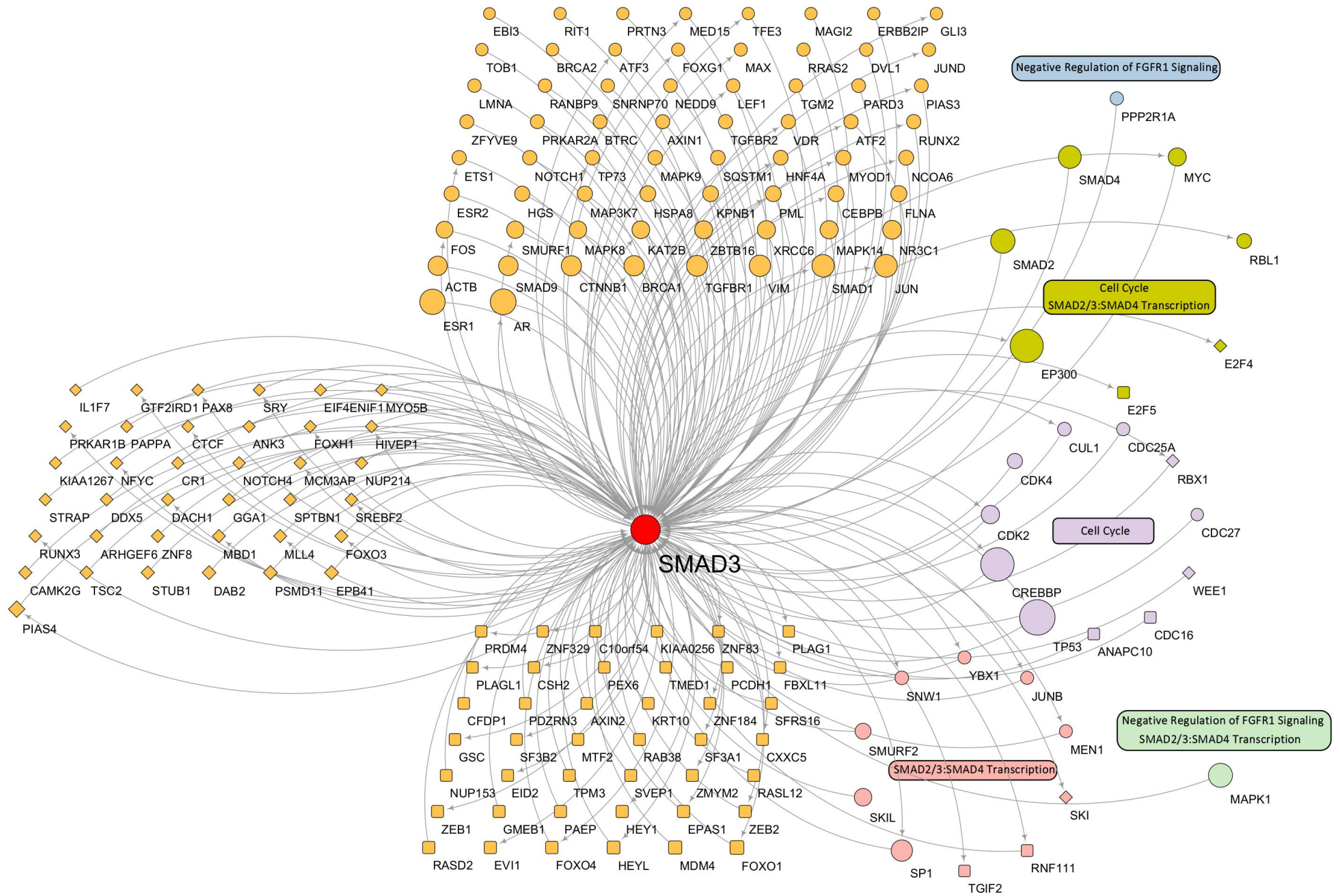

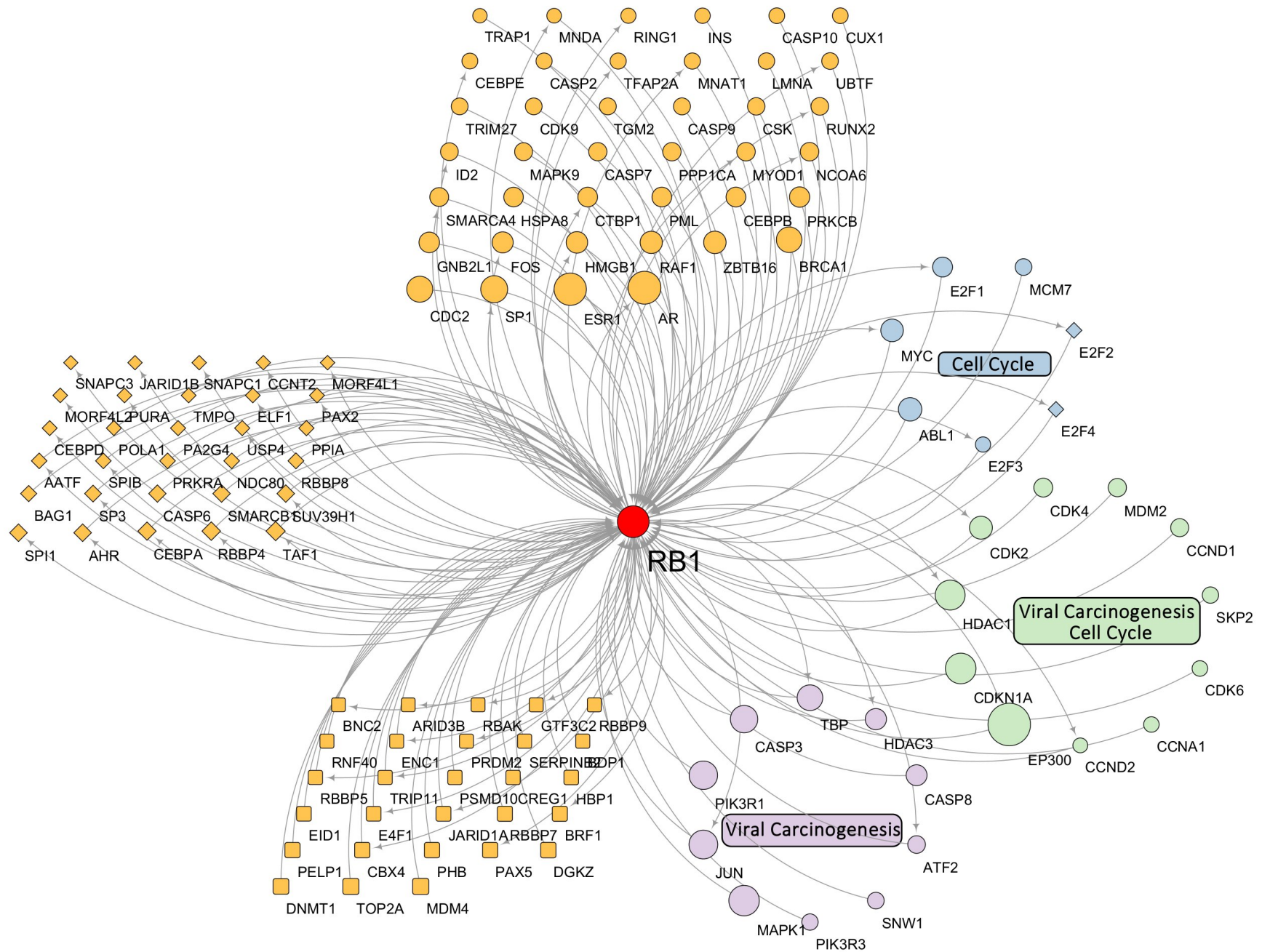

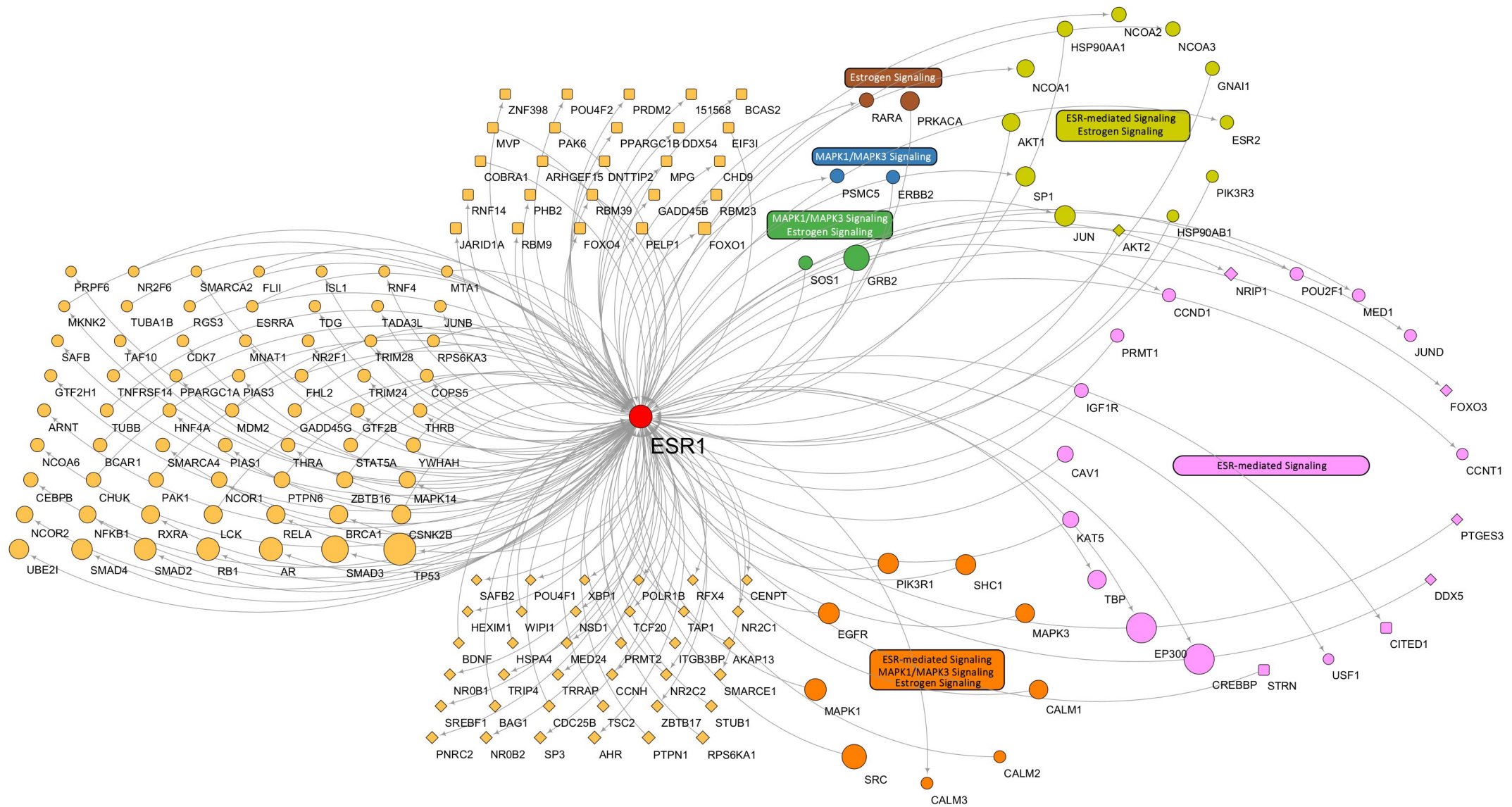

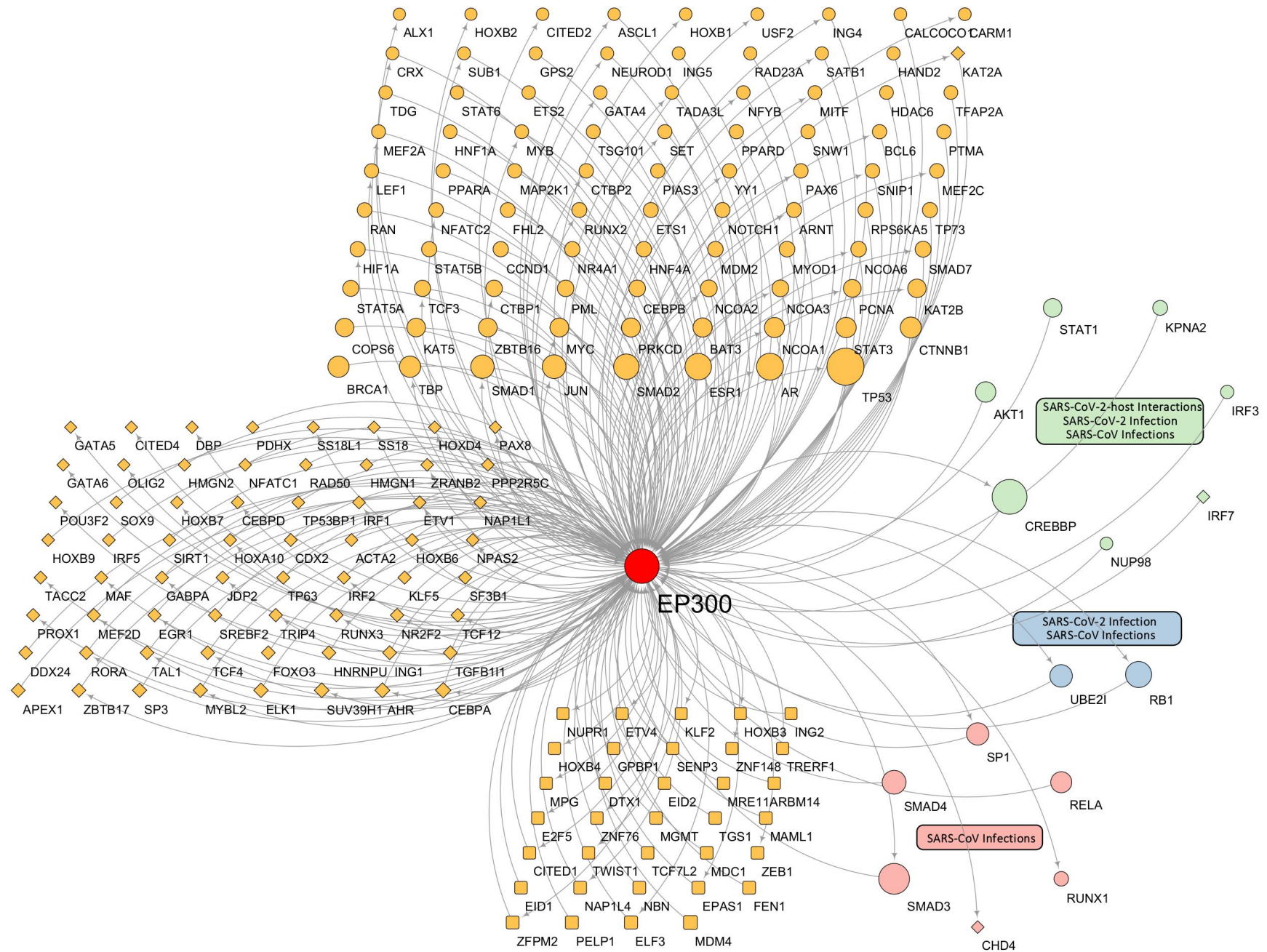

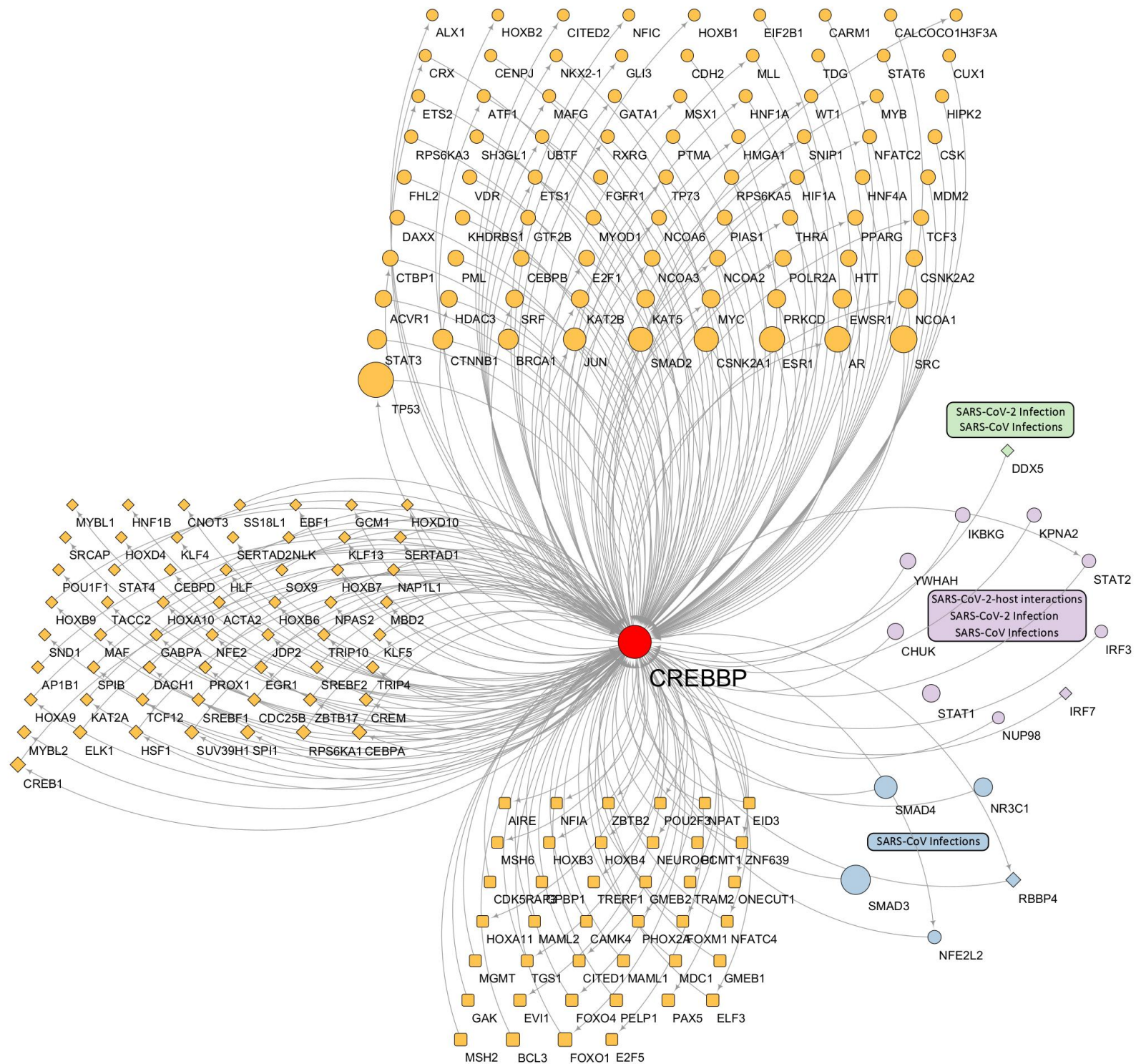

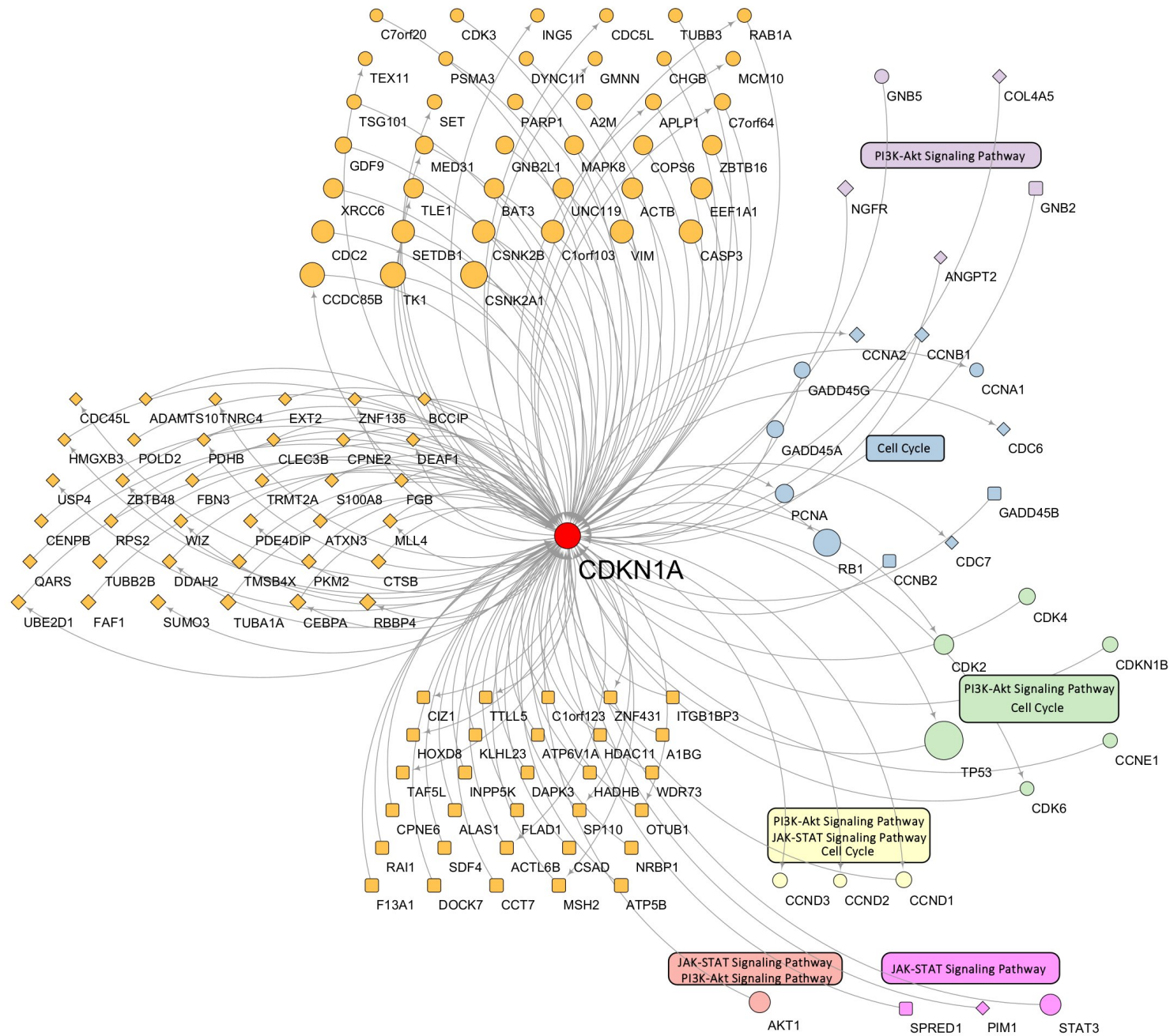

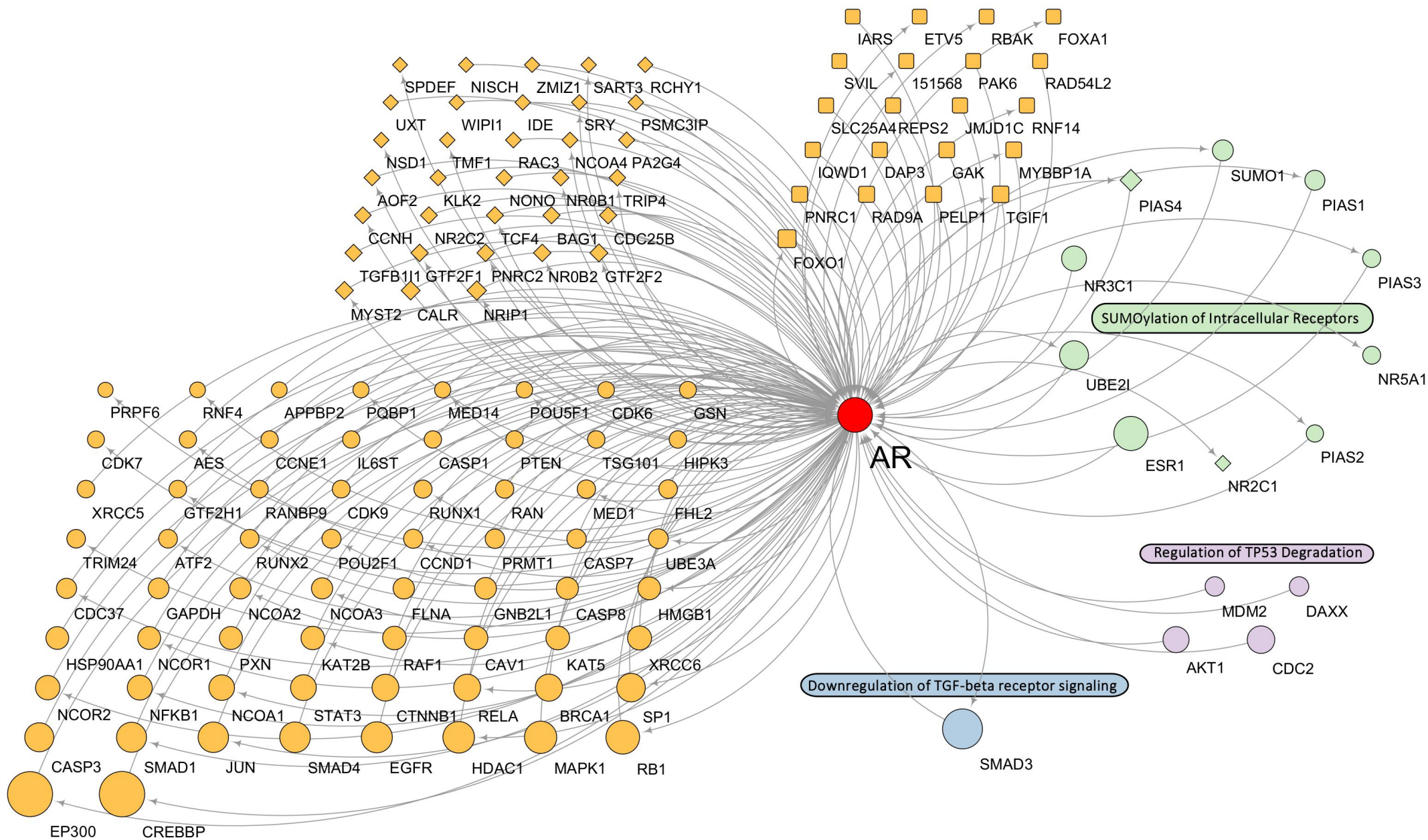

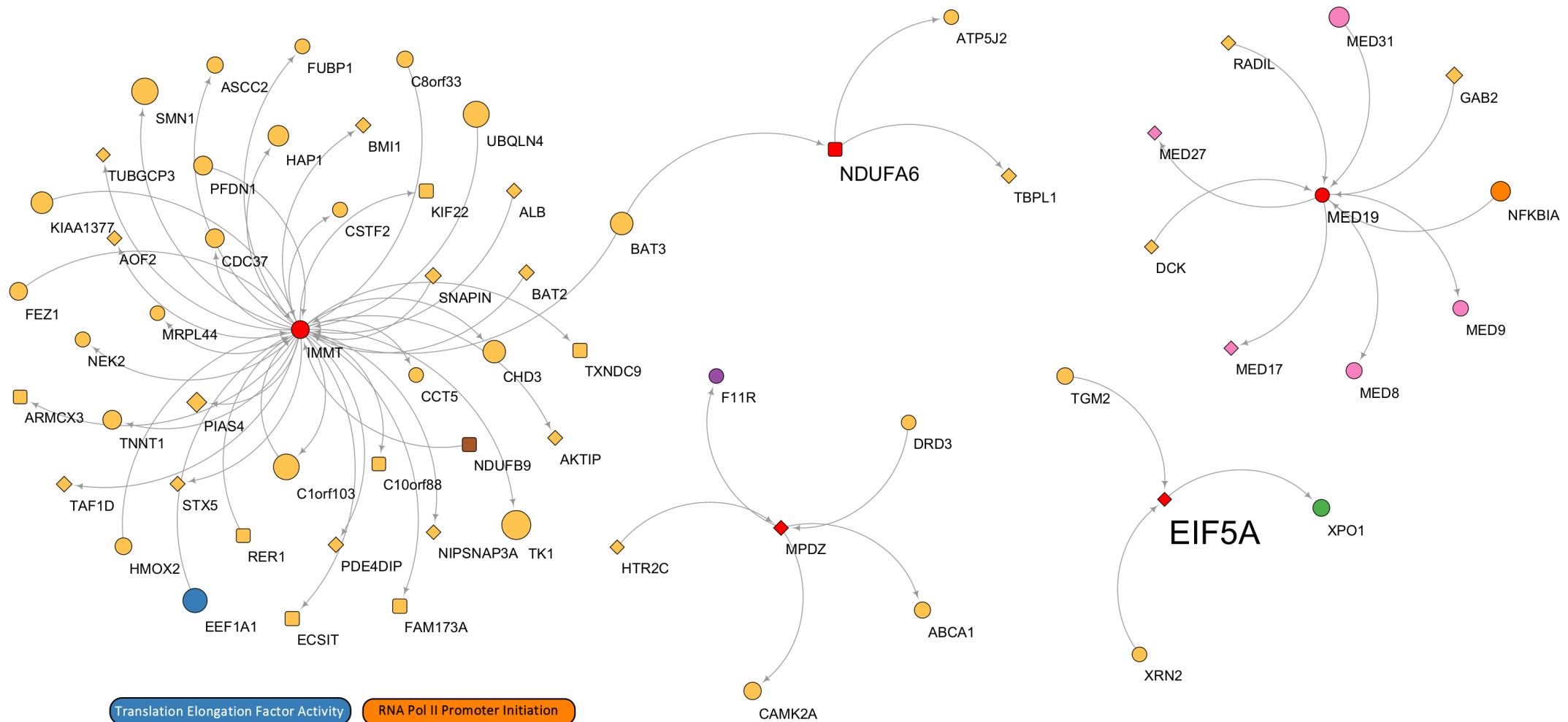

Supplement: S1 Fig — (PDF) [file pcbi.1013725.s015.pdf]
