# Supplementary material for: Integrative multi-omics framework for causal gene discovery in Long COVID
Source: PLoS Comput Biol. 2025 Dec 1;21(12):e1013725. doi: 10.1371/journal.pcbi.1013725 (PMC12677781; doi:10.1371/journal.pcbi.1013725)
Supplement: S2 Fig — Individual patient-level heatmap showing expression patterns of the 32 candidate Long COVID genes across all samples. Color gradient represents z-scored log2 expression values with hierarchical clustering of both genes and samples. (PDF) [file pcbi.1013725.s016.pdf]

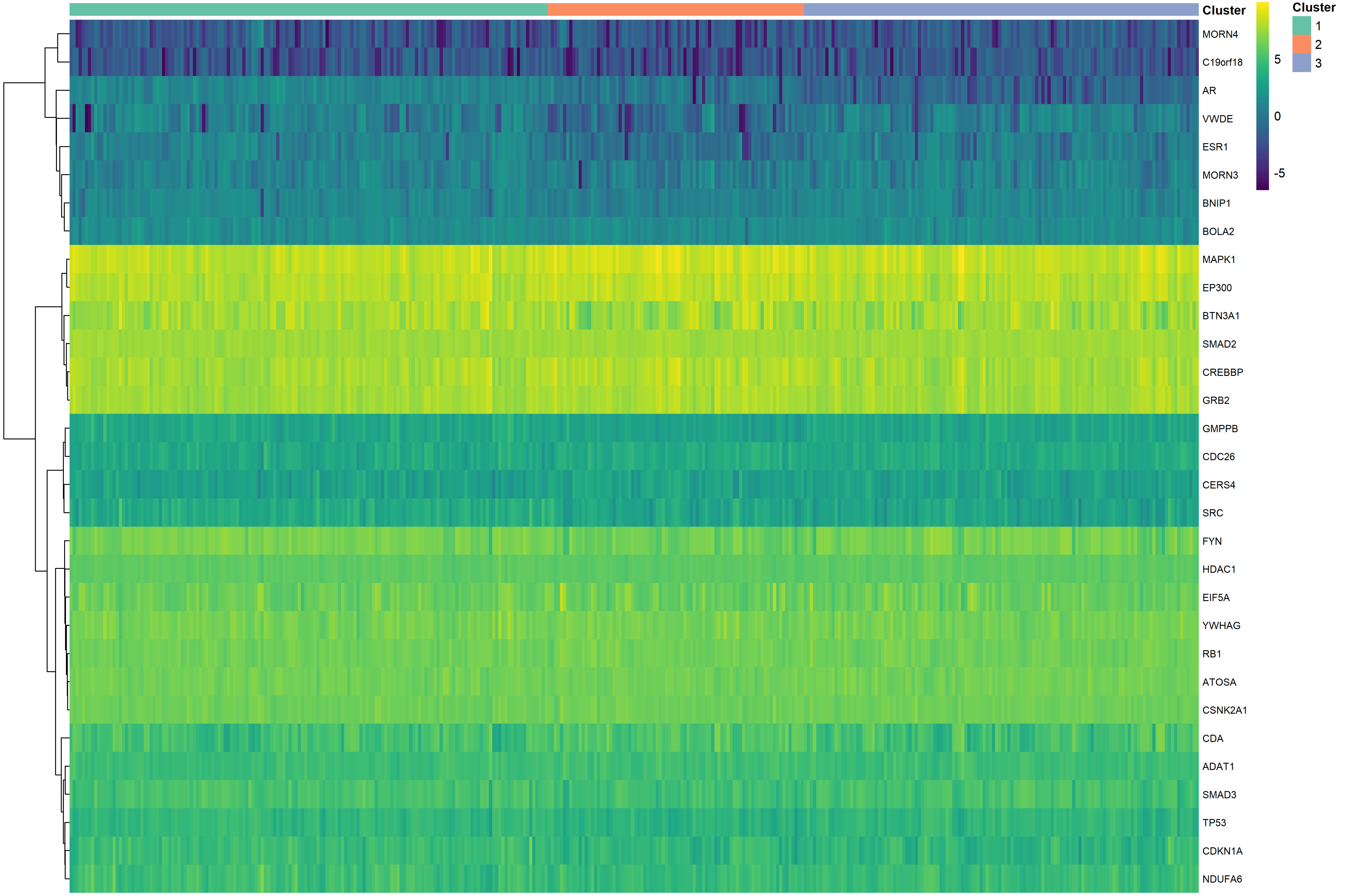
Cluster
MORN4
C19orf18
AR
VWDE
ESR1
MORN3
BNIP1
BOLA2
MAPK1
EP300
BTN3A1
SMAD2
CREBBP
GRB2
GMPPB
CDC26
CERS4
SRC
FYN
HDAC1
EIF5A
YWHAG
RB1
ATOSA
CSNK2A1
CDA
ADAT1
SMAD3
TP53
CDKN1A
NDUFA6
5
0
-5
Cluster
1
2
3

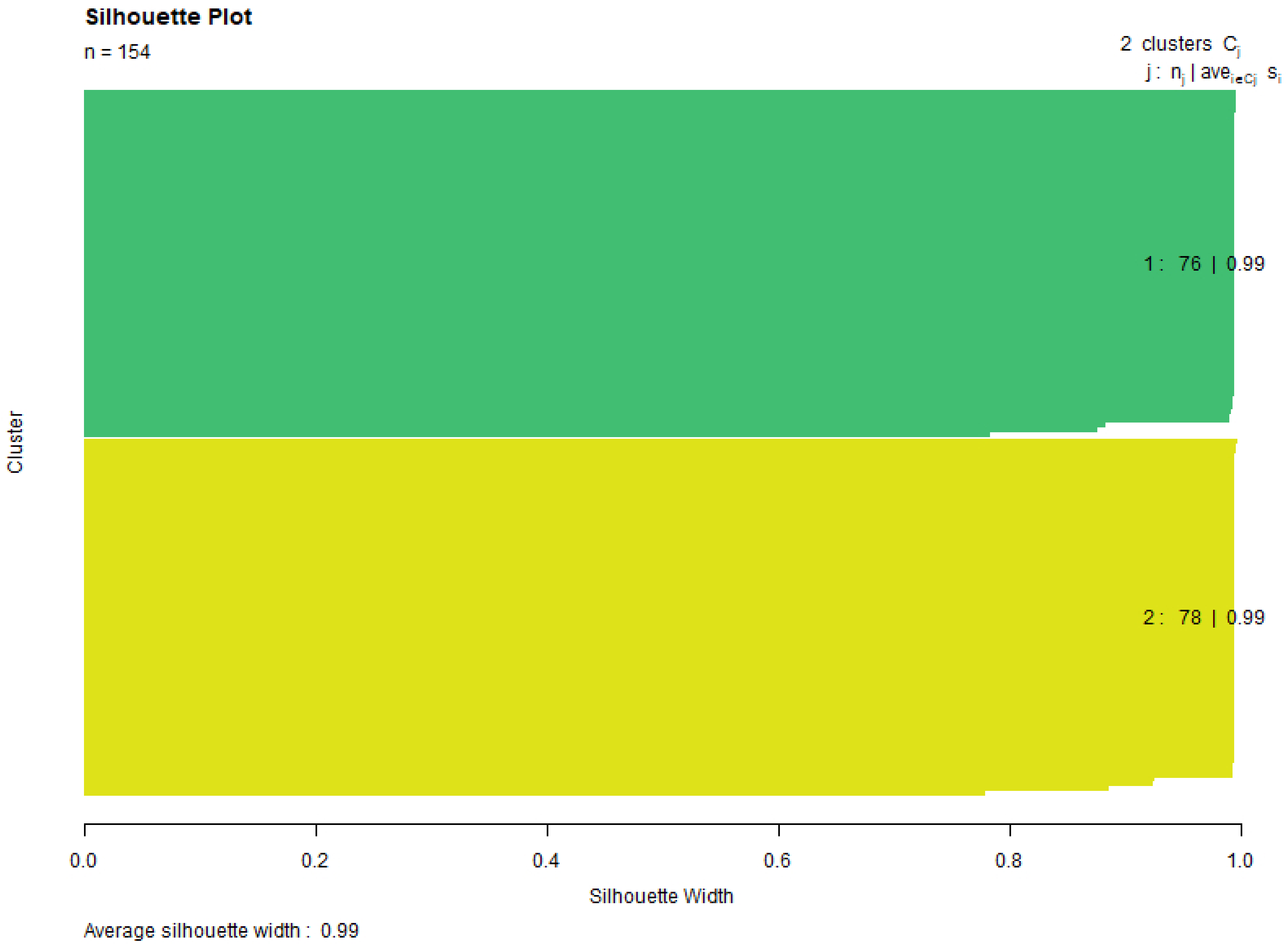
Silhouette Plot
n = 154
2 clusters C_j
j : n_j | $ave_{i \in Cj}$ s_i
1 : 76 | 0.99
2 : 78 | 0.99
Cluster
0.0
0.2
0.4
0.6
0.8
1.0
Silhouette Width
Average silhouette width : 0.99

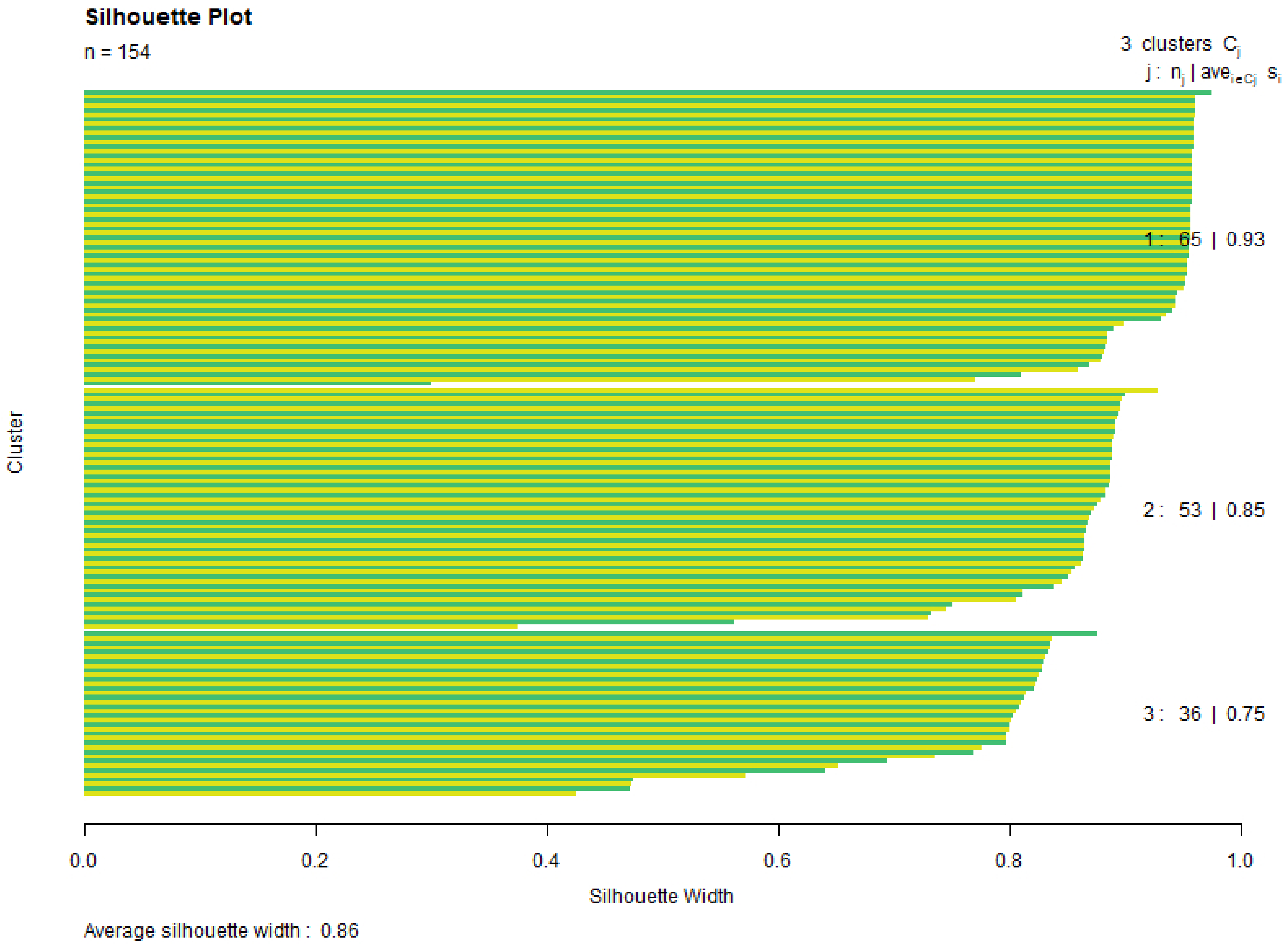

Silhouette Plot
n = 154
3 clusters C_j
j : n_j | $ave_{i \in Cj}$ s_i
1 : 65 | 0.93
2 : 53 | 0.85
3 : 36 | 0.75
Cluster
0.0
0.2
0.4
0.6
0.8
1.0
Silhouette Width
Average silhouette width : 0.86

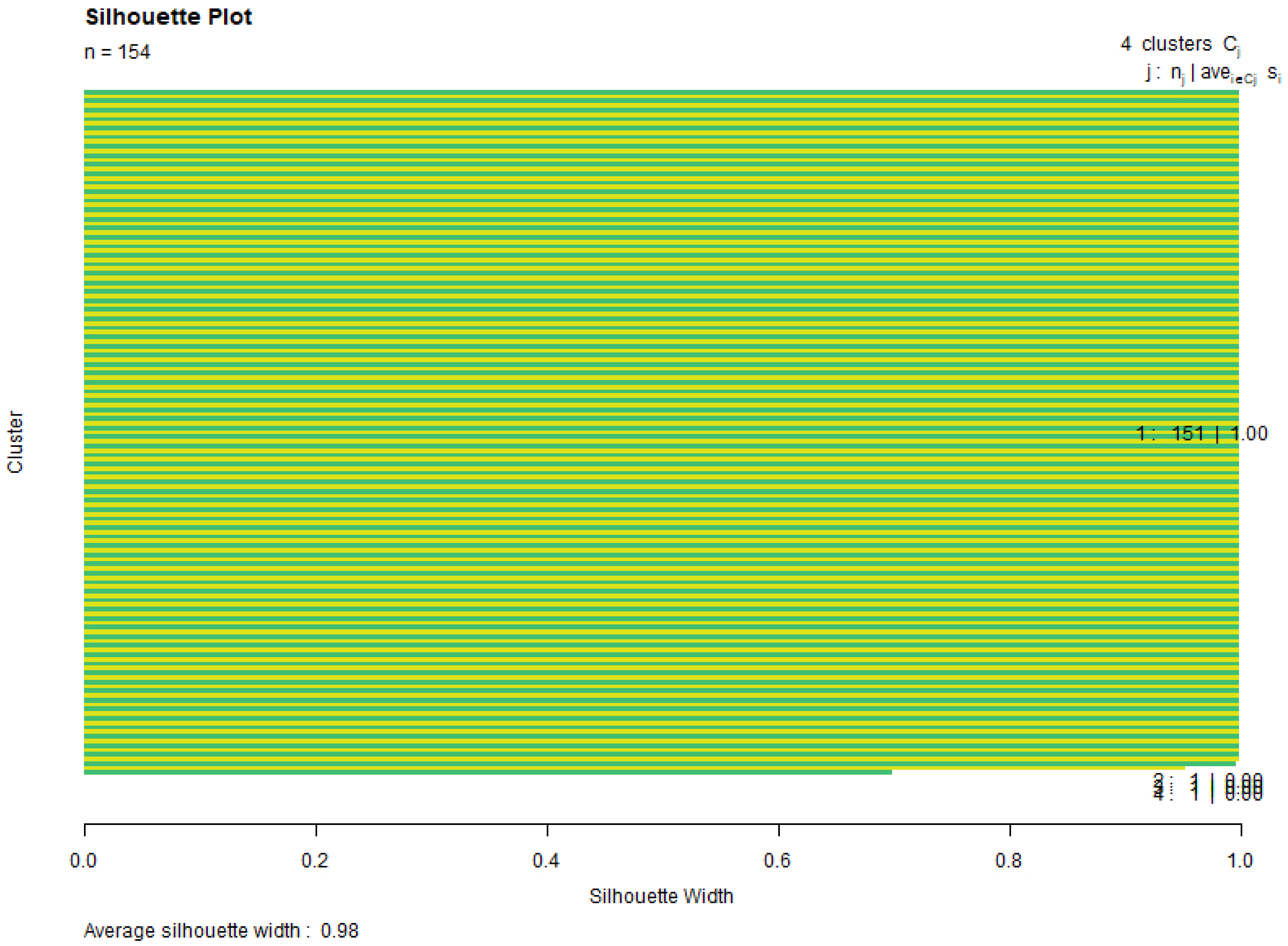
Silhouette Plot
n = 154
4 clusters C_j
j : n_j | $ave_{i \in Cj}$ s_i
1 : 151 | 1.00
Cluster
0.0
0.2
0.4
0.6
0.8
1.0
Silhouette Width
Average silhouette width : 0.98

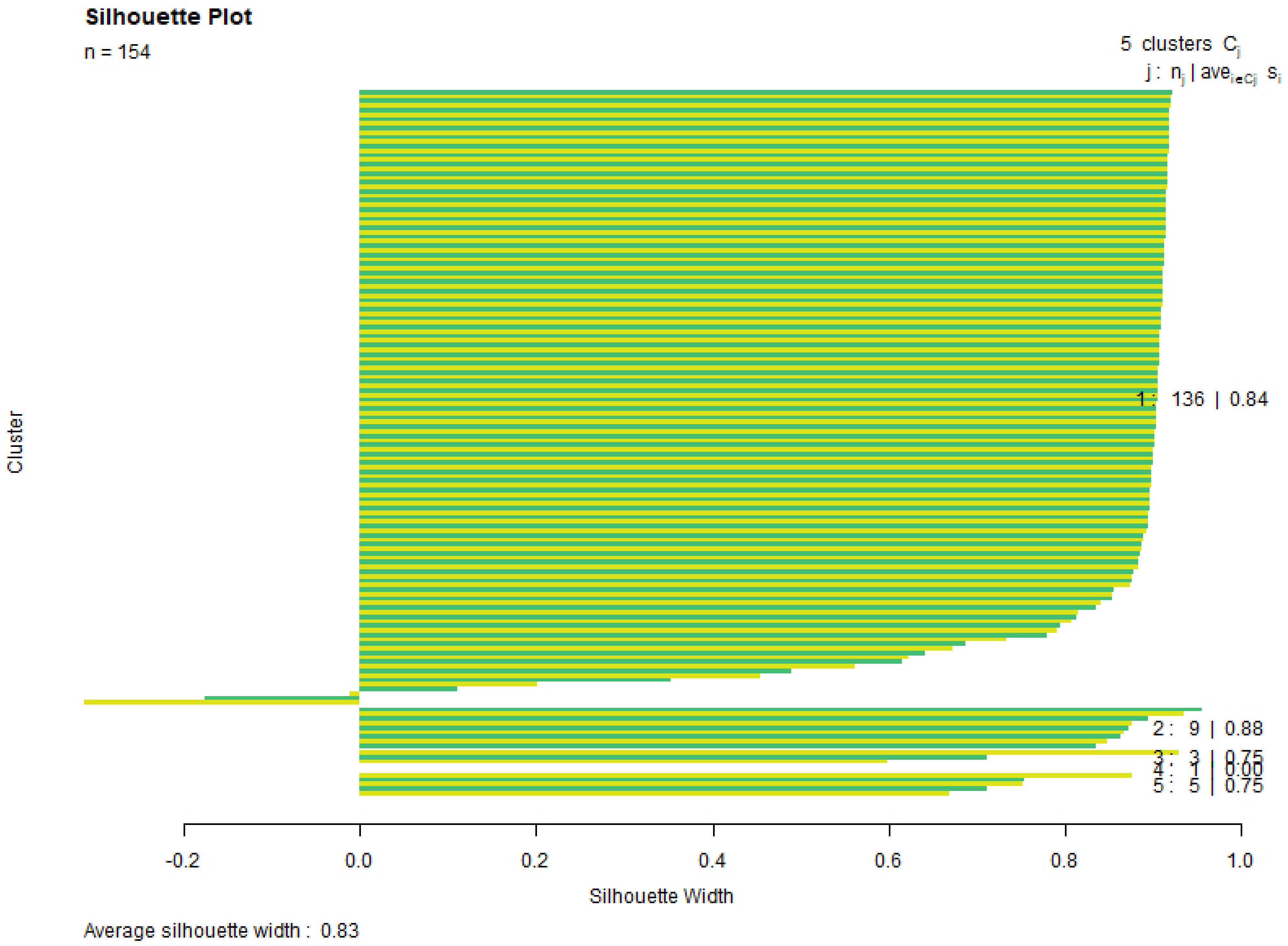

Silhouette Plot
n = 154
5 clusters C_j
j : n_j | $ave_{i \in Cj}$ s_i
1 : 136 | 0.84
2 : 9 | 0.88
3 : 3 | 0.75
4 : 1 | 0.00
5 : 5 | 0.75
Cluster
-0.2
0.0
0.2
0.4
0.6
0.8
1.0
Silhouette Width
Average silhouette width : 0.83
