# Supplementary material for: Integrative multi-omics framework for causal gene discovery in Long COVID
Source: PLoS Comput Biol. 2025 Dec 1;21(12):e1013725. doi: 10.1371/journal.pcbi.1013725 (PMC12677781; doi:10.1371/journal.pcbi.1013725)
Supplement: S3 Fig — Comparative visualization of gene rankings and controllability scores across multiple PPI networks, demonstrating framework robustness and network-specific differences in topology and interaction coverage. (PDF) [file pcbi.1013725.s017.pdf]

Ven Diagrams PPIs

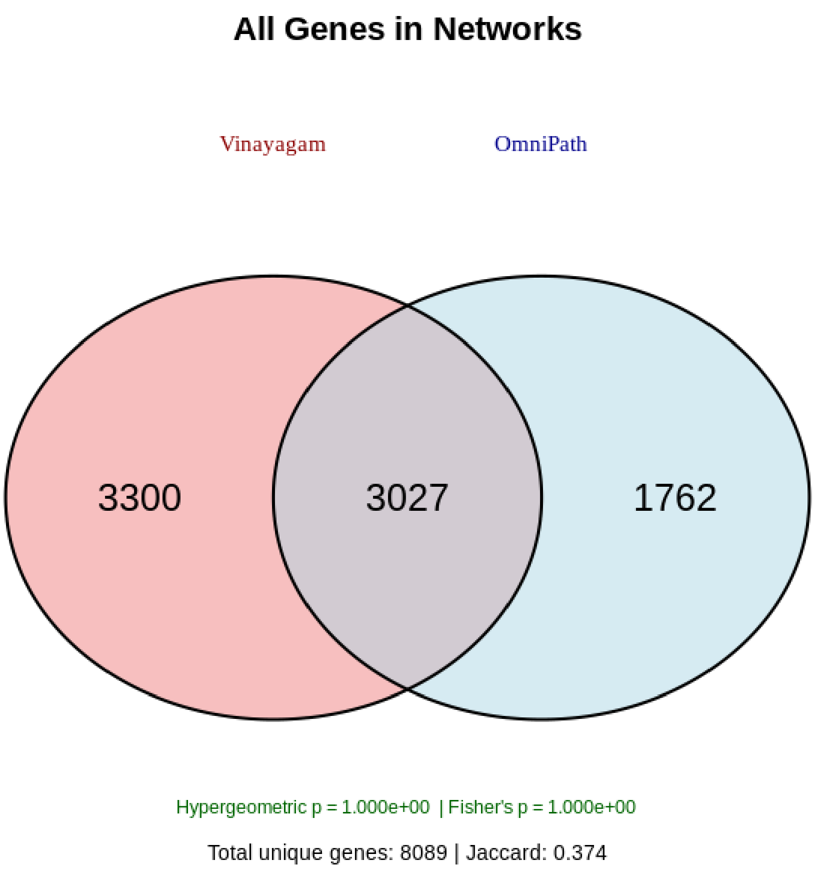

Critical Genes (Type I or II)

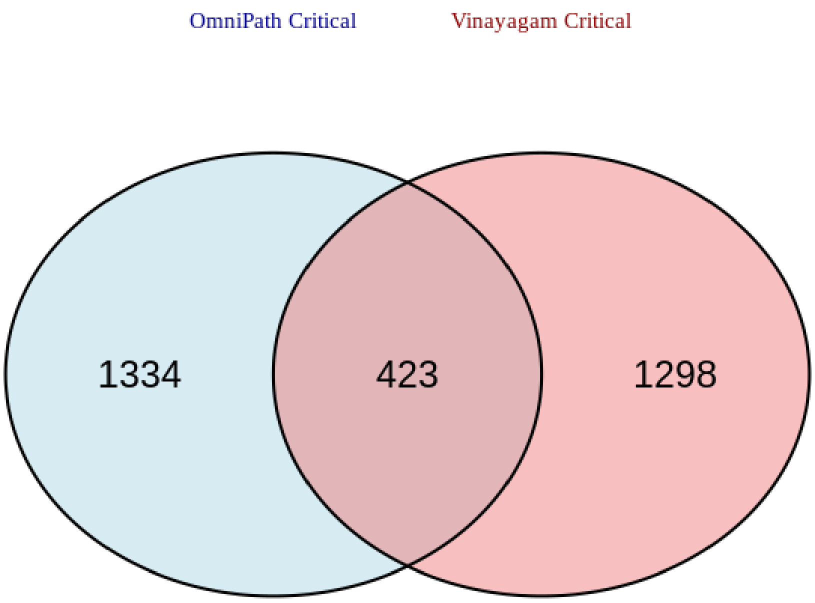

Hypergeometric p = 7.445e-04 * | Fisher's p = 7.445e-04 *

Total unique genes: 3055 | Jaccard: 0.138

Non-Critical Genes

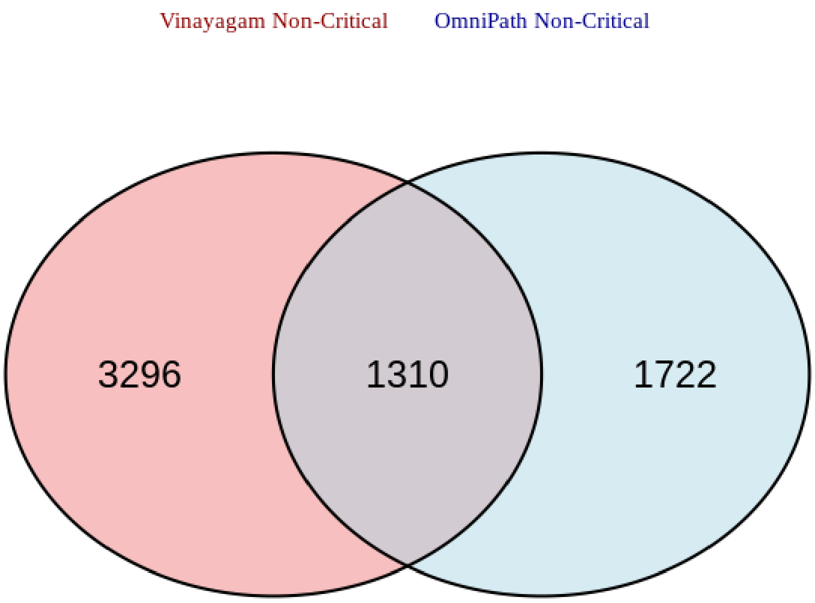

Hypergeometric p = 1.000e+00 | Fisher's p = 1.000e+00

Total unique genes: 6328 | Jaccard: 0.207

Top 16 Genes by Degree (K)

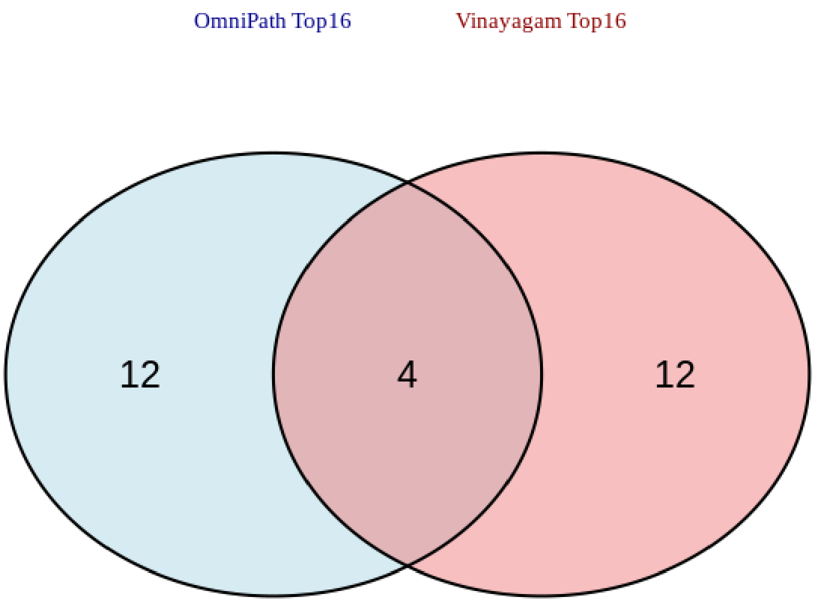

Hypergeometric p = 1.832e-08 * | Fisher's p = 1.832e-08 *

Total unique genes: 28 | Jaccard: 0.143

Critical Genes in Top 16 by Degree

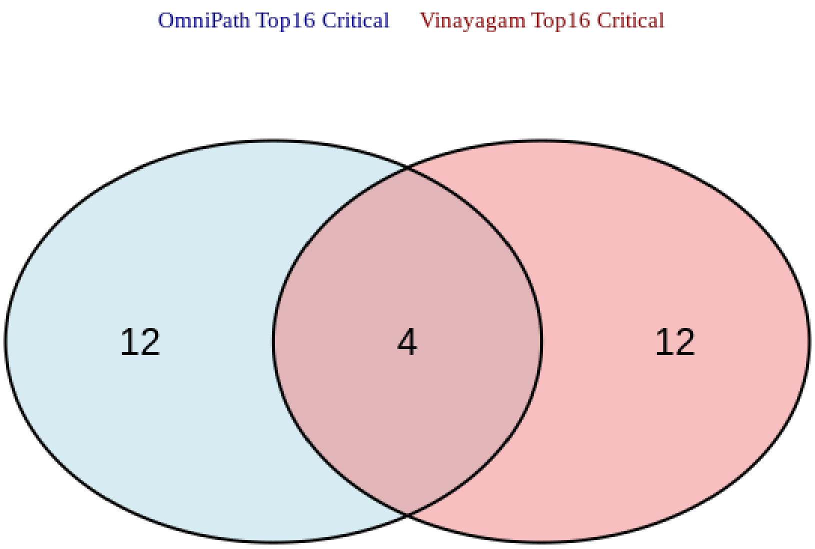

Total unique genes: 28

Top 100 Genes by Degree (K)

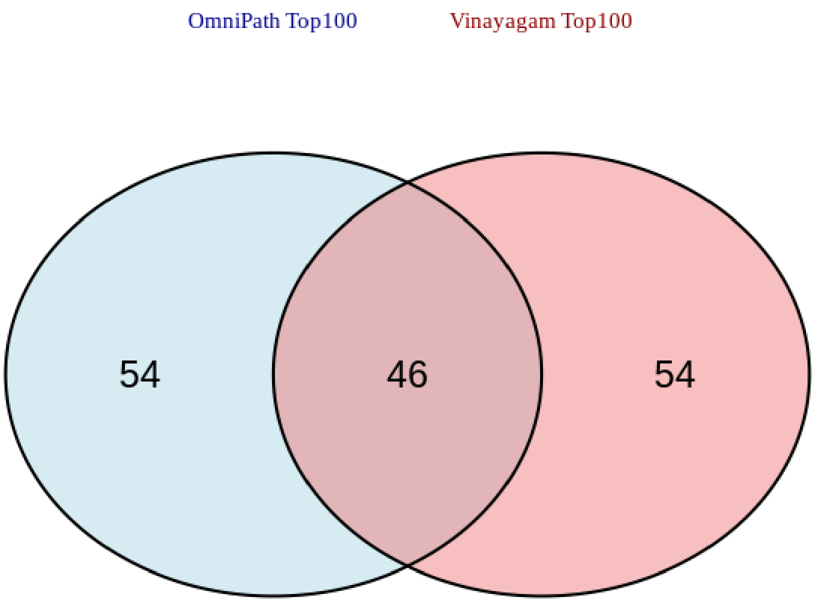

Hypergeometric p = 4.076e-65 * | Fisher's p = 4.076e-65 *

Total unique genes: 154 | Jaccard: 0.299

Critical Genes in Top 100 by Degree

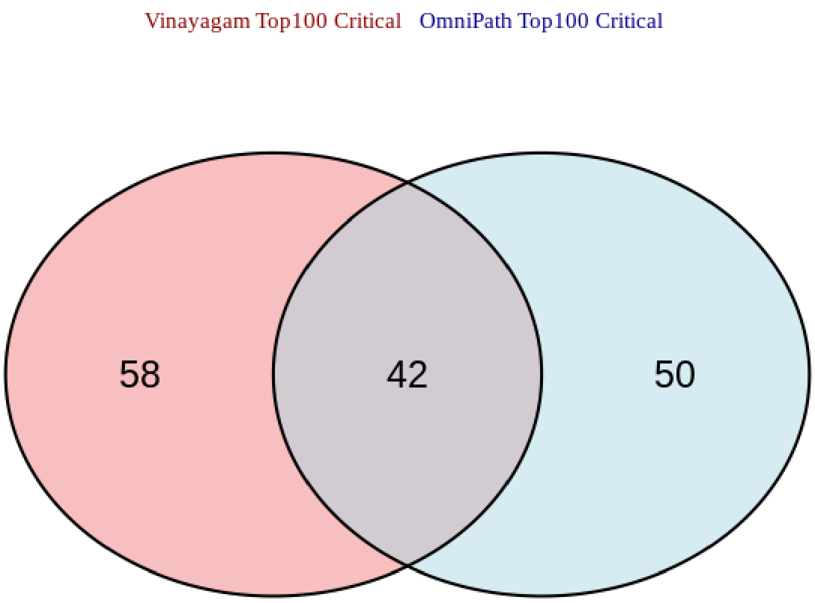

Hypergeometric p = 6.664e-59 * | Fisher's p = 6.664e-59 *

Total unique genes: 150 | Jaccard: 0.280

Non-Critical Genes in Top 100 by Degree

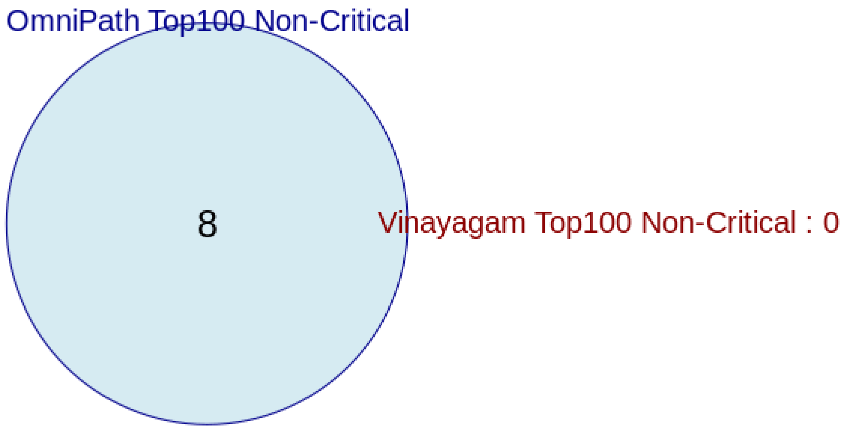

Type I Critical Genes

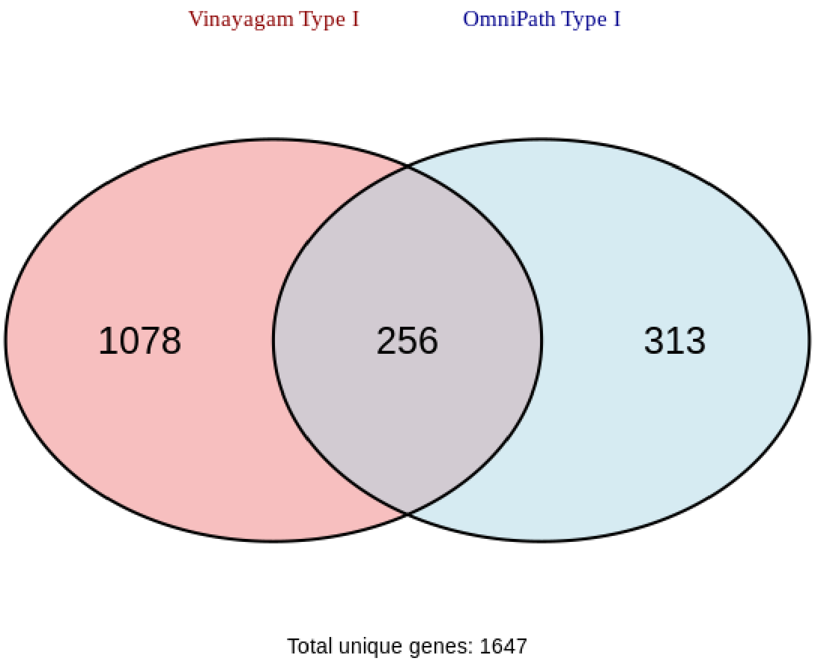

Type II Critical Genes

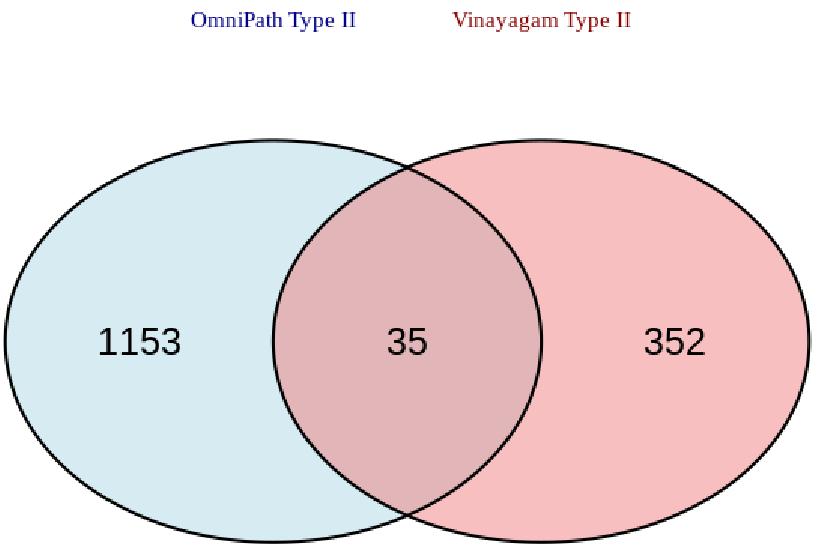

Total unique genes: 1540
