# Supplementary material for: Integrative multi-omics framework for causal gene discovery in Long COVID
Source: PLoS Comput Biol. 2025 Dec 1;21(12):e1013725. doi: 10.1371/journal.pcbi.1013725 (PMC12677781; doi:10.1371/journal.pcbi.1013725)
Supplement: S4 Fig — Visualization of the consistency of gene ranking across different normalization approaches and parameter settings (α), spanning from statistical causal evidence to network-based prioritization. (PDF) [file pcbi.1013725.s018.pdf]

Different Normalization Techniques for the TOP 16 Genes:

Top-16 genes across α — asinh

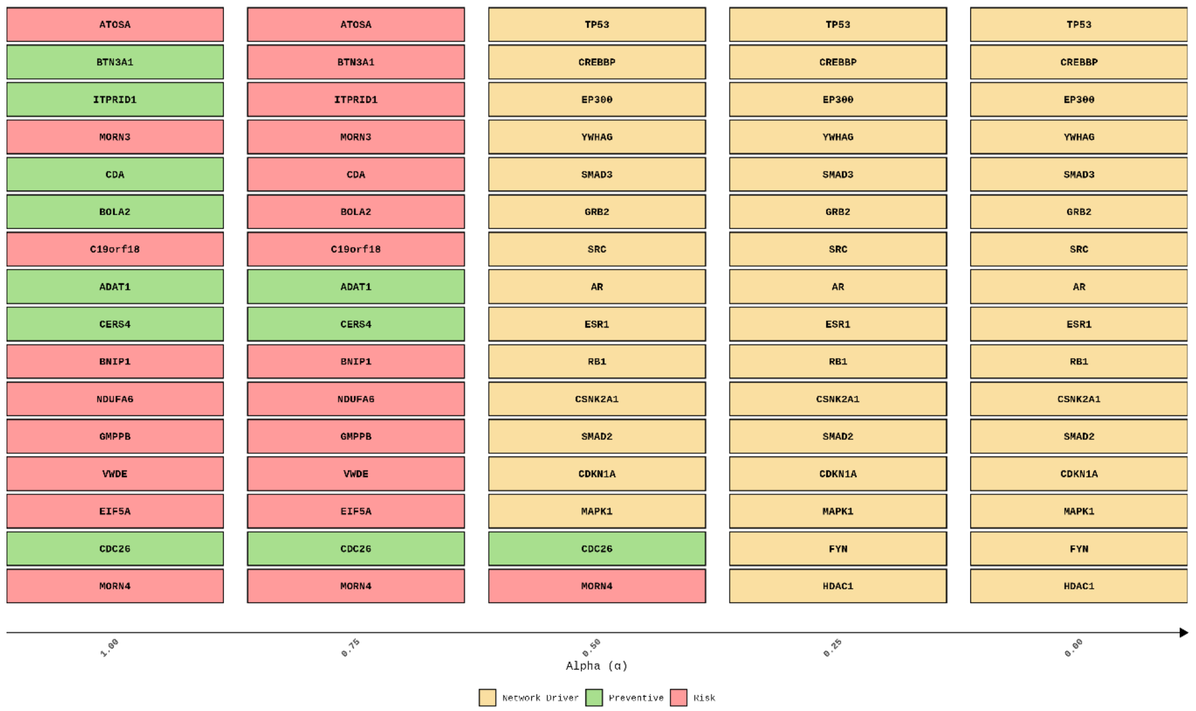

Top-16 genes across α — boxcox

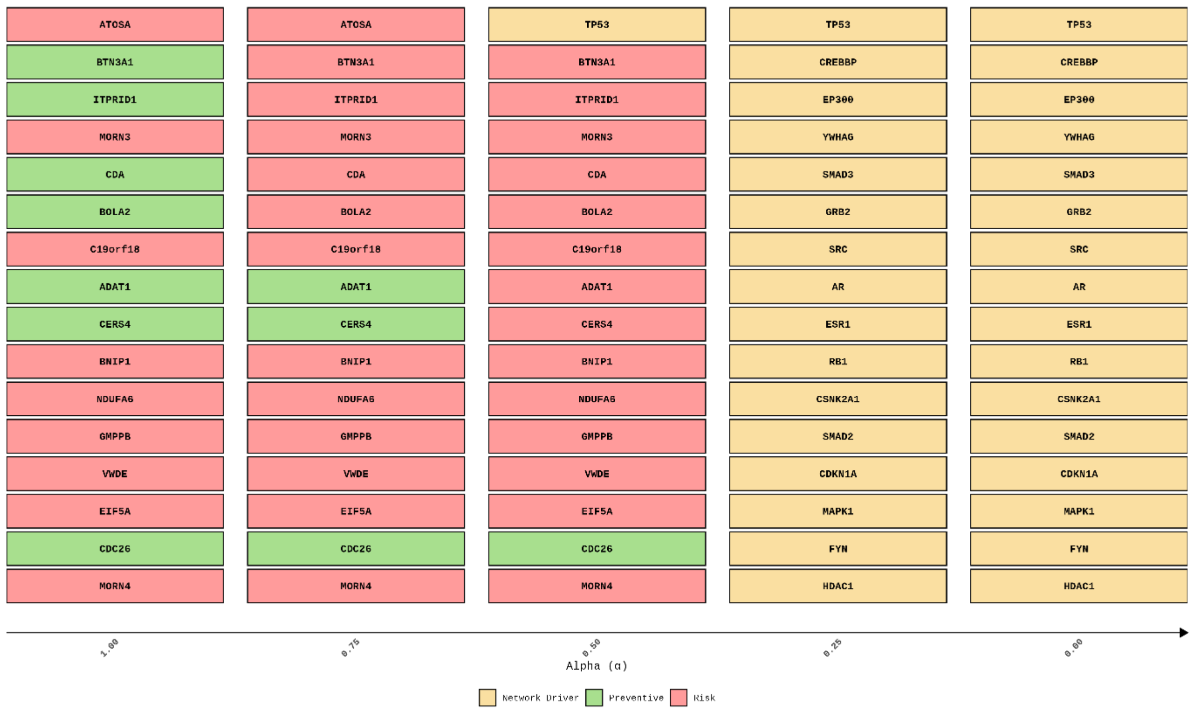

Top-16 genes across α — minmax

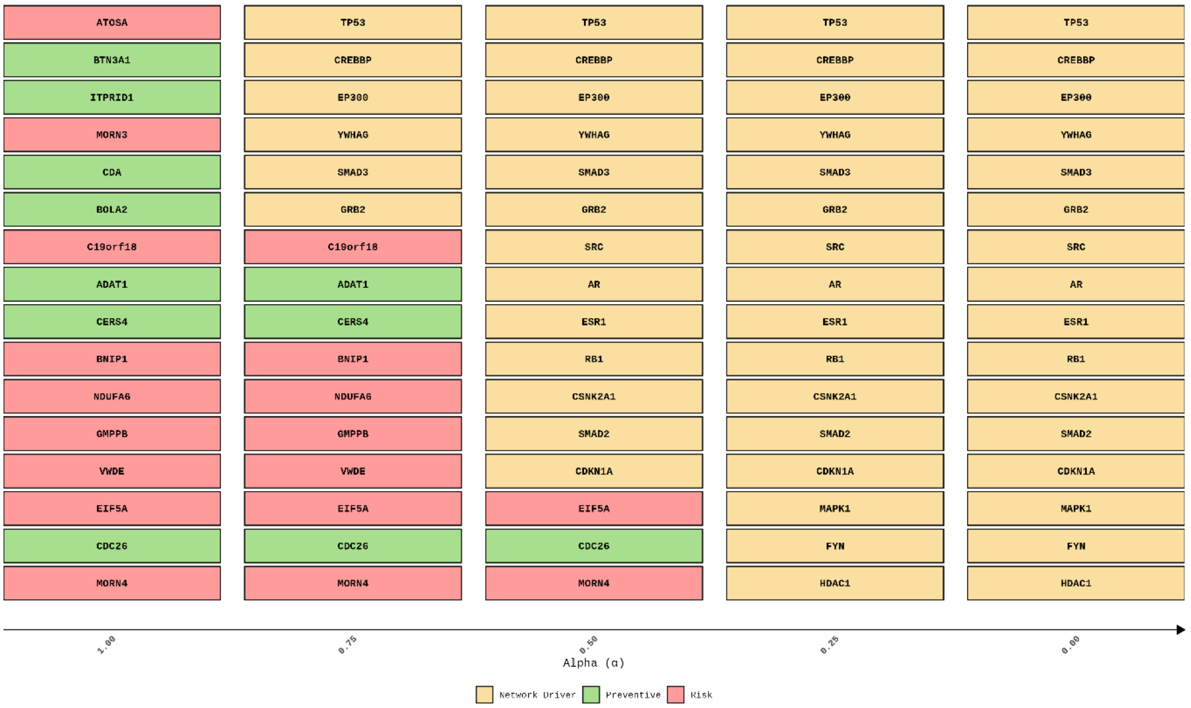

Top-16 genes across α — quantile

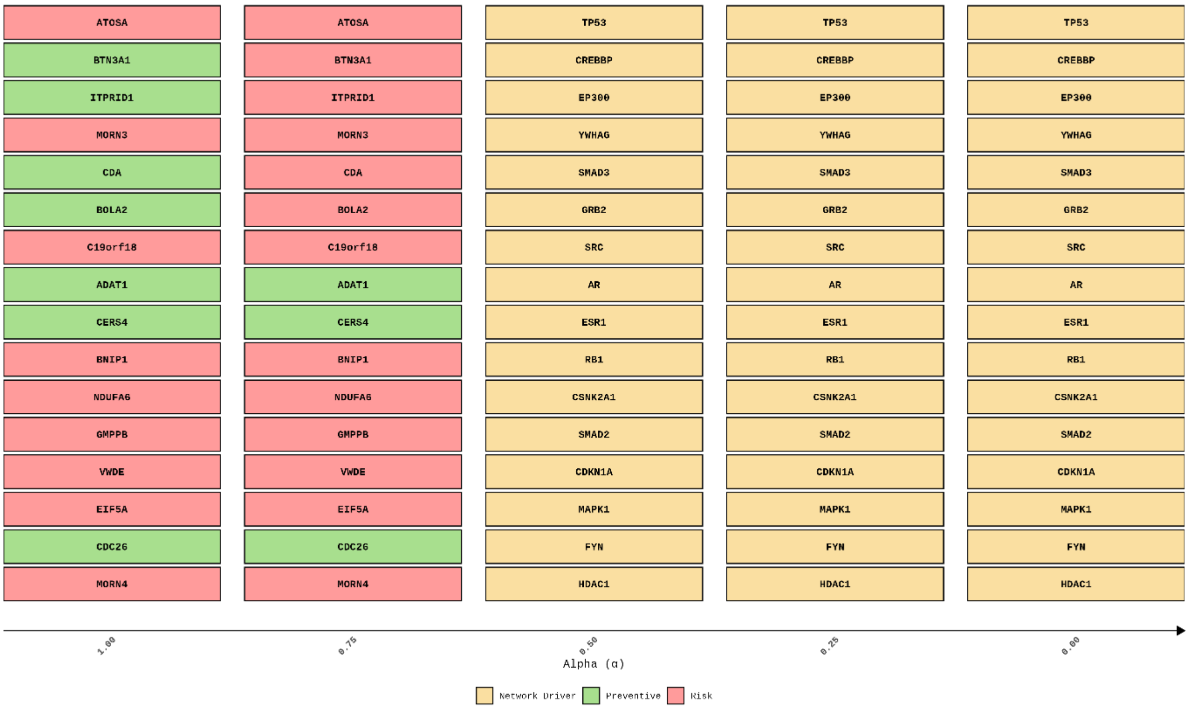

Top-16 genes across α — rank

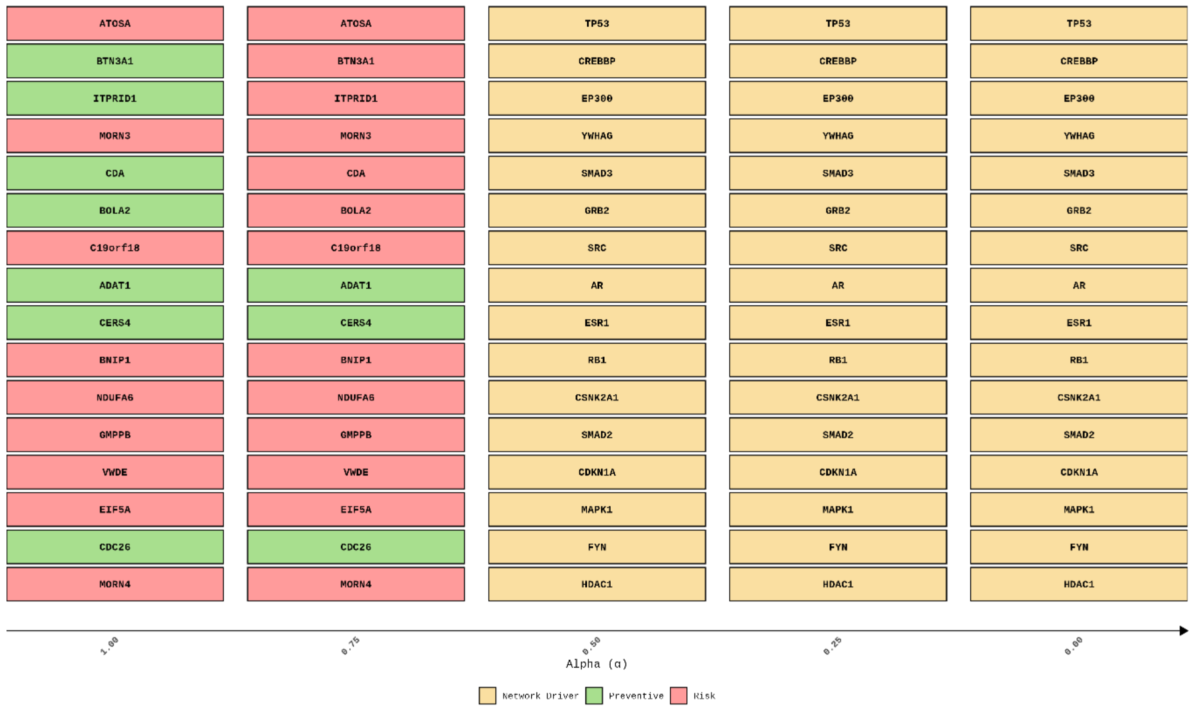

Top-16 genes across α — rin

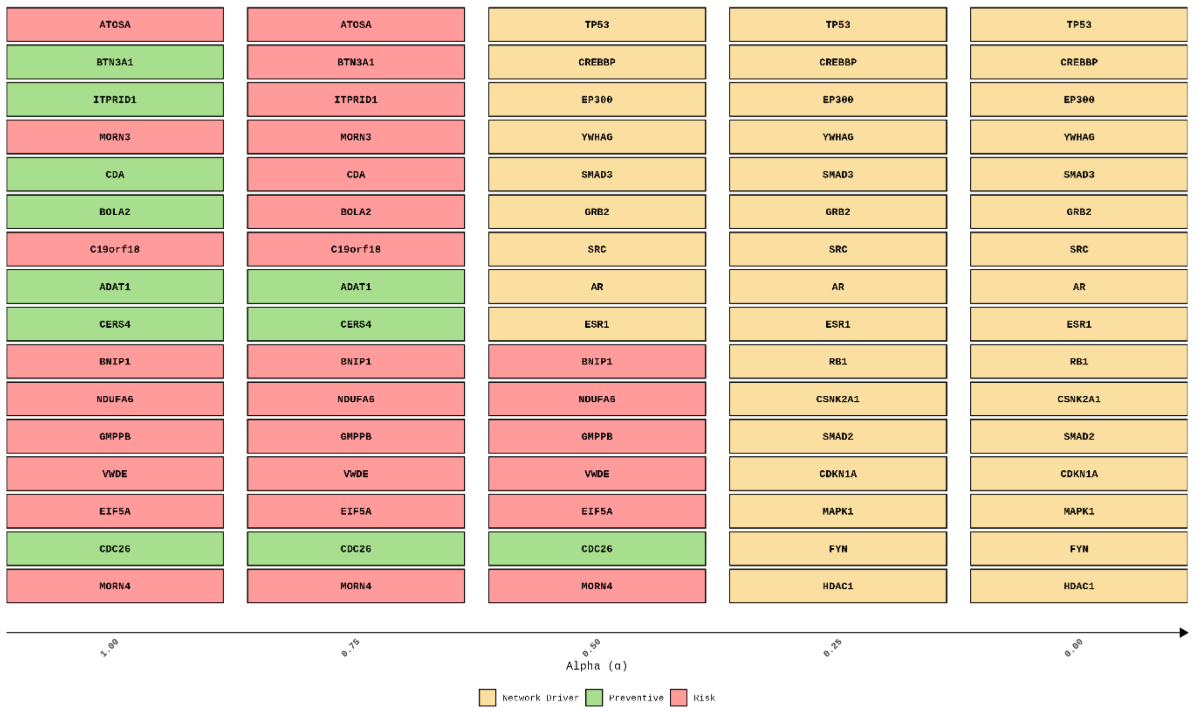

Top-16 genes across α — yeojohnson

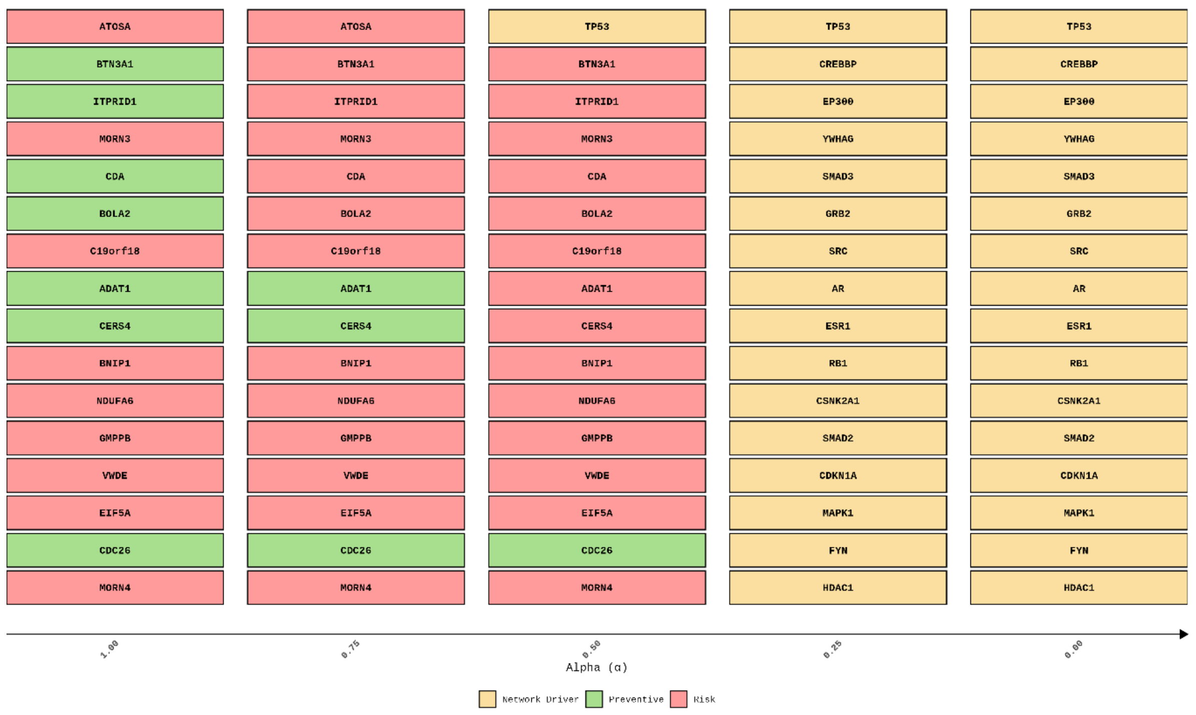

Different Normalization Techniques for the TOP 50 Genes:

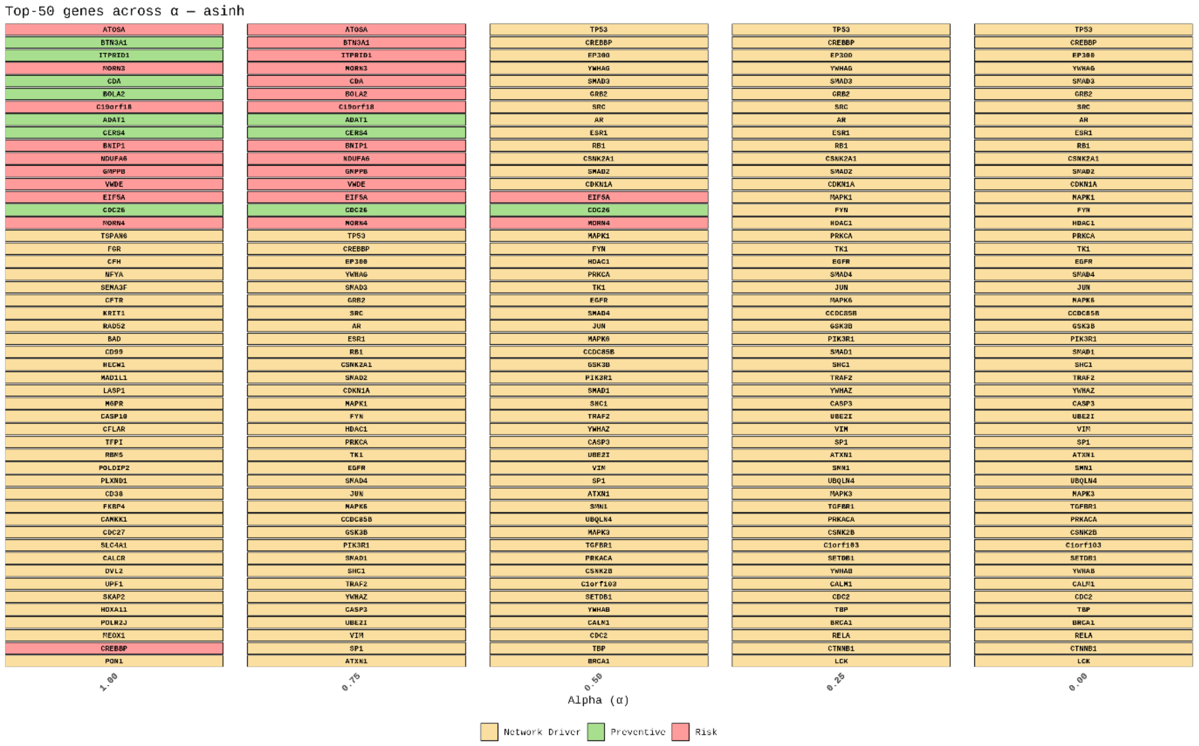

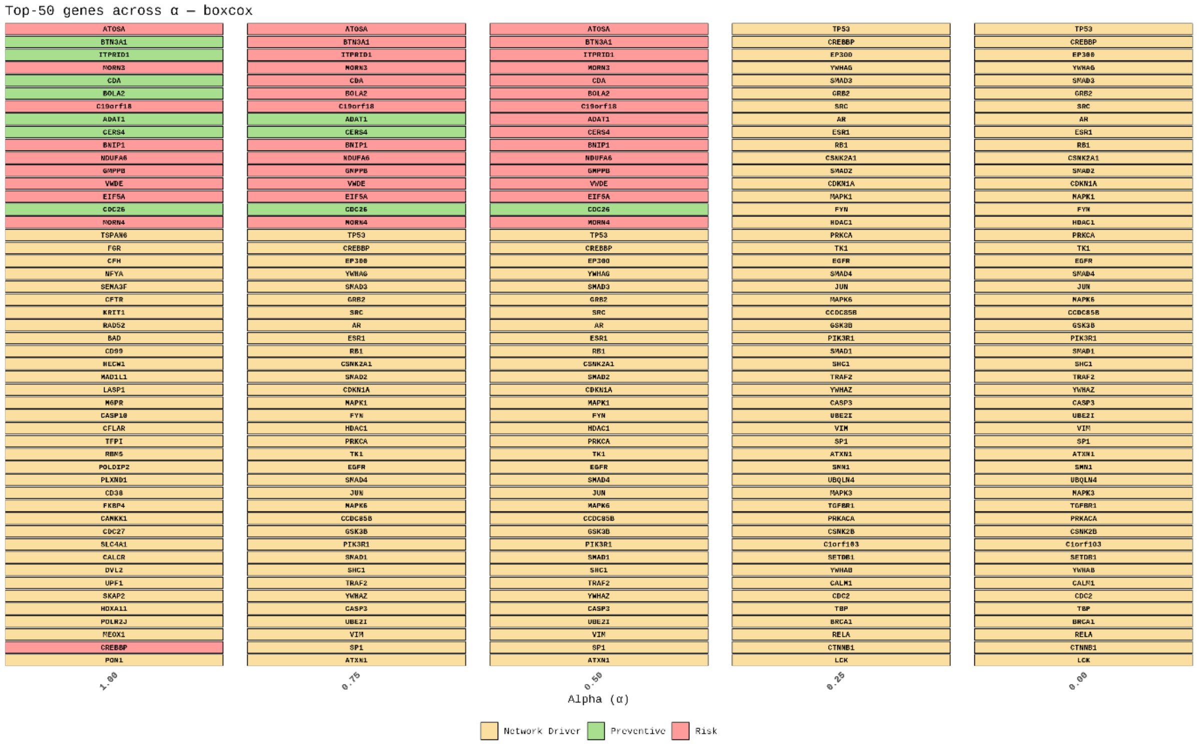

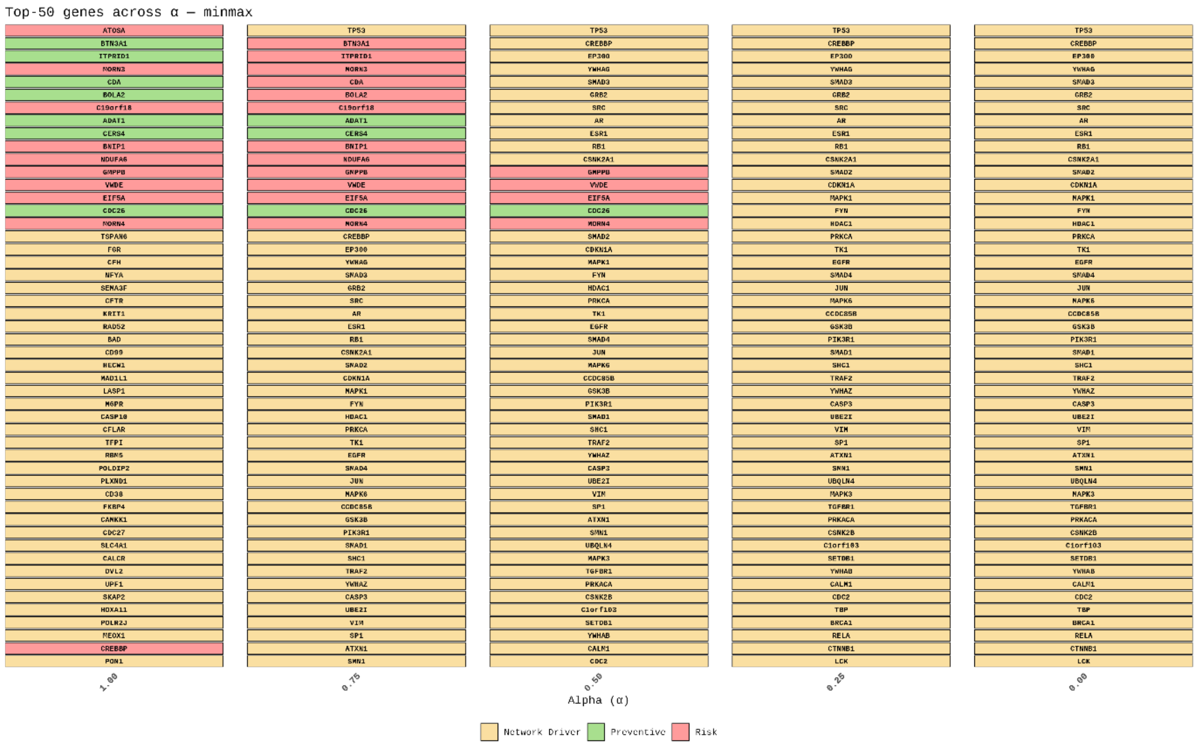
Top-50 genes across α — minmax
1.00
0.75
0.50
0.25
0.00
Alpha (α)
Network Driver
Preventive
Risk

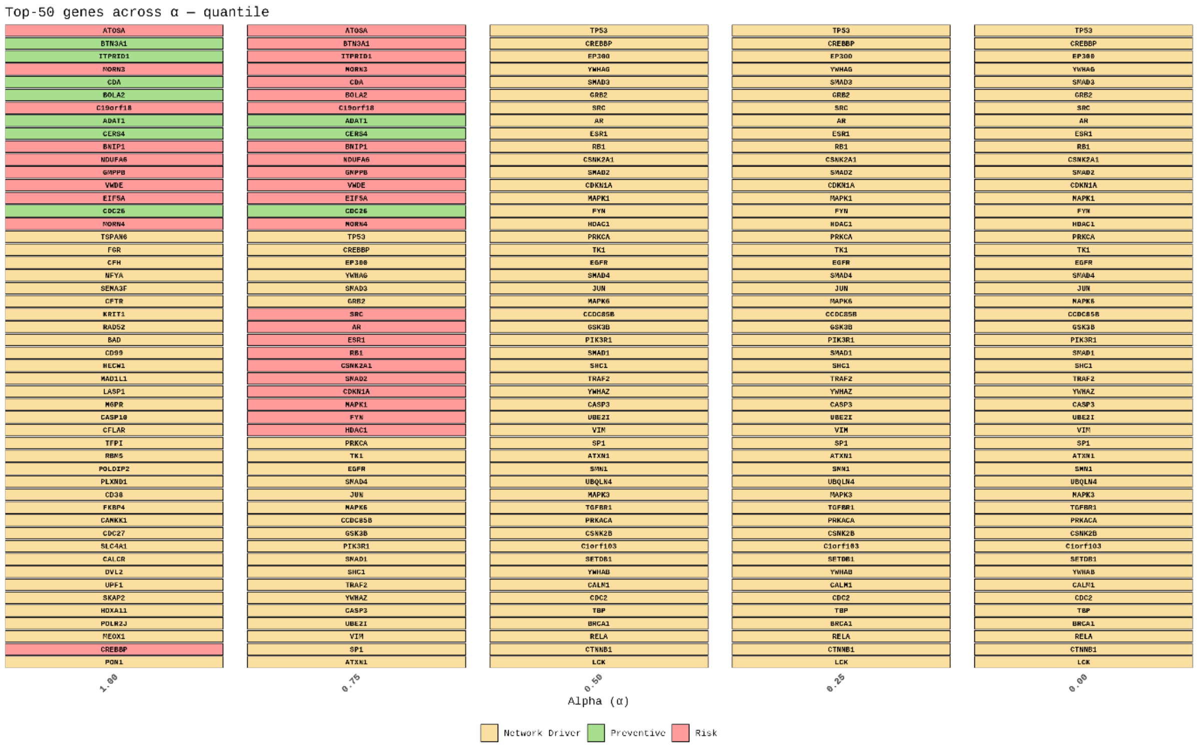
Top-50 genes across α — quantile
1.00
0.75
0.50
0.25
0.00
Alpha (α)
Network Driver
Preventive
Risk

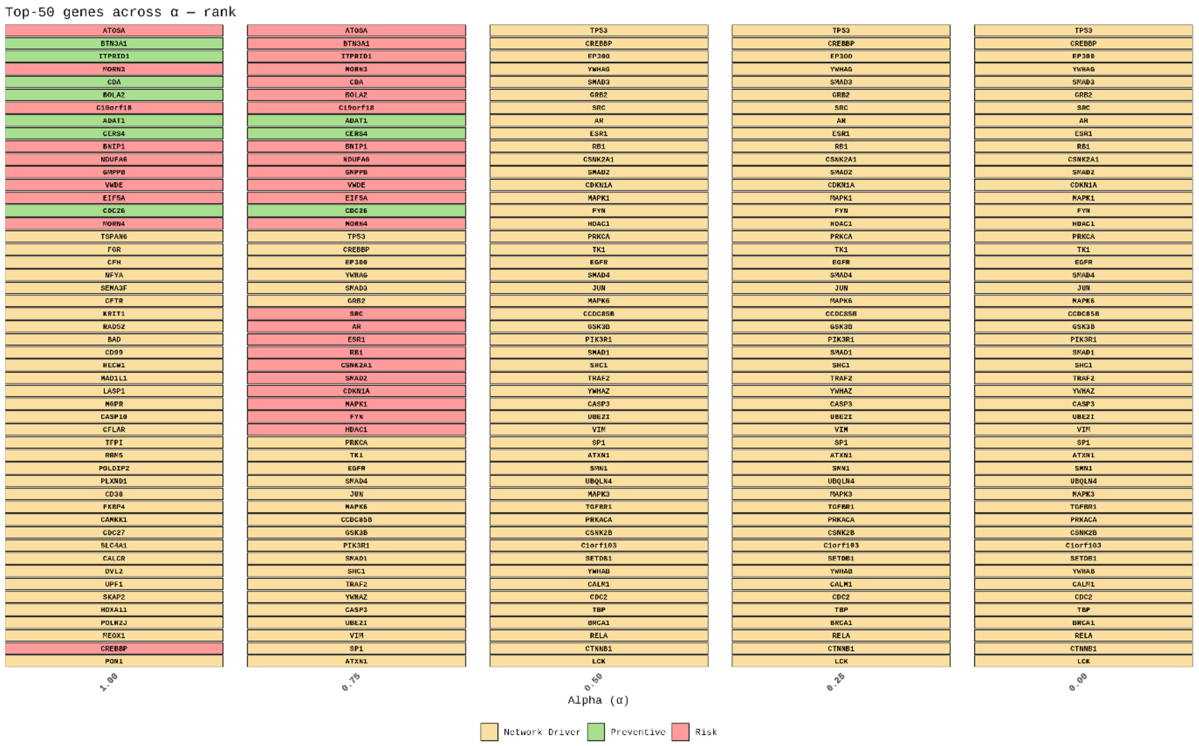
Top-50 genes across α — rank
1.00
0.75
0.50
0.25
0.00
Alpha (α)
Network Driver
Preventive
Risk

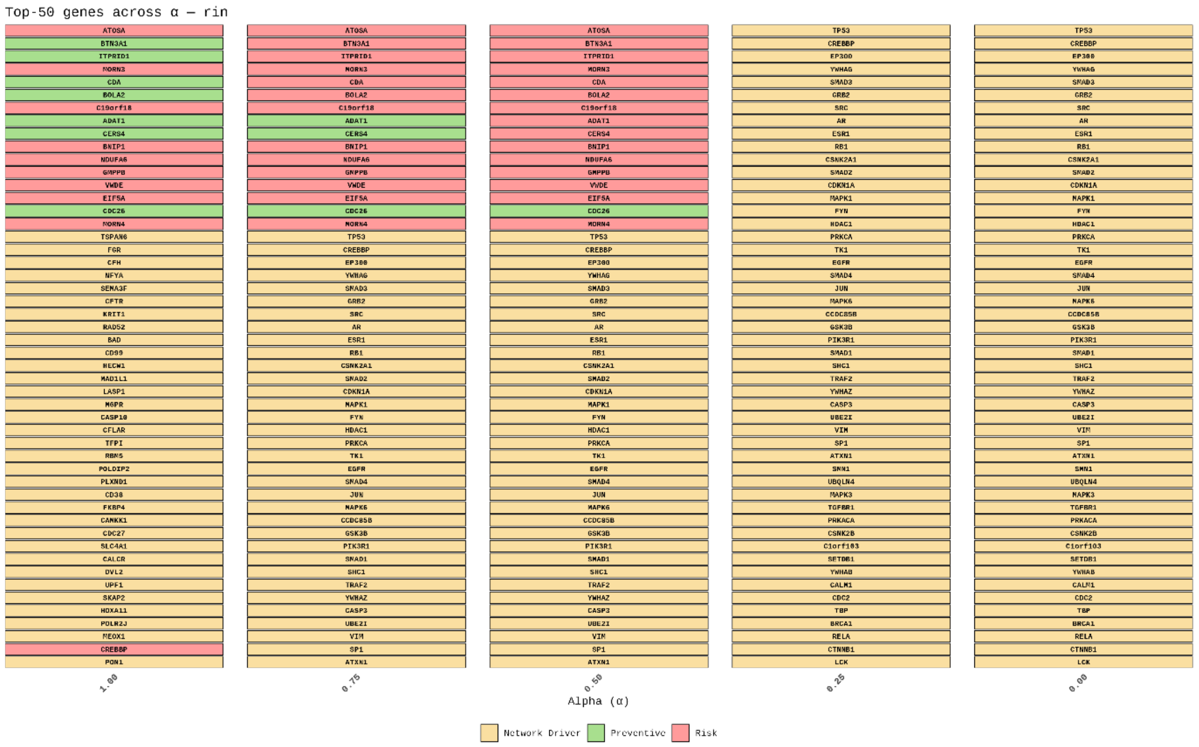
Top-50 genes across α — rin
1.00
0.75
0.50
0.25
0.00
Alpha (α)
Network Driver
Preventive
Risk

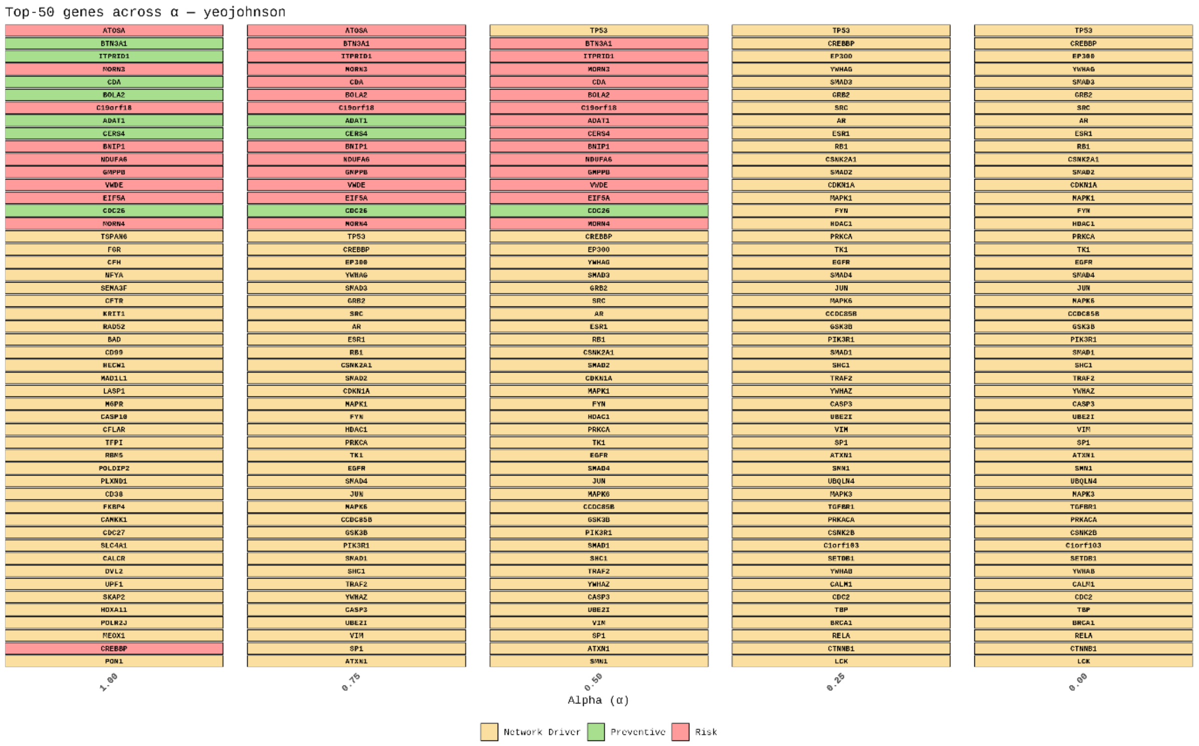
Top-50 genes across α — yeojohnson
ATOSA
BTN3A1
ITPRID1
MORN3
CDA
BOLA2
C19orf18
ADAT1
CERS4
BNIP1
NDUFA6
GMPPB
VWDE
EIF5A
CDC26
MORN4
TSPAN6
FGR
CFH
NFYA
SEMA3F
CFTR
KRIT1
RAD52
BAD
CD99
HECW1
MAD1L1
LASP1
M6PR
CASP10
CFLAR
TFPI
RBM5
POLDIP2
PLXND1
CD38
FKBP4
CAMKK1
CDC27
SLC4A1
CALCR
DVL2
UPF1
SKAP2
HOXA11
POLR2J
MEOX1
CREBBP
PON1
ATOSA
BTN3A1
ITPRID1
MORN3
CDA
BOLA2
C19orf18
ADAT1
CERS4
BNIP1
NDUFA6
GMPPB
VWDE
EIF5A
CDC26
MORN4
TP53
CREBBP
EP300
YWHAG
SMAD3
GRB2
SRC
AR
ESR1
RB1
CSNK2A1
SMAD2
CDKN1A
MAPK1
FYN
HDAC1
PRKCA
TK1
EGFR
SMAD4
JUN
MAPK6
CCDC85B
GSK3B
PIK3R1
SMAD1
SHC1
TRAF2
YWHAZ
CASP3
UBE2I
VIM
SP1
ATXN1
TP53
BTN3A1
ITPRID1
MORN3
CDA
BOLA2
C19orf18
ADAT1
CERS4
BNIP1
NDUFA6
GMPPB
VWDE
EIF5A
CDC26
MORN4
CREBBP
EP300
YWHAG
SMAD3
GRB2
SRC
AR
ESR1
RB1
CSNK2A1
SMAD2
CDKN1A
MAPK1
FYN
HDAC1
PRKCA
TK1
EGFR
SMAD4
JUN
MAPK6
CCDC85B
GSK3B
PIK3R1
SMAD1
SHC1
TRAF2
YWHAZ
CASP3
UBE2I
VIM
SP1
ATXN1
SMN1
TP53
CREBBP
EP300
YWHAG
SMAD3
GRB2
SRC
AR
ESR1
RB1
CSNK2A1
SMAD2
CDKN1A
MAPK1
FYN
HDAC1
PRKCA
TK1
EGFR
SMAD4
JUN
MAPK6
CCDC85B
GSK3B
PIK3R1
SMAD1
SHC1
TRAF2
YWHAZ
CASP3
UBE2I
VIM
SP1
ATXN1
SMN1
UBQLN4
MAPK3
TGFBR1
PRKACA
CSNK2B
C1orf103
SETDB1
YWHAB
CALM1
CDC2
TBP
BRCA1
RELA
CTNNB1
LCK
TP53
CREBBP
EP300
YWHAG
SMAD3
GRB2
SRC
AR
ESR1
RB1
CSNK2A1
SMAD2
CDKN1A
MAPK1
FYN
HDAC1
PRKCA
TK1
EGFR
SMAD4
JUN
MAPK6
CCDC85B
GSK3B
PIK3R1
SMAD1
SHC1
TRAF2
YWHAZ
CASP3
UBE2I
VIM
SP1
ATXN1
SMN1
UBQLN4
MAPK3
TGFBR1
PRKACA
CSNK2B
C1orf103
SETDB1
YWHAB
CALM1
CDC2
TBP
BRCA1
RELA
CTNNB1
LCK
1.00
0.75
0.50
0.25
0.00
Alpha (α)
Network Driver
Preventive
Risk

Different Normalization Techniques for the TOP 100 Genes:

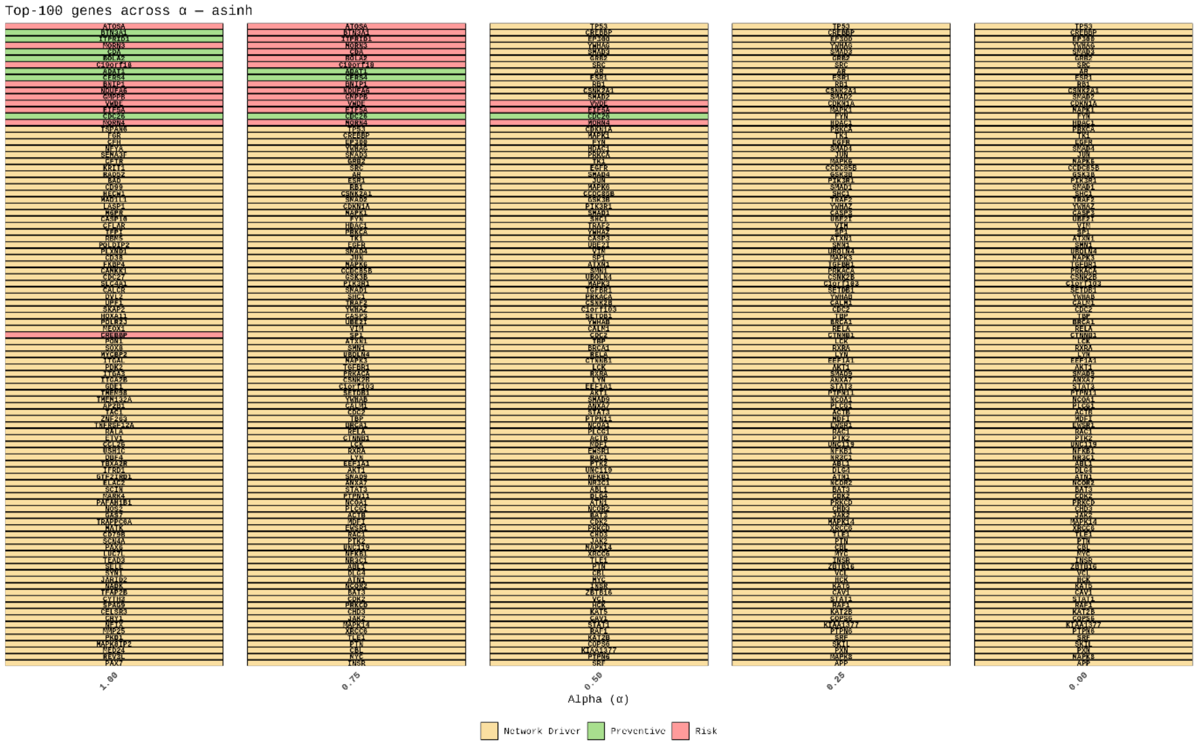

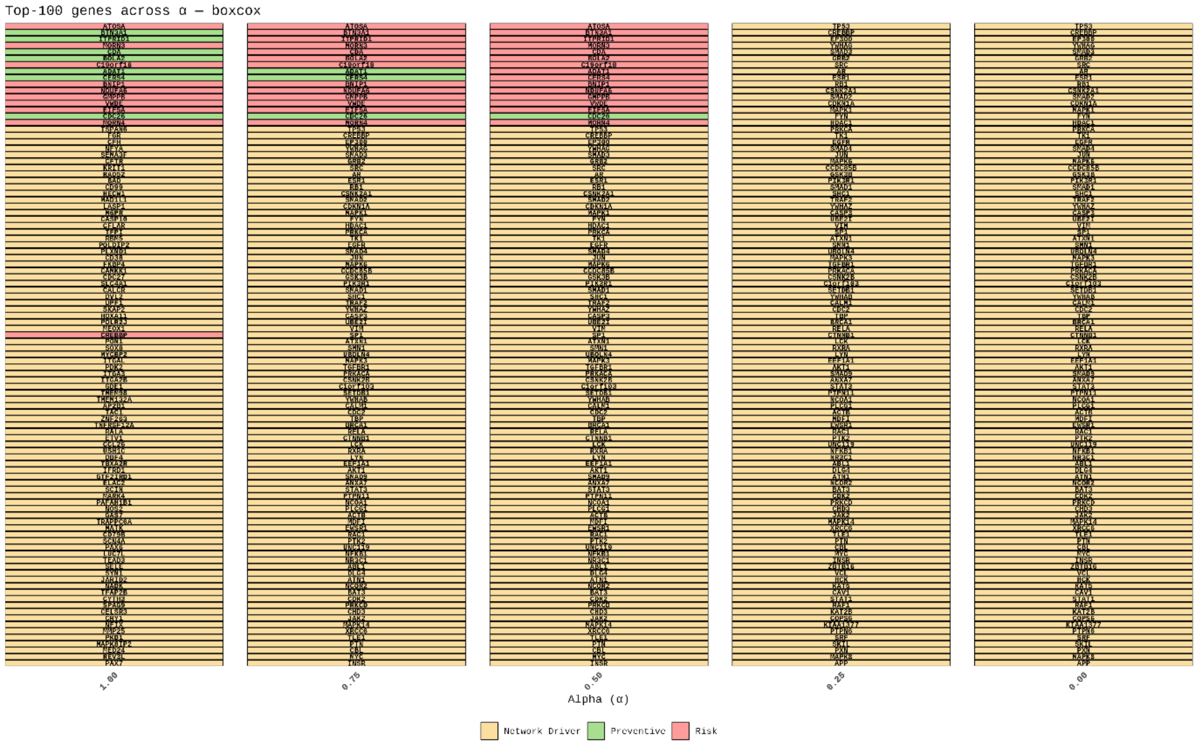

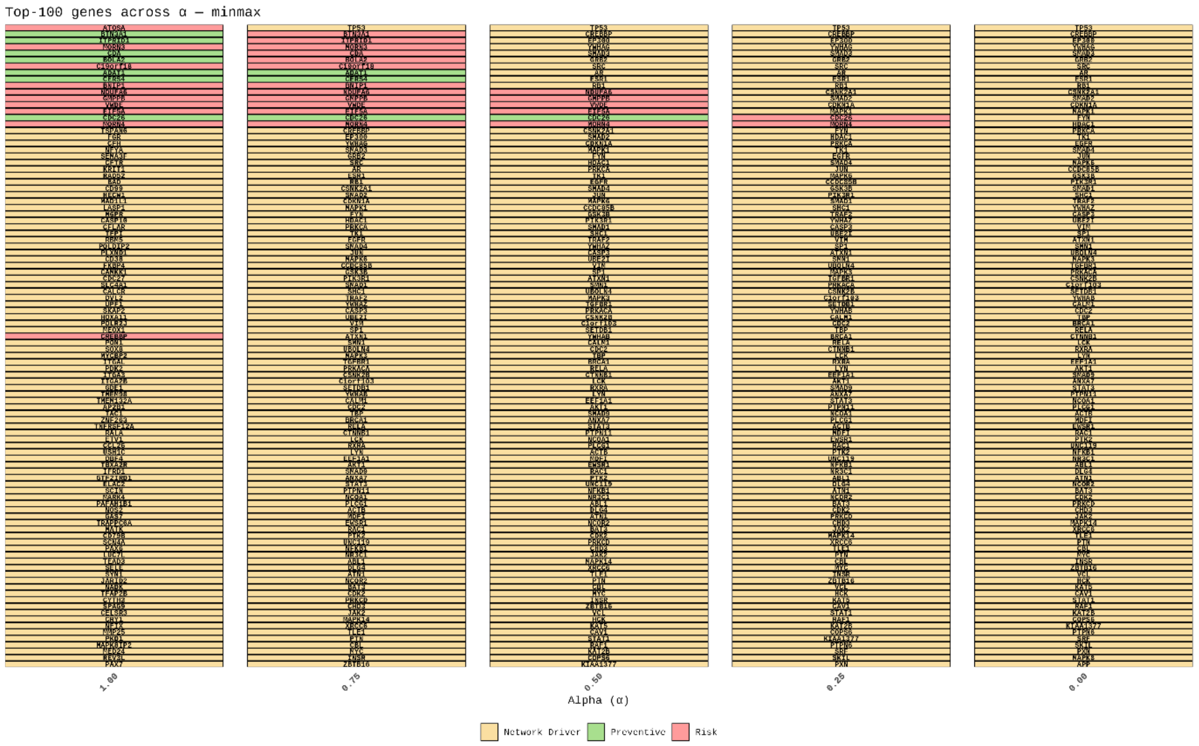
Top-100 genes across α — minmax
1.00
0.75
0.50
0.25
0.00
Alpha (α)
Network Driver
Preventive
Risk

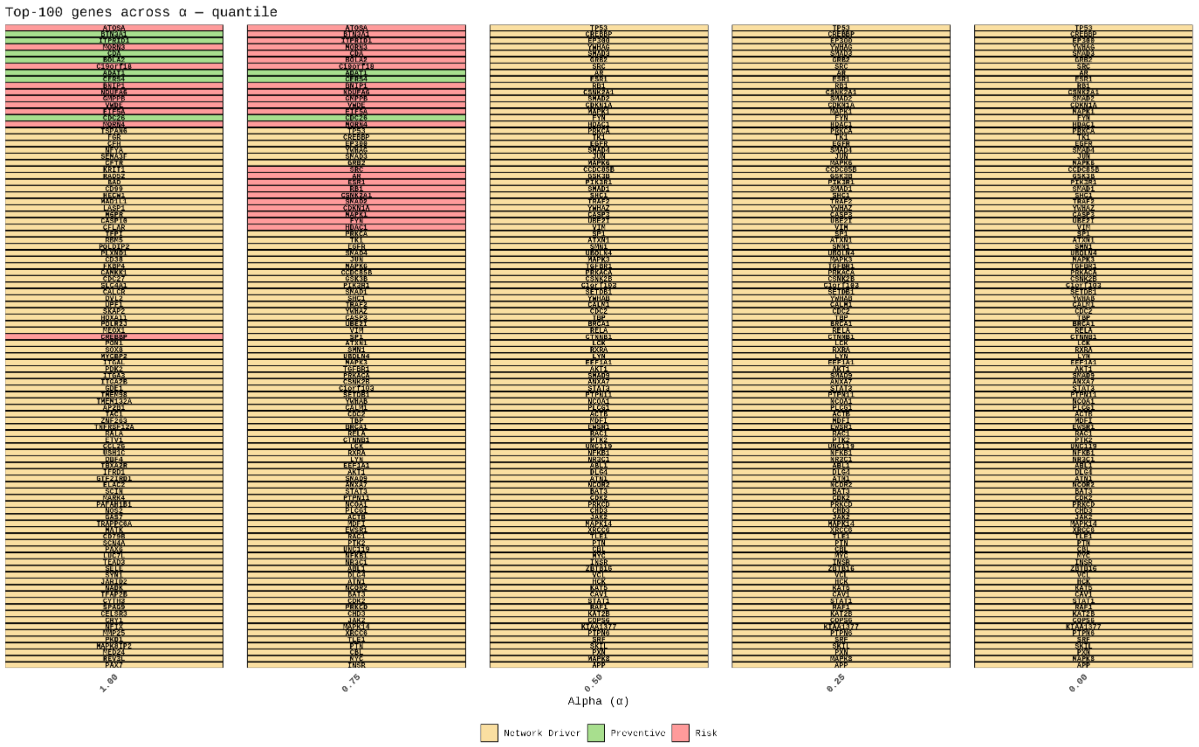
Top-100 genes across α — quantile
1.00
0.75
0.50
0.25
0.00
Alpha (α)
Network Driver
Preventive
Risk

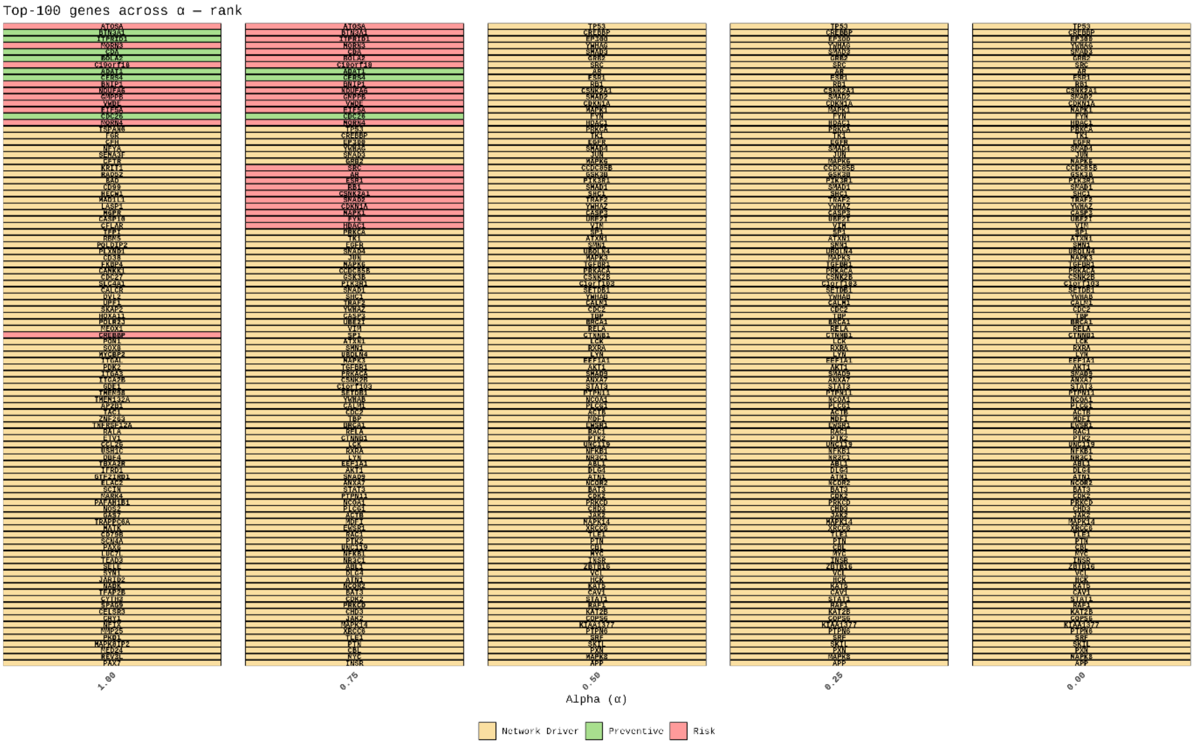
Top-100 genes across α — rank
1.00
0.75
0.50
0.25
0.00
Alpha (α)
Network Driver
Preventive
Risk

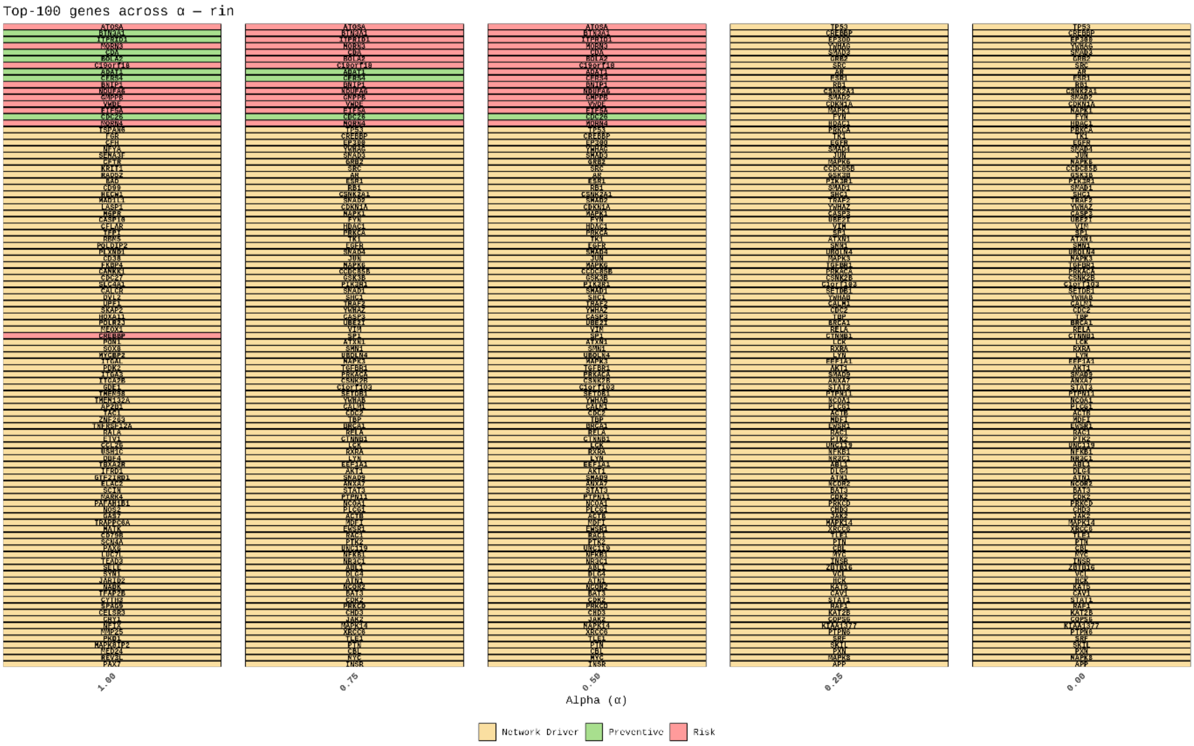
Top-100 genes across α — rin
1.00
0.75
0.50
0.25
0.00
Alpha (α)
Network Driver
Preventive
Risk

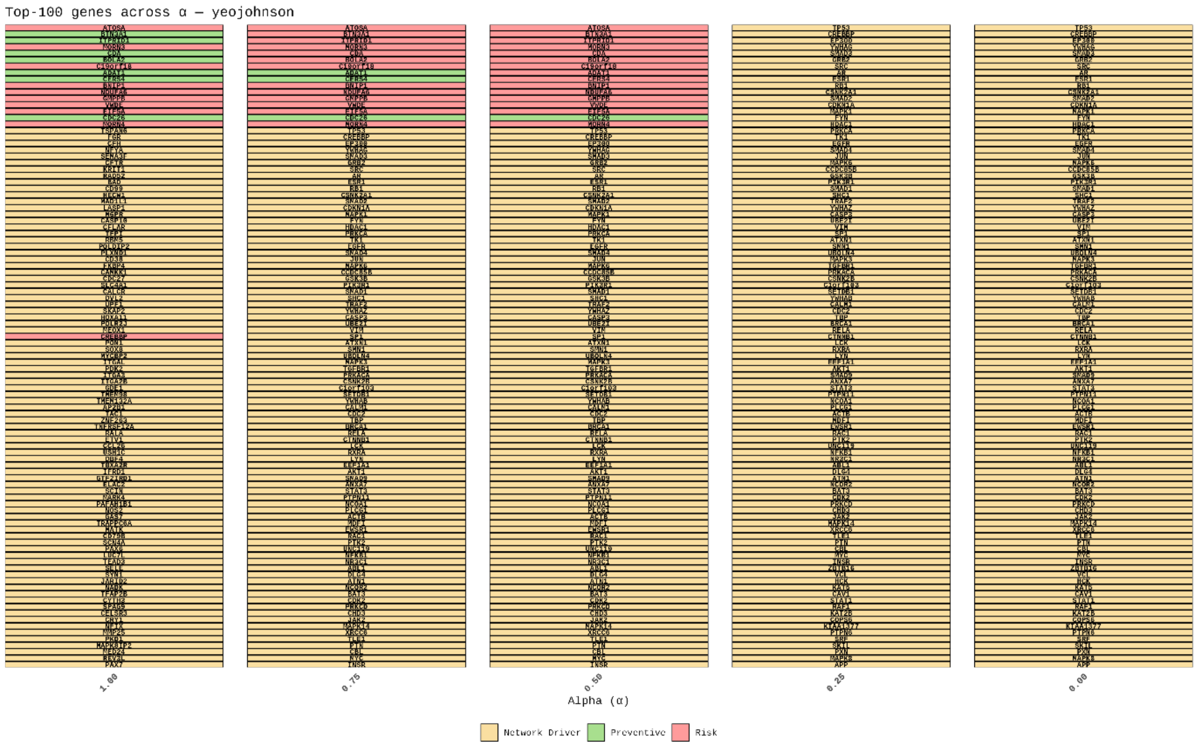
Top-100 genes across α — yeojohnson
1.00
0.75
0.50
0.25
0.00
Alpha (α)
Network Driver
Preventive
Risk
